# Evidence, strategies, and pathways for thrombectomy in medium vessel occlusion stroke: a systematic review

**DOI:** 10.3389/fneur.2026.1838008

**Published:** 2026-09-29

**Authors:** Chenye Yang, Chenyang Zhao, Yaxuan Sun

**Affiliations:** Department of Neurology, Shanxi Provincial People's Hospital, Shanxi Medical University, Taiyuan, China

**Keywords:** endovascular treatment, ischemic stroke, mechanical thrombectomy, medium vessel occlusion, patient selection, precision medicine

## Abstract

**Background:**

Medium vessel occlusions (MeVOs) account for 25–40% of acute ischemic strokes and present a significant therapeutic frontier. Despite high technical success rates of endovascular thrombectomy (EVT), recent randomized trial results have been nuanced, creating a paradox and underscoring the need for a precision-based approach.

**Methods:**

We conducted a systematic review following PRISMA guidelines. After screening, 42 original studies were included (4 RCTs and 38 cohort studies). Evidence was synthesized and appraised using a three-tiered hierarchy. The GRADE framework was applied. Overall certainty of evidence ranged from moderate to very low.

**Results:**

Observational studies, which often enrolled populations with higher baseline NIHSS scores and stricter imaging selection criteria, generally reported favorable functional independence rates (mRS 0–2 in 60–80% of EVT-treated patients). However, these rates may partly reflect selection bias rather than a purely treatment-related effect. Successful recanalization is frequently achieved (>85%), but safety concerns like symptomatic hemorrhage and vessel perforation require careful management. Success hinges on a precision strategy integrating advanced imaging (e.g., CT perfusion for “Target Mismatch”) with a tailored technical arsenal. This includes dedicated low-profile stent retrievers and aspiration catheters, selected based on occlusion site, thrombus burden, and anatomy. A “Eight Keys” framework interprets neutral trial results, highlighting patient selection heterogeneity. Future progress depends on refined imaging criteria, dedicated device development, and validated selection tools.

**Conclusion:**

Current randomized evidence does not demonstrate an overall functional benefit of endovascular thrombectomy (EVT) for unselected MeVO stroke, while procedure-related hemorrhagic risk appears higher than with best medical therapy. EVT may be reasonable in carefully selected patients with disabling deficits, salvageable tissue, and favorable anatomy, when performed by experienced teams using distal-appropriate techniques. Future work should prioritize validated selection tools, standardized imaging definitions, distal-specific devices, and trial designs enriched for patients most likely to benefit.

## 1 Introduction

Acute Ischemic Stroke (AIS) is characterized by the sudden interruption of cerebral blood flow, leading to ischemic brain injury. It remains a leading cause of mortality and adult-acquired disability worldwide, imposing a profound socioeconomic burden on healthcare systems (1). The management of AIS has been revolutionized by endovascular thrombectomy (EVT) for large vessel occlusions (LVOs). However, occlusions affecting medium-diameter intracranial vessels-medium vessel occlusions (MeVOs)-represent a substantial and evolving frontier, accounting for an estimated 25–40% of all AIS cases (2).

Anatomically, MeVO is typically defined as an occlusion of a distal intracranial artery with a lumen diameter of 0.75–2.0 mm, encompassing the M2, M3, and M4 segments of the middle cerebral artery (MCA), the A2-A4 segments of the anterior cerebral artery (ACA), and the P2-P4 segments of the posterior cerebral artery (PCA) (3). M2 occlusions are the most common and intensely studied MeVO subtype, with proximal M2 and distal M2 segments carrying distinct clinical and technical implications (4). The clinical impact of a MeVO is highly variable, depending on the occluded segment, collateral circulation, and eloquence of the downstream territory. Deficits range from mild (NIHSS 1–4) to moderate or severe (NIHSS ≥5), affecting motor, sensory, language, or cognitive function (4, 5). The anatomical complexity and access difficulty of various MeVO sites are illustrated in Figure 1 (see Discussion).

**Figure 1:**
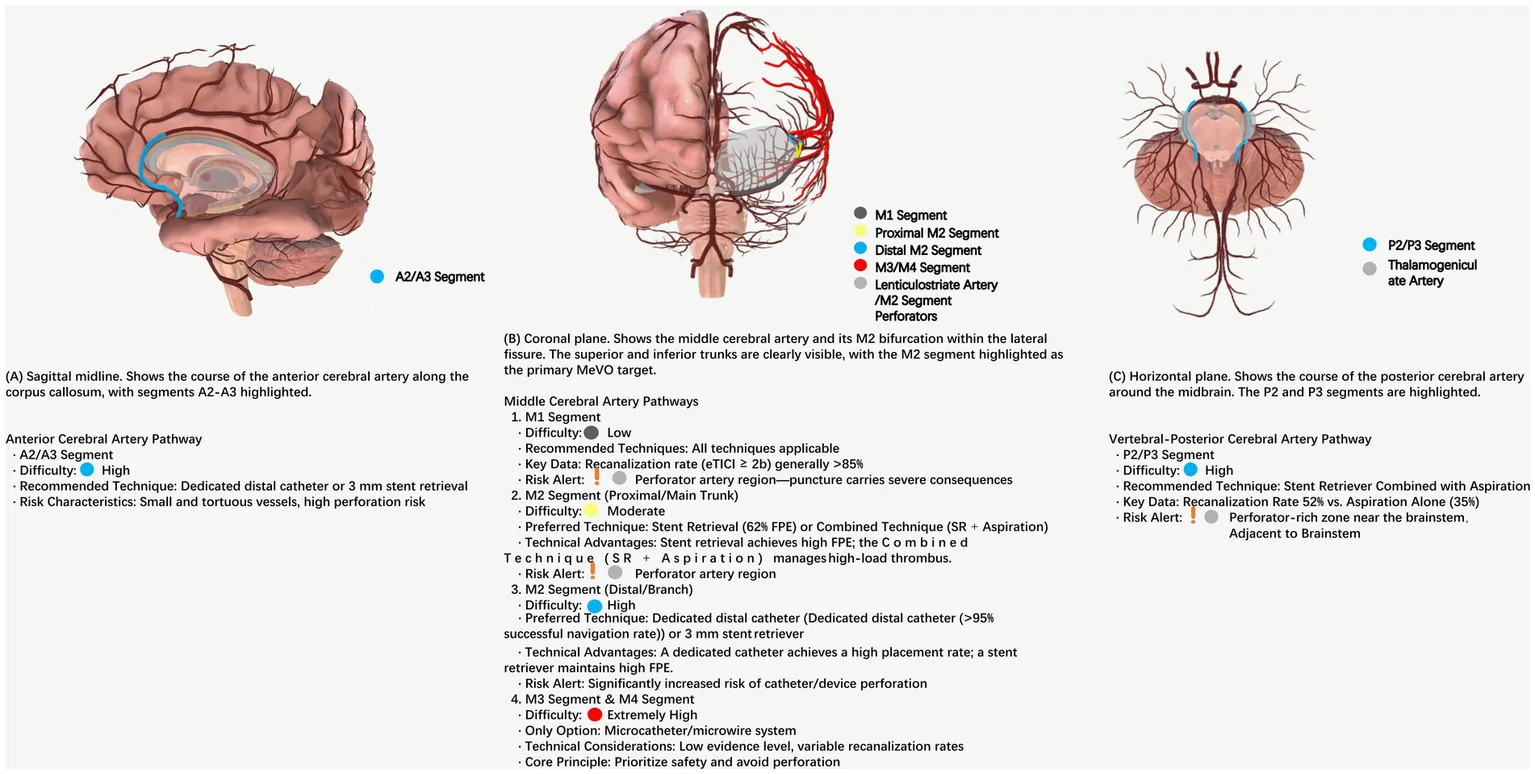
The MeVO anatomical and access difficulty landscape. BioDigital Human (BioDigital, Inc.), accessed November 18, 2023 (https://www.biodigital.com). eTICI ≥ 2b, expanded Thrombolysis in Cerebral Infarction score of 2b or greater (indicating partial to successful reperfusion); FPE, first-pass effect.

The management of MeVO necessitates a distinct approach from LVO, as they differ fundamentally across several key anatomical and pathophysiological domains (Table 1).

**Table 1:** LVO versus MeVO: a comparative analysis of imaging and pathophysiological characteristics.

Comparative dimension	LVO	MeVO	Clinical Implications
Vessel diameter	Typically >2.0 mm (e.g., ICA, M1)	Typically 0.75–2.0 mm (e.g., M2, A2, P2)	Determines device selection and technical difficulty of intervention
Perfusion territory	Extensive territories (anterior circulation via ICA/MCA, posterior via BA)	Restricted territories; M2 most common; high anatomical variability	MeVO emphasizes functional importance of infarct location over maximum potential volume
Collateral compensation	Adequacy linked to occlusion site and etiology	Crucial role of Circle of Willis and leptomeningeal flow, but assessment more challenging	No standardized collateral assessment tools for MeVO; compensatory mechanisms may differ from LVO
Ischemic Core threshold (CBV)	CBV index of 0.8 validated for clinical outcomes (63); ICAD associated with CBV index of 0.9 (64).	Optimal CBV threshold undefined (may be higher); assessment has limitations (63).	LVO thresholds are evidence-based; MeVO standards require further validation
Penumbra threshold (Tmax/CBF)	HIR (Tmax>10s / Tmax>6 s) > 0.4 predicts poor collaterals and infarct progression (53).	HIR holds predictive value but measurement of smaller lesions is more susceptible to artifact (52); optimal penumbra thresholds remain undefined	HIR applicable to both, but requires more cautious interpretation in MeVO.
Impact on clinical decision-making	Relatively standardized, based on DAWN/DEFUSE-3 criteria (e.g., core <70 mL) (65).	Complex; requires integration of core/penumbra, anatomy, symptoms, and technical feasibility. Recent RCTs have yielded mixed results, with one trial showing superiority of EVT (ORIENTAL-MeVO), two trials not demonstrating a statistically significant benefit over BMT alone (ESCAPE-MeVO, DISTAL), and one trial terminated early due to a harm signal in the EVT arm (DISCOUNT) (6–9).	Evidence level for EVT in MeVO is lower than LVO; greater individualization needed

HIR was originally developed and validated in LVO populations (DEFUSE 2 and related cohorts); its application to MeVO is extrapolative and carries greater uncertainty.

LVO, large vessel occlusion; MeVO, medium vessel occlusion; ICA, internal carotid artery; MCA, middle cerebral artery; CBV, cerebral blood volume; CBF, cerebral blood flow; Tmax, time to maximum; HIR, hypoperfusion intensity ratio; EVT, endovascular thrombectomy; ICAD, intracranial atherosclerosis; BMT, best medical therapy.

The evidence base for EVT in MeVO is in a dynamic phase of accumulation. A central paradox defines the current state: numerous observational studies report high technical success rates and favorable outcomes with EVT, yet four pivotal randomized controlled trials (RCTs)—ESCAPE-MeVO, DISTAL, and ORIENTAL-MeVO and DISCOUNT—have yielded mixed results, with only one demonstrating statistically significant superiority of EVT over best medical therapy (BMT) alone for improving 90-day functional independence (6–9). This discrepancy between technical success and clinical trial efficacy underscores the need for a precision-based approach. Table 2 summarizes the key characteristics of these RCTs.

**Table 2:** RCT summary.

Trial name	ESCAPE-MeVO (Goyal, 2025) (6)	ORIENTAL-MeVO (Hu, 2026) (8)	DISTAL (Psychogios, 2025) (7)	DISCOUNT (Clarençon, 2024) (9)
Year or status	2025	2026	2025	Terminated early (2024 protocol published)
Sample size	530 (EVT 255/Control 274)	563 (EVT 280 / Control 283)	543 (EVT 271 / BMT 272)	488 planned (actual enrollment not fully reported)
Indication	AIS due to MeVO (M2/M3, A2/A3, P2/P3)	AIS due to MeVO (codominant/nondominant M2 or M3; A1-A3; P1-P3)	AIS due to MeVO (nondominant/codominant M2-M4; A1-A3; P1-P3)	Distal M2, M3; A1-A3; P1-P3
Treatment window	≤12 h from LKW	≤24 h from LKW	≤24 h from LKW (perfusion selection for 6–24 h)	≤6 h from LKW (≤24 h if FLAIR negative)
Key inclusion criteria	NIHSS >5 or 3–5 with disabling deficit; salvageable tissue on imaging	NIHSS ≥6; core <50% of territory or mismatch ratio >1.4	NIHSS ≥4 or disabling deficit; prestroke independent	NIHSS ≥5; distal occlusion
Intervention	EVT (first-line Solitaire X) + usual care vs. usual care alone	EVT (any device/technique) + medical management vs. medical management alone	EVT (any device/technique) + BMT vs. BMT alone	EVT (stent retriever ≤3 mm and/or aspiration) + BMT vs. BMT alone
Primary outcome	mRS 0–1 at 90d	mRS 0–2 at 90d	Ordinal shift in mRS at 90d	mRS 0–2 at 90d
Primary outcome results	EVT 41.6% vs. Control 43.1%; aRR 0.95 (0.79–1.15), *p* = 0.61	EVT 58.6% vs. Control 46.6%; aRR 1.24 (1.07–1.44), *p* = 0.004	cOR 0.90 (0.67–1.22), *p* = 0.50	Stopped early: interim analysis showed harm signal (worse functional outcomes, higher mortality, more sICH in EVT arm)
Actually included in this review	Yes	Yes	Yes	Yes

mRS, modified Rankin scale; NIHSS, National Institutes of Health Stroke Scale; MCA, Middle Cerebral Artery; PCA, Posterior Cerebral Artery; ACA, Anterior Cerebral Artery; eTICI, expanded Thrombolysis in Cerebral Infarction; ASPECTS, Alberta Stroke Program Early CT Score; BMM/BMT, best medical management/therapy; IVT, intravenous thrombolysis; EVT, endovascular therapy/mechanical thrombectomy; sICH, symptomatic intracranial hemorrhage.

Given the smaller vessel caliber, greater tortuosity, and higher perforation risk associated with MeVO thrombectomy. Success hinges on two interconnected pillars: precision patient selection using advanced imaging and refined interventional technique with dedicated low-profile devices. This systematic review aims to synthesize the current evidence on EVT for MeVO, critically appraise the discrepancies between randomized and observational data, and provide a structured framework for individualized decision-making.

## 2 Method

### 2.1 Study design

This systematic review was conducted following the Preferred Reporting Items for Systematic Reviews and Meta-Analyses (PRISMA) reporting guidelines. The methods were predefined and are described in detail to ensure transparency and reproducibility.

This systematic review was not prospectively registered in PROSPERO or any other registry. Key methodological decisions—including eligibility criteria, outcome measures, and analysis plans—were, however, prespecified by the authors in a study protocol before data extraction commenced. The review was conducted and reported in accordance with the PRISMA 2020 guidelines.

### 2.2 Search strategy

We conducted a systematic search of three electronic databases: PubMed/MEDLINE, Embase, and Web of Science. The search covered records from inception to May 2026. The search strategy combined Medical Subject Headings (MeSH) terms and free-text keywords related to the core concepts. The primary search block focused on the condition, using terms such as “medium vessel occlusion”, “MeVO”, “distal occlusion”, “M2 occlusion”, “M3 occlusion”, and “A2 occlusion.” This was combined (using the Boolean operator “AND”) with a second block targeting the intervention, including terms such as “endovascular thrombectomy”, “mechanical thrombectomy”, “thrombectomy”, and “aspiration thrombectomy.” No language restrictions were applied. The complete search strategies for all three databases, including all MeSH terms, free-text keywords, and database-specific syntax, are provided in Supplementary Table S1.

The database search yielded 776 records (PubMed: 211, Embase: 309, Web of Science: 256). An additional 9 records were identified through manual screening of reference lists and relevant systematic reviews (snowball searching), bringing the total to 785 records. After removing 400 duplicates, 385 unique records were screened.

### 2.3 Eligibility criteria

Studies were considered eligible if they met all of the following criteria:

1. Study design: Randomized controlled trials (RCTs), prospective or retrospective cohort studies, or case–control studies.
2. Population: Adult patients (≥18 years) with acute ischemic stroke confirmed by neuroimaging to be due to a medium vessel occlusion (MeVO), defined as occlusion of distal intracranial arteries with a diameter <2 mm (e.g., M2-M4 segments of the middle cerebral artery, A2-A4 segments of the anterior cerebral artery, or P2-P4 segments of the posterior cerebral artery).
3. Intervention: Endovascular thrombectomy (EVT) as a primary or adjunctive treatment, using any device or technique.
4. Comparator: Best medical management (including intravenous thrombolysis) or no comparator for single-arm studies.
5. Outcomes: Studies reporting at least one of the predefined efficacy (e.g., 90-day functional independence, successful reperfusion) or safety outcomes (e.g., symptomatic intracranial hemorrhage, vessel perforation).

Systematic reviews and meta-analyses were not included as primary studies; instead, their reference lists were manually screened to identify any additional eligible original studies (see 2.2).

Studies were excluded if they were narrative reviews, editorials, conference abstracts, technical notes, animal studies, or focused exclusively on large vessel occlusions (LVOs) without separate MeVO analysis. Non-English publications without a reliable translation were also excluded. The full inclusion and exclusion criteria, including detailed definitions and rationales, are provided in Supplementary Tables S2, S3.

### 2.4 Study selection and data extraction

Study selection was performed independently by two reviewers. Titles and abstracts were screened against the eligibility criteria, and potentially relevant records were retrieved for full-text assessment. The same two reviewers independently evaluated the full-text articles for inclusion. Disagreements at any stage were resolved through discussion, or by consultation with a third reviewer when consensus could not be reached.

Data from included studies were extracted into a standardized, pre-piloted template by one reviewer. The extracted data were independently verified for accuracy and completeness by a second reviewer. Any discrepancies were resolved through discussion. We acknowledge that this verification-based approach, while systematic, does not constitute fully independent dual extraction. It may therefore introduce a risk of transcription bias. This is addressed as a limitation in the Discussion section. Extracted information included study characteristics (first author, year, design, sample size), patient population (MeVO definition, occlusion site, baseline severity), intervention details (device, technique, anesthesia), efficacy outcomes, and safety outcomes.

For the purposes of this review, a “disabling deficit” was operationally defined as any neurological deficit that significantly impairs a patient’s occupational capacity or activities of daily living, conceptually corresponding to a modified Rankin Scale (mRS) score of ≥2–3. This definition was applied consistently when interpreting study eligibility criteria (e.g., “NIHSS 3-5 with disabling deficit”) and when categorizing clinical severity in the proposed scoring system (Supplementary Table S5).

The primary outcome of interest was 90-day functional independence, defined as a modified Rankin Scale (mRS) score of 0 to 2. Secondary outcomes were categorized into efficacy and safety endpoints. Efficacy endpoints comprised successful reperfusion (expanded Thrombolysis in Cerebral Infarction grade of 2b50 or greater, i.e., reperfusion of ≥50% of the involved territory); first-pass effect (complete or near-complete reperfusion, eTICI 2c-3, with a single device pass without rescue therapy); early neurological improvement (reduction of ≥8 points on the National Institutes of Health Stroke Scale or an NIHSS score of 0–1 at 24 h post-procedure) (10) and follow-up infarct volume (the volume of infarcted tissue on imaging, usually DWI or NCCT, performed 24–36 h after treatment). Safety endpoints included symptomatic intracranial hemorrhage, defined according to the Heidelberg Bleeding Classification as any intracranial hemorrhage associated with a ≥ 4-point increase in NIHSS score or leading to death (unless a study explicitly used an alternative standardized definition); vessel perforation (iatrogenic rupture of an intracranial artery during the procedure, confirmed angiographically by active contrast extravasation); embolization to new territory (angiographically confirmed migration of thrombus to a previously uninvolved vascular territory during the procedure); 90-day all-cause mortality; and procedure-related complications including arterial dissection, subarachnoid hemorrhage, and transient catheter-induced vasospasm. Variability in sICH definitions across studies was noted as a limitation. Detailed definitions and the clinical significance of these metrics are provided in Supplementary Table S6.

### 2.5 Risk of bias assessment

The quality of individual studies was assessed using appropriate tools based on study design. Randomized controlled trials (RCTs) were evaluated using the Cochrane Risk of Bias tool (RoB 2). Cohort studies and case–control studies were assessed with the Risk Of Bias In Non-randomized Studies of Interventions (ROBINS-I) tool. Each study was independently assessed by two reviewers. Disagreements were resolved through discussion or consultation with a third reviewer.

Domain-level ratings for all included studies are provided in Supplementary Table S14 (ROBINS-I) and Supplementary Table S16 (RoB 2), with corresponding traffic light plots in Supplementary Figures S3, S4. A concise summary of overall risk of bias judgements is presented in Table 3.

**Table 3:** Summary of evidence levels for included studies.

Evidence level	Tier	Number of studies
High	Tier I (RCTs)	4 (ESCAPE-MeVO, DISTAL, ORIENTAL-MeVO, DISCOUNT)
Moderate	Tier II (Observational)	~35
Low	Tier III (Observational)	~3

For study-level detail, see Supplementary Table S8.

### 2.6 Data synthesis and analysis

Data were synthesized qualitatively without meta-analysis. This was due to substantial heterogeneity in study designs, populations, and outcome measures across included studies. Narrative synthesis was structured around key clinical and technical themes. Findings were summarized in Tables 4–7, and in Supplementary Table S8.

**Table 4:** Systematic comparison of baseline characteristics between pivotal RCTs and representative observational studies in MeVO thrombectomy.

Characteristic	ESCAPE-MeVO (*n* = 530)	DISTAL (*n* = 543)	ORIENTAL-MeVO (*n* = 563)	DISCOUNT (*n* = 488 planned)	Observational studies (pooled estimates)	Key difference
Baseline NIHSS, median	7 (5–10)	6 (5–9)	8 (6–11)	≥5 (inclusion criterion)	9–14	Observational cohorts enroll patients with higher stroke severity
Age, median	75	77	71	Not fully reported	68–76	Comparable, though some observational cohorts include younger patients
Imaging selection	Non-contrast CT/MRI (ASPECTS) or CTA (collaterals) or perfusion (mismatch)	CTA/MRA; perfusion required for 6–24 h window	CTA/MRA; perfusion mismatch required (penumbra/core >1.4)	CT or MRI (local standard); FLAIR negativity required for 6–24 h window	Predominantly CTA + CT perfusion	RCTs used heterogeneous imaging criteria; observational studies more consistently applied perfusion imaging
Core infarct volume limit	No strict limit (absence of extensive ischemic changes)	No strict limit (presence of salvageable tissue)	<50% of at-risk territory	Not specified in protocol	Variable; commonly <50–70 mL	RCTs used more lenient or heterogeneous core criteria
Time window	≤12 h	≤24 h (perfusion selection for 6–24 h)	≤24 h	≤6 h (≤24 h if MRI FLAIR negative)	≤24 h	Comparable
IVT rate	57.60%	65.40%	36.60%	Not fully reported	22–74%	Highly variable across studies
Successful reperfusion (eTICI ≥2b)	75.10%	71.70%	74.40%	Not fully reported	85–90%	Higher in observational cohorts, reflecting operator experience and device evolution
Dominant M2 included?	Yes	No (excluded)	Yes (codominant)	No (proximal M2 excluded by anatomical landmark)	Variable	DISTAL excluded the subgroup with potentially greatest treatment benefit

This table compares key baseline characteristics of the three pivotal RCTs with representative pooled estimates from observational studies included in this review. The observed differences, particularly in baseline NIHSS, imaging selection criteria, and the inclusion of dominant M2 occlusions, provide empirical context for the hypothetical framework presented in Table 7. RCT data are derived from (6–8); observational estimates are based on the studies summarized in Supplementary Table 15. Additional empirical support for selective enrollment in RCTs is provided by Murillo-Olaizola et al. (50), who reported that trial participants had significantly lower baseline NIHSS (5 vs 10) and smaller hypoperfusion volumes (27 mL vs 48 mL) compared with patients treated in routine clinical practice.

The overall certainty of evidence was assessed using the GRADE framework. This assessment considered five domains: risk of bias, inconsistency, indirectness, imprecision, and publication bias. Risk of bias ratings from Section 2.5 directly informed this assessment. These ratings were based on RoB 2 for RCTs and ROBINS-I for non-randomized studies. Certainty was rated as high, moderate, low, or very low for each key outcome. The results of this assessment are presented in Section 3.4.

## 3 Results

### 3.1 Study selection and characteristics

The electronic database search yielded a total of 776 records. This included 211 records from PubMed, 309 from Embase, and 256 from Web of Science. An additional 9 records were identified through manual screening of reference lists and relevant systematic reviews. This brought the total to 785 records before deduplication. After removing 400 duplicates, 385 unique records were screened at the title and abstract level. Of these, 339 records were excluded based on predefined eligibility criteria. The remaining 46 reports were sought for full-text retrieval, and all were obtained. Following full-text assessment, 4 additional reports were excluded. Consequently, 42 original studies were included in this systematic review (4 RCTs and 38 observational studies). The flow of study selection is detailed in the PRISMA diagram (Figure 2).

**Figure 2:**
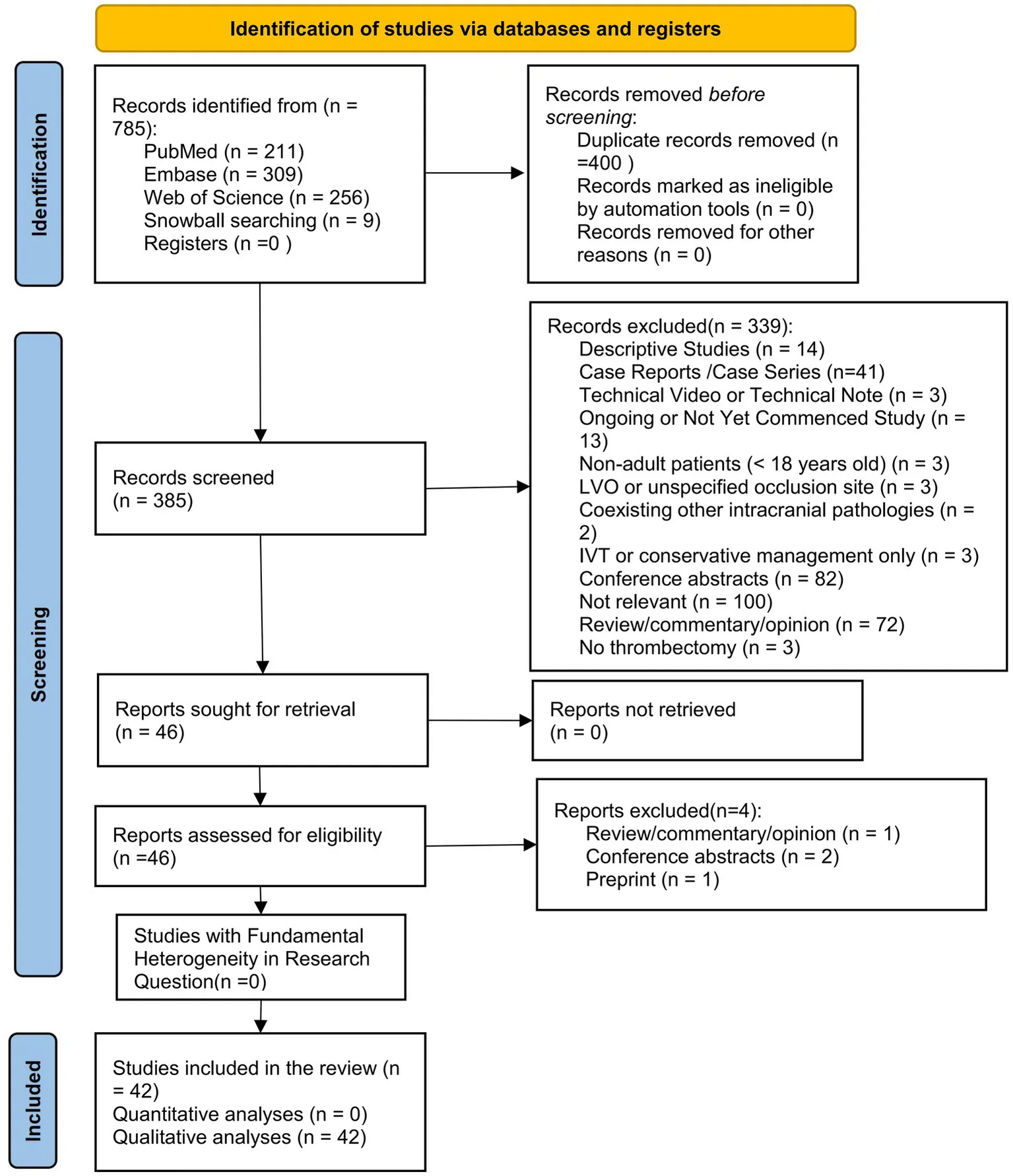
PRISMA flow diagram of the study selection process.

Among the 42 original studies, the evidence base comprised four pivotal RCTs (Tier I) and 38 observational studies (Tiers II and III). The Tier I RCTs—ESCAPE-MeVO, DISTAL, ORIENTAL-MeVO, and DISCOUNT—were all prospective, multicenter, open-label trials with blinded endpoint assessment. Each directly compared EVT combined with best medical therapy (BMT) against BMT alone in patients with acute MeVO. The treatment windows varied: up to 12 h from last known well for ESCAPE-MeVO, up to 24 h for both DISTAL and ORIENTAL-MeVO, and up to 6 h (or ≤24 h if MRI FLAIR negative) for DISCOUNT (6–9). Notably, the DISCOUNT trial was terminated early after a pre-specified interim analysis suggested worse functional outcomes, higher mortality, and increased symptomatic intracranial hemorrhage in the EVT group. The remaining 38 observational studies comprised the Tier II and III evidence base. Large-scale registries, including the MAD-MT Registry, TOPMOST study, ANGEL-ACT registry, and German Stroke Registry, provided robust real-world data on outcomes, techniques, and complications (11–13). Numerous single-center and multicenter cohort studies investigated specific technical aspects and dedicated devices, such as Trevo NXT, APERIO Hybrid, and Catch Mini. Additional studies examined the prognostic value of imaging biomarkers, including the Hypoperfusion Intensity Ratio (HIR) (5, 14–17).

The key characteristics of all 42 original studies are systematically summarized in Table 5. These characteristics include study design, sample size, patient population, imaging protocols, specific interventions, and primary outcomes. Complete details are provided in Supplementary Table S15.

**Table 5:** Characteristics of included studies.

Study	Design	MeVO sample size (EVT),	Occlusion sites included	EVT technique(s) used	Primary outcome (90d mRS 0–2)	sICH rate (%)	Overall risk of bias
Abecassis, 2026 (69)	Multicenter retrospective cohort	257	A1/A2, M2, M3/4, P1/2	ADAPT, SR, Combined	64.6%	6.9%	Moderate (ROBINS-I)
Alessiani, 2025 (70)	Retrospective observational study (single center)	59	A2, A3, distal M2 or its single branch, M3, P2, P3, PICA, SCA, AICA	Direct aspiration (ADAPT)	50.9% (calculated: 100–49.1% with mRS > 2)	33.8% (hemorrhagic transformation, not strictly sICH)	ROBINS-I: Moderate
Clarençon, 2024 (9)	Phase 3, multicenter, prospective, randomized, open-label, blinded-outcome (PROBE) trial—terminated early	488 planned; actual enrollment not fully reported	Distal M2, M3; A1-A3; P1-P3	Stent retriever (≤3 mm) and/or aspiration catheter (operator discretion); maximum 3 passes	Not reached; trial stopped early after interim analysis showed harm signal (worse functional outcomes, higher mortality, more sICH in EVT arm)	Not fully reported; interim analysis indicated increased sICH in EVT arm	RoB 2: Some concerns (incomplete outcome data due to early termination)
Dmytriw, 2024 (21)	Retrospective multicenter cohort (37 centers, propensity-score matched)	670 (335 MT alone + 335 MT + IVT)	Medium vessel occlusion per Saver et al. (includes M2, M3, A2, A3, P2, P3, etc.)	Aspiration and/or stent retriever (operator discretion)	aOR for MT + IVT vs. MT alone = 1.37 (95% CI: 0.99–1.89), *p* = 0.054 (not significant)	Not reported as sICH; any ICH aOR = 1.25 (0.88–1.76), *p* = 0.21	ROBINS-I: Moderate
Gaub, 2026 (72)	Retrospective multicenter registry (STAR)	1734 (711 aspiration, 958 SR/combined)	M2 segment (trunk, frontal division, temporal division)	Frontline aspiration vs. stent retriever alone or combined	PSM: aspiration 49.9%, SR/combined 44.0%; OR 1.27 (1.03–1.57)	PSM: aspiration 3.5%, SR/combined 6.2%; OR 0.55 (0.33–0.91)	ROBINS-I: Moderate
Goyal, 2025 (6)	Phase 3, prospective, randomized, open-label, blinded-outcome (PROBE) trial	255	M2 (proximal/distal), M3, A2, A3, P2, P3	First-line Solitaire X stent retriever (± aspiration); subsequent attempts any device	54.1% (138/255) - secondary outcome; primary outcome was mRS 0–1 (41.6% EVT vs. 43.1% control, not significant)	5.4% (14/257 as-treated)	RoB 2: Low
Grossberg, 2024 (71)	International multicenter retrospective registry (STAR)	1,287 (pMeVO cohort); propensity-matched for technique comparison: 295 ADAPT, 225 SRT/PC	M2, A1, P1	Frontline aspiration (ADAPT) vs. stent retriever (SRT) vs. primary combined (PC)	Matched cohort: ADAPT 45%, SRT/PC 47% (*p* > 0.05) - no significant difference between techniques	Matched cohort: ADAPT 4%, SRT/PC 9% (*p* < 0.01) - significantly lower with ADAPT	ROBINS-I: Moderate
Ishiguro, 2026 (85)	Prospective single-center before-after cohort	76 (Tron group); 121 (conventional SR group); MeVO subgroup numbers not separately reported for outcome	M2, M3, ACA, PCA (MeVO); also includes M1 and ICA	Combined technique (SR + aspiration + balloon guide catheter). Tron FX II (low radial/axial force) vs. conventional SRs (Solitaire X, Trevo NXT, EmboTrap III)	No significant difference between Tron and conventional SR groups (32.9% vs. 25.6%, *p* = 0.330)	Not reported as sICH; symptomatic SAH (neurological deterioration) in MCA + MeVO: Tron 1.9% vs. conventional 12.7% (*p* = 0.044)	ROBINS-I: Serious (non-randomized before-after design, potential time-period bias, small MeVO subgroup)
Hu, 2026 (8)	Multicenter, prospective, randomized, open-label, blinded-outcome (PROBE) trial	280	M2 (codominant/nondominant, ≤2 mm), M3, A1-A3, P1-P3	Operator discretion: stent retriever, aspiration, combined (most common), intraarterial thrombolysis, angioplasty	58.6% (164/280) vs. control 46.6%; adjusted rate ratio 1.24 (1.07–1.44), *p* = 0.004	4.7% (13/275) vs. control 2.2% (6/271); adjusted risk ratio 2.21 (0.87–5.63)	RoB 2: Low
Hu, 2026 (8)	Multicenter retrospective registry (China, 25 centers)	529	M3, M4; A1-A3; P1-P3 (excludes M2)	Operator discretion: combined (47.2%), aspiration only (30.9%), stent retriever only (21.9%)	EVT 60.9% (322/529) vs. SMM 53.5% (292/546); adjusted RR 1.39 (1.14–1.69), *p* = 0.001	EVT 11.5% (61/529) vs. SMM 10.4% (57/546); adjusted RR 1.24 (0.92–1.66), *p* = 0.16	ROBINS-I: Moderate
Meyer, 2024 (12)	International, retrospective, multicenter registry (TOPMOST)	233 (119 LA ± CS, 114 GA)	PCA: P2/P3; ACA: A2-A4	Aspiration, stent retriever, intra-arterial thrombolysis (operator discretion)	90-day mRS 0–1: LA ± CS 63.9%, GA 40.2% (unadjusted *p* = 0.002; mRS 0–2 not reported)	LA ± CS 2.5%, GA 1.8% (*p* = 1.0)	ROBINS-I: Moderate
Meyer, 2021 (12)	International, retrospective, multicenter case–control (propensity-matched)	92 (after PSM)	PCA: P2, P3	Stent retriever (± aspiration) 72%, primary aspiration 26.6%, intra-arterial tPA 1.4%	90-day mRS 0–2: EVT 76.6% (59/77), medical management 75.4% (43/57); *p* = 0.87 (not significant)	EVT 4.3% (4/92), medical management 4.3% (4/92); *p* > 0.99	ROBINS-I: Moderate
Meyer, 2023 (12)	International, retrospective, multicenter case–control (propensity-matched)	55 (after PSM; total EVT 94)	ACA: A2, A3, A4 (distal to A1)	Stent retriever (± aspiration), primary aspiration, intra-arterial thrombolysis	EVT 49% (18/37), medical 49% (19/39); *p* = 0.99 (not significant)	EVT 2% (2/94), medical 2% (1/60); overall 2%	ROBINS-I: Moderate
Meyer, 2022 (12)	International, retrospective, multicenter registry (TOPMOST subanalysis)	141	PCA: P2, P3	Aspiration only (29%) vs. stent retriever (71%); combined aspiration ± SR allowed	90d mRS 0–2 not reported; 90d mRS 0–1: aspiration 60.5%, stent retriever 68.6% (*p* = 0.40)	2.8% (4/141) overall; aspiration 2.4%, stent retriever 3.0%	ROBINS-I: Moderate
(Mohammaden, 2025) (34)	Multicenter retrospective propensity-matched analysis (14 centers)	122 (61 GA, 61 non-GA) after matching	MCA-M3/4, ACA-A2/3, PCA-P1/P2-3	SR alone, CA alone, combined (SR + CA), IA-tPA, wire maceration	GA 45.5% (25/55), non-GA 43.6% (24/55); OR 1.14 (0.44–2.96), *p* = 0.79	GA 15.5% (9/58), non-GA 4.5% (3/56); OR 3.24 (0.83–12.68), *p* = 0.09	ROBINS-I: Moderate
DUSK (Mohammaden, 2024) (34)	Multicenter retrospective cohort with IPTW (7 centers)	179	MCA-M3/M4, ACA-A2/A3, PCA-P1/P2 (excludes M2, A1, P3)	Operator discretion: stent retriever, aspiration, combined, etc.	EVT 53.6% (96/179), MM 60.6% (86/142); adjusted OR 1.39 (0.80–2.45), *p* = 0.244	EVT 5.6% (10/179), MM 6.3% (9/142); aOR 0.57 (0.21–1.58), *p* = 0.277	ROBINS-I: Moderate
PLATO (Nguyen, 2024) (73)	International multicenter retrospective case–control study (30 sites)	326	PCA: P1, P2, P3, fetal, bilateral (isolated PCA occlusion)	Stent retriever (SR) alone, contact aspiration (CA) alone, combined (SR + CA)	50.0% (145/290) overall; SR 60.0%, CA 47.3%, combined 49.0% (unadjusted; adjusted: SR vs. CA aOR 0.52 favoring SR)	5.6% (18/326) overall (SR 7.0%, CA 5.8%, combined 5.1%; *p* = 0.892)	ROBINS-I: Moderate
Orscelik, 2024 (74)	Retrospective multicenter registry (STAR)	BGC 231, non-BGC 1277 (total 1,508)	MCA M2-M4, ACA any segment, PCA any segment (DMVO per consensus)	ADAPT, stent retriever, Solumbra, etc. (operator discretion)	BGC 38.6%, non-BGC 45.2% (*p* = 0.11)	BGC 4.2%, non-BGC 5.1% (*p* = 0.68)	ROBINS-I: Moderate
Palmisano, 2024 (75)	Multicenter retrospective review of prospective database (5 Italian centers)	171 (DA 95, CT 76)	M2, M2/M3, M3, M4, A2 (anterior circulation DMVO)	Direct aspiration (DA) vs. combined technique (CT: aspiration + stent retriever); all used JET D catheter	DA 63.7% (58/91), CT 36.1% (26/72); *p* = 0.001	Overall hemorrhagic complications: DA 7.4% vs. CT 18.4% (*p* = 0.035); parenchymal hematoma (sICH surrogate): DA 4.2% vs. CT 10.5% (*p* = 0.136)	ROBINS-I: Moderate
Pérez-García, 2020 (22)	Single-center retrospective analysis (prospectively maintained database)	102 patients (49 mini SR, 53 BEMP)	MCA mid/distal M2, M3; ACA A1-A3; PCA P1/distal	BEMP (mini stent retriever + low-profile DAC) vs. mini stent retriever alone	BEMP 50.9% (27/53), mini SR 53.1% (26/49); *p* = 0.831	BEMP 1.9% (1/52)*, mini SR 12.8% (5/39)*; *p* = 0.038	ROBINS-I: Moderate
Palmisano, 2024 (75)	Multicenter retrospective review of prospective database (5 Italian centers)	171 (DA 95, CT 76)	M2, M2/M3, M3, M4, A2 (anterior DMVO, 90% M2)	Direct aspiration (DA) vs. combined technique (CT: aspiration + stent retriever); all used JET D catheter (0.054″)	DA 63.7% (58/91), CT 36.1% (26/72); *p* = 0.001	Parenchymal hematoma (sICH surrogate): DA 4.2% (4/95), CT 10.5% (8/76); *p* = 0.136	ROBINS-I: Moderate
Rizzo, 2024 (76)	Prospective single-center observational study	38 (EVT-only)	Distal M2, M3; A2, A3; P2, P3	Operator-dependent (stent retriever, aspiration, or combined)	31.6% (12/38)	2.6% (1/38)*	ROBINS-I: Moderate
Rosi, 2025 (77)	Retrospective before-after cohort (single center)	100 (3D-RA 57, control 43)	MCA M2 segment	Aspiration alone or combined (stent retriever + aspiration)	3D-RA 49.1% vs. Control 45% (*p* = 0.834)	3D-RA 5.3% (3/57), control 2.3% (1/43); *p* = 0.697	ROBINS-I: Moderate
Saber, 2022 (67)	Multicenter retrospective cohort with IPTW (11 US centers)	156	MCA M3, ACA (any segment)	Aspiration alone 49.0%, stent retriever (± aspiration) 43.5%, IA alteplase 7.5%	51.7% (weighted) vs. MM 44.0%; aOR 1.36 (0.84–2.19), *p* = 0.20	EVT 4.0% (weighted), MM 3.1%; *p* = 0.90	ROBINS-I: Moderate
Salim, 2026 (56)	Multicenter retrospective propensity-matched cohort (37 centers)	184 (IAT 19, MT 165)	MCA M2, M3, M4	IAT (urokinase) vs. MT (aspiration/stent retriever/combined)	IAT 50% (9/18), MT 40% (64/160); *p* = 0.42	MT 6.7% (10/150), IAT 0% (0/18); *p* = 0.60	ROBINS-I: Moderate
Salim, 2024 (51)	Multicenter retrospective propensity-matched cohort (37 centers)	320 (MT-IVT)	M2, P1, A1 (medium) and M3/M4, P2/P3, A2/A3 (distal) - all DMVO	Aspiration 23%, combined 64%, stent retriever 13% (first-line)	MT-IVT 62% (197/320), IVT alone 63% (202/320); adjusted OR 1.00 (0.66–1.51), *p* > 0.99	MT-IVT 8.3% (26/320), IVT alone 6.9% (22/320); adjusted OR 0.78 (0.42–1.45), *p* = 0.44	ROBINS-I: Moderate
Salim, 2025 (56)	Multicenter retrospective propensity-matched cohort (37 centers)	391	MCA M2, M3, M4	Aspiration 20%, combined 69%, stent retriever 11%	EVT 59% (187/391), BMM 59% (206/391); adjusted OR 1.00 (0.67–1.50), P > 0.99	EVT 8.4% (31/391), BMM 3.0% (11/391); adjusted OR 3.56 (1.69–7.48), *p* < 0.001	ROBINS-I: Moderate
Salsan, 2024 (15)	Retrospective, two-center, IPTW-adjusted cohort	50	M2 (distal/non-dominant), M3, M4, A2/A3, P2/P3	Contact aspiration 62%, stent retriever (± aspiration) 38%	EVT 74.0% (37/50), IVT 65.6% (42/64); adjusted OR 2.02 (0.85–4.85), *p* = 0.12	EVT 6.0% (3/50), IVT 9.4% (6/64); adjusted OR 0.49 (0.10–2.39), *p* = 0.38	ROBINS-I: Moderate
Siegler, 2025 (78)	International multicenter retrospective propensity-matched cohort (37 centers, 11 countries)	522 (Trevo 261, other 261)	M2, M3; A1, A2; P1, P2 (proximal medium and distal medium)	Trevo (combined with aspiration in 88.8%) vs. other devices (aspiration, other stent retrievers, etc.)	Trevo 52.5% (137/261), other 46.7% (122/261); adjusted OR 1.32 (0.86–2.01), *p* = 0.20	Not reported separately; parenchymal hematoma: Trevo 9.5%, other 7.9% (*p* = 0.60)	ROBINS-I: Moderate
Siegler, 2023 (79)	International multicenter retrospective propensity-matched cohort (37 centers, 10 countries)	210 (after matching: aspiration 105, stent-retriever 105)	M2, P1, A1 (proximal medium); M3, P2, A2, others (distal medium)	Aspiration alone vs. stent-retriever alone (no combined techniques)	Aspiration 57.1% (129/226), stent-retriever 55.4% (108/195) (unadjusted *p* = 0.73); adjusted OR 1.00 (0.87–1.15)	Not explicitly reported; any ICH: aspiration 25.3%, stent-retriever 34.2% (adjusted OR 1.10, 0.96–1.25)	ROBINS-I: Moderate
Zhang, 2026 (68)	Multicenter retrospective registry with PSM and IPTW (25 Chinese centers)	138 (after PSM)	ACA A1, A2, A3	Aspiration 28.7%, combined 60.7%, stent retriever 10.7%	PSM: 65.2% vs. SMM 50%; adjusted RR 2.17 (1.23–3.83), *p* = 0.007	Not separately reported; no significant difference between groups (aRR 1.12, *p* = 0.793)	ROBINS-I: Moderate
Nedelcu, 2023 (80)	Single-center retrospective cohort	39	M2-M4, A2-A3, P1-P2, PICA, AICA, SCA	Operator discretion (aspiration, stent retriever, or combined)	69.2% (27/39) vs. BMT 70.7% (29/41); OR 0.56 (0.17–1.83), *p* = 0.341	12.8% (5/39) parenchymal hemorrhage (PH1 + PH2); sICH not separately reported	ROBINS-I: Moderate
Shimonaga, 2025 (81)	Single-center retrospective cohort	134	M2 segment	SR (41%), CA (31%), CT (28%)	50.8% (68/134) overall; no significant difference between techniques across angles	2.2% (3/134) overall	ROBINS-I: Moderate
Hu, 2026 (8)	Single-center retrospective cohort with IPTW	54 (EVT) / 172 (conservative treatment) - after IPTW: EVT 97, conservative treatment 145	M2-M4, A1-A3, P1-P3	SR (Solitaire, EmboTrap), aspiration (SOFIA, 3MAX/4MAX, ACE60), or combined	After IPTW: EVT 68.0% vs. conservative treatment 67.6% (*p* = 0.998) - no significant difference	EVT 8.2% vs. conservative treatment 0% (*p* = 0.002)	ROBINS-I: Moderate
Alawieh, 2022 (82)	International multicenter retrospective registry (STAR)	213 (DVO subgroup)	MCA M3/M4, ACA A2/A3, PCA P2/P3	ADAPT, stent retriever (SR), primary combined (PC)	DVO overall: 44%; no significant difference between techniques (after PSM: ADAPT 47% vs. SR/PC 47%)	DVO overall: 5–6%; no significant difference between techniques	ROBINS-I: Moderate
Elhorany, 2022 (83)	Single-center retrospective cohort with PSM	44 (MT + BMT); after PSM: 28	44 (MT + BMT);after PSM: 28	Stent retriever (≤3 mm) ± aspiration; 3MAX aspiration	After PSM: MT + BMT 53.6% (15/28), BMT 57.1% (16/28); OR 0.87 (0.3–2.4), *p* = 1.00	After PSM: MT + BMT 3.6% (1/28), BMT 0%; P = 1.00	ROBINS-I: Moderate
Nagel, 2020 (84)	Single-center retrospective cohort	30 (EVT ± IVT)	M2, M3, A2, P1, P2	Stent retriever (90%), aspiration (10%)	73.3% (22/30) (good outcome: mRS 0–2 or return to baseline) vs. IVT 87.5% (56/64), *p* = 0.14	13.3% (4/30) (HBC class 2/PH2) vs. IVT 1.6% (1/64), *p* = 0.01	ROBINS-I: Moderate
Mehta, 2025 (37)	Single-center retrospective cohort	279 (CS 193, GA 69)	M2-M4, A1-A2, P1-P2	Aspiration (72%), stent retriever ± aspiration (24%)	Not reported; 90-d good outcome defined as mRS 0–3: CS 41.7%, GA 34.4% (*p* = 0.12)	ICH (PH1/2): CS 2.2%, GA 4.2% (*p* = 0.51)	ROBINS-I: Moderate
Valente, 2024 (35)	Multicenter retrospective PSM (16 centers)	94 (after PSM: GA 47, CS/LA 47)	M2 segment (superior, middle, inferior branches)	Stent retriever ± aspiration, or direct aspiration	GA 87.2%, CS/LA 80.8% (*p* = 0.401) - secondary outcome, no significant difference	sSAH: GA 2.2%, CS/LA 13.5% (*p* = 0.082)	ROBINS-I: Moderate
Hofmeister, 2025 (45)	Single-center retrospective cohort	72 (CE-CBCT 37, 3DRA 35)	M2 segment	Combined technique (stent retriever + aspiration)	No significant difference: median mRS 2 in both groups (*p* = 0.770)	Not separately reported; complications low, no difference	ROBINS-I: Moderate
Berberich, 2025 (36)	International multicenter retrospective cohort (PLATO) with IPTW	376 (non-GA 193, GA 183)	PCA: P1, P2, P3, fetal PCA	Stent retriever, contact aspiration, combined (operator discretion)	Non-GA 50.0% (91/182), GA 47.7% (73/153) (unadjusted *p* = 0.677); IPTW OR 0.84 (0.50–1.39), *p* = 0.49	Non-GA 7.8% (15/193), GA 5.0% (9/179); IPTW OR 0.54 (0.17–1.76), *p* = 0.31	ROBINS-I: Moderate
DISTAL (Psychogios, 2025) (7)	Phase 3, multicenter, prospective, randomized, open-label, blinded-outcome (PROBE) trial	271	Nondominant/codominant M2, M3/M4; A1/A2/A3; P1/P2/P3 (excludes dominant M2)	Combined (64.6%), stent retriever only (15.7%), aspiration only (15.7%)	90-day mRS 0–2 rate not directly reported; median mRS 2 vs. 2 (IQR both 1–4); common OR for shift 0.90 (0.67–1.22), *p* = 0.50	EVT 5.9% (16/271), BMT 2.6% (7/272); OR 2.38 (0.44–6.14)	RoB 2: Low

BEMP, balloon-guided enhanced microcatheter protection; BGC, balloon guide catheter; BMT, best medical management/best medical therapy/medical management; CBV, cerebral blood volume; CE-CBCT, contrast-enhanced cone-beam CT; CS, conscious sedation; CTA, CT angiography; CTP, CT perfusion; DSA, digital subtraction angiography; DRA, digital subtraction angiography (Note: typically synonymous with DSA); END, early neurological deterioration; SR, stent retriever; CA, contact aspiration; ENT, embolization to new territory; eTICI, expanded thrombolysis in cerebral infarction; EVT, endovascular therapy/mechanical thrombectomy; FIV, final infarct volume; FPE, first-pass effect; GA, general anesthesia; HIR, hypoperfusion intensity ratio; IVT, intravenous thrombolysis; LA, local anesthesia; MRA, MR angiography; NCCT, non-contrast CT.

*sICH rates calculated after excluding patients who required rescue therapy.

### 3.2 Clinical and angiographic outcomes

Synthesizing findings across the three-tiered evidence hierarchy reveals a complex picture of the efficacy and safety of MeVO thrombectomy. A detailed, study-by-study overview of efficacy and safety outcomes is provided in Supplementary Table S8. A concise summary of evidence levels is presented in Table 3.

A critical methodological consideration when interpreting these outcomes is the substantial heterogeneity in how MeVOs are defined across studies. The first key dimension of variation concerns the inclusion of dominant M2 occlusions. Dominant M2 occlusions—arguably the most clinically significant MeVO subtype—wereexplicitly excluded from the DISTAL trial, while ESCAPE-MeVO and ORIENTAL-MeVO included them. DISCOUNT excluded proximal M2 occlusions (defined as M2 segment below the mid-height of the insula), which substantially overlaps with but is not anatomically identical to the dominant M2 exclusion used in DISTAL. A second dimension relates to the distal extent of the definition: some studies excluded M2 entirely (e.g., DUSK and (8)), whereas the four RCTs included M2-M4 segments, with varying restrictions on the proximal M2 portion (DISTAL excluded dominant M2; DISCOUNT excluded proximal M2; ORIENTAL-MeVO excluded dominant M2 and required diameter ≤2.0 mm for codominant/nondominant M2). Third, studies differed in their coverage of the anterior and posterior circulation, with certain studies restricting their populations to specificterritories—for example, (68) limited inclusion to M3 and ACA; (69) to ACA only; and (56) to MCA only.

These differences carry direct implications for outcome comparisons. More distal occlusions are generally associated with smaller infarct volumes and better natural history, while more proximal MeVOs carry higher perforation risk but also potentially greater benefit from successful recanalization. Table 8 provides a structuredcomparison of MeVO definitions across key studies, highlighting the definitional dimensions most likely to influence outcome comparisons.

A detailed, study-by-study overview of efficacy and safety outcomes is provided in Supplementary Table S8. A concise summary of evidence levels is presented in Table 3.

#### 3.2.1 Clinical functional outcomes (90-day mRS)

A concise summary of evidence levels for all included studies is presented in Table 3, and complete study-by-study outcome data are provided in Supplementary Table S8. The Tier I evidence from the four pivotal RCTs yielded mixed results, with two trials showing neutral outcomes, one demonstrating significant benefit of EVT, and one terminated early due to a harm signal. Specifically, the DISTAL and ESCAPE-MeVO trials found no statistically significant difference in the rate of 90-day functional independence (mRS 0–2) between the EVT and BMT groups. The ORIENTAL-MeVO trial demonstrated a statistically significant benefit of EVT over BMT for 90-day functional independence (58.6% vs. 46.6%; aRR 1.24, 95% CI 1.07–1.44, *p* = 0.004). The DISCOUNT trial was terminated prematurely after a pre-specified interim analysis suggested worse functional outcomes, higher mortality, and increased symptomatic intracranial hemorrhage in the EVT group; complete peer-reviewed outcome data are not yet available.

Formal assessment of heterogeneity across the four RCTs confirmed that meta-analysis would be inappropriate. Substantial between-study variability was evident in the primary outcome results (I^2^ > 75%, τ^2^ > 0.1), reflecting the discordance between the neutral findings of ESCAPE-MeVO and DISTAL, the positive result of ORIENTAL-MeVO, and the early termination of DISCOUNT due to harm. This statistical heterogeneity, combined with the clinical heterogeneity documented in Table 8, 4, precluded meaningful pooling of the RCT data and supported the narrative synthesis approach adopted throughout this review.

A conceptual subgroup framework exploring the association between key patient and occlusion characteristics and 90-day functional outcomes is presented in Figure 3.

**Figure 3:**
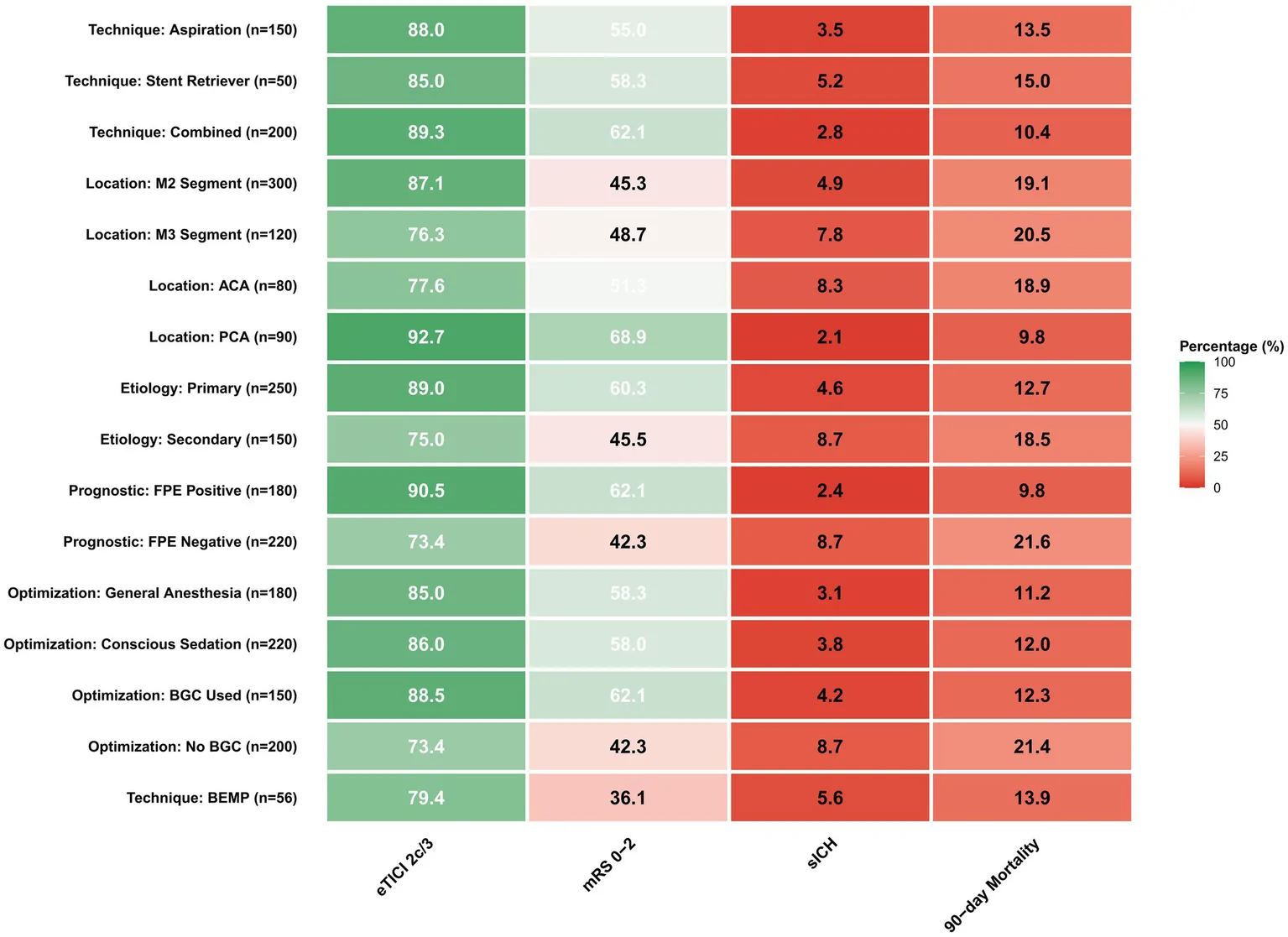
Conceptual framework: subgroup analysis for MeVO thrombectomy. Data represent author estimates derived from narrative synthesis of included studies. They should not be interpreted as pooled effect estimates. Individual study data are available in Supplementary Table S8. eTICI 2c/3, successful recanalization; mRS 0–2, functional independence; sICH, symptomatic intracranial hemorrhage; FPE, first−pass effect; BGC, balloon guide catheter; BEMP, blind exchange with mini−pinning; PCA, posterior cerebral artery; ACA, anterior cerebral artery.

In contrast, the majority of Tier II and III observational studies have generally reported higher rates of functional independence following MeVO thrombectomy. Rates commonly range from 60 to 80%, with some registries, such as the REVISAR study for the APERIO device, reporting rates as high as 79% (18, 19). This discrepancy may suggest a potential benefit of EVT in specific real-world patient subgroups. These subgroups include patients with higher baseline stroke severity, more favorable imaging profiles, or those treated with contemporary techniques. However, this effect appears diluted in all-comers RCT designs.

#### 3.2.2 Revascularization outcomes

Technical success, measured by successful reperfusion (eTICI ≥2b), is frequently achieved. In the completed RCTs, the rates in the EVT arms were 71.7% (DISTAL), 75.1% (ESCAPE-MeVO), and 74.4% (ORIENTAL-MeVO). The DISCOUNT trial was terminated early and complete reperfusion data have not been published. Observational series often report even higher recanalization rates, frequently exceeding 85–90%, reflecting both operator experience and the use of advanced, dedicated low-profile devices (7, 9, 20, 21).

The First-Pass Effect (FPE) is a critical technical benchmark strongly associated with excellent clinical outcomes. Reported FPE rates show considerable variability across studies, spanning from approximately 24 to 70% (22). This wide range likely reflects heterogeneity in occlusion characteristics (clot consistency, location), device selection strategy (aspiration-first vs. stent retriever-first), and operator technique.

#### 3.2.3 Safety outcomes and complications

Safety profile analysis presents a nuanced picture essential for risk–benefit assessment. Technically, EVT for MeVOs is inherently more demanding. The intervention necessitates navigation through longer, more tortuous, and smaller-caliber vascular pathways to reach the target occlusion, which increases the risks of access failure, vessel dissection, and iatrogenic perforation (23). This anatomical complexity directly contributes to the heightened safety considerations in MeVO thrombectomy.

1. The most consistently observed safety concern was symptomatic intracranial hemorrhage. All four pivotal RCTs reported a higher incidence of sICH in the EVT groups compared to the BMT control groups (ESCAPE-MeVO: 5.4% vs. 2.2%; DISTAL: 5.9% vs. 2.6%; ORIENTAL-MeVO: 4.7% vs. 2.2%) (6–9). The DISCOUNT trial was terminated early after an interim analysis that indicated an increased sICH rate in the EVT arm, though complete peer-reviewed data are not yet available. Rates in observational studies generally fall within this 0–8% range, though direct comparisons are limited by heterogeneous definitions and reporting standards (21, 22). The consistently higher sICH rate with EVT across all completed trials represents one of the most robust safety signals in the MeVO literature and should be weighed carefully in treatment decisions. The early termination of the DISCOUNT trial due to a harm signal that included increased sICH further reinforces this concern.

A second major complication was vessel perforation, a more frequent and serious event specific to MeVO thrombectomy. Multiple studies report perforation rates between 2 and 5%, approximately double the rate observed in LVO thrombectomy (24, 25). The smaller vessel caliber, thinner arterial walls, and greater navigational complexity of the distal vasculature contribute to this elevated risk. Perforation is strongly correlated with higher rates of sICH and is a devastating event: mortality in patients with intraprocedural perforation reaches approximately 50%, and active bleeding persisting beyond 20 min further worsens survival (26). These findings underscore the critical importance of device selection, operator experience, and meticulous technique in minimizing this complication.

Other complications, including embolization to new territory and arterial dissection, occurred less frequently, typically between 1 and 5% (15, 27). Subarachnoid hemorrhage, both symptomatic and asymptomatic, is increasingly recognized as a distinct complication of MeVO thrombectomy, with reported rates varying widely depending on detection methodology and classification criteria (15, 28). Transient catheter-induced vasospasm was also noted, complicating the procedure and affecting outcomes (29, 30).

Mortality results were mixed across the evidence base. Among the completed RCTs, ESCAPE-MeVO reported higher mortality in the EVT group (13.3% vs. 8.4%) (6), while DISTAL (15.5% vs. 14.0%) (7) and ORIENTAL-MeVO (11.1% vs. 10.2%) (8) showed similar rates between groups. The DISCOUNT trial’s interim analysis also indicated higher mortality in the EVT arm, although final adjudicated data are pending (9). Observational data also show variability, necessitating cautious interpretation. A key limitation in interpreting mortality data is that successful recanalization does not universally equate to survival. This is particularly true for patients with large baseline infarcts, advanced age, or significant comorbidities (31, 32).

Several observational studies specifically examined the impact of anesthesia modality on outcomes following MeVO thrombectomy. Mohammaden et al. conducted a propensity score-matched analysis comparing general anesthesia (GA) with non-GA (conscious sedation or local anesthesia) in 122 patients with distal MeVO and found no significant difference in 90-day functional independence (GA 45.5% vs. Non-GA 43.6%; OR 1.14, 95% CI 0.44–2.96, *p* = 0.79), although GA was associated with a numerically higher rate of successful reperfusion (eTICI ≥2b: 90.2% vs. 78.7%, *p* = 0.12) and a non-significantly higher sICH rate (15.5% vs. 4.5%, *p* = 0.09) (33). Similarly, Mehta et al. reported that conscious sedation was associated with higher odds of early neurological improvement (aOR 2.59, *p* < 0.05) and successful reperfusion (aOR 2.33, *p* < 0.05) compared with GA in a cohort of 279 DMVO patients, with no significant difference in 90-day good outcome (mRS 0–3: CS 41.7% vs. GA 34.4%, *p* = 0.12) (34). Valente et al. examined 94 patients with isolated M2 occlusion and minor stroke (NIHSS ≤5) and found no difference in 90-day excellent outcome (mRS 0–1) between GA and non-GA (74.5% vs. 72.3%; aOR 1.12, 95% CI 0.45–2.79, *p* = 0.815) (35). Meyer et al. (12) analyzed 233 patients with primary isolated PCA or ACA DMVO and reported no significant difference in complete reperfusion (mTICI 3) between local anesthesia ± conscious sedation and GA (73.9% vs. 71.9%, *p* = 0.729), although GA was associated with superior complete reperfusion in the ACA subgroup (aOR 3.07, 95% CI 1.24–7.57, *p* = 0.015). Berberich et al. (36) studied 376 patients with isolated PCA occlusion and found that GA was associated with higher successful reperfusion (eTICI ≥2b: 83.6% vs. 73.6%; aOR 1.70, *p* = 0.005) but no difference in functional independence (mRS 0–2: GA 47.7% vs. non-GA 50.0%; IPTW OR 0.84, 95% CI 0.50–1.39, *p* = 0.49). Collectively, these data suggest that while GA may facilitate higher rates of successful reperfusion, particularly in technically challenging occlusions, this does not consistently translate into improved functional outcomes. The choice of anesthesia modality should therefore be individualized based on patient characteristics, occlusion location, and institutional expertise (12, 33, 35–37).

### 3.3 Interventional devices and techniques

#### 3.3.1 Technical principles and the evolving device arsenal

The successful execution of MeVO thrombectomy requires a precision strategy tailored to the unique anatomical constraints of the distal vasculature—smaller diameter, thinner walls, and greater tortuosity than LVO targets (23, 38). The key design objectives guiding next-generation device development to meet these challenges are summarized in Table 6.

**Table 6:** Evolution of devices.

Direction of evolution	Design objectives and pain points	Specific technical concepts
Smaller outer diameter/improved deliverability	MeVO involves smaller vascular diameters and more tortuous pathways, for which conventional devices designed for LVO are inadequate.	The stents and catheters feature elongated lengths, reduced outer diameters, and enhanced flexibility (e.g., through mesh-like structures), with surfaces coated with hydrophilic coating to minimize friction during advancement.
Material mechanics balance	Medium-sized blood vessels feature thinner walls and more tortuous paths, making them prone to complications such as perforation and hemorrhage.	• Improved material flexibility or tip design, such as a self-expanding funnel structure; • Intermediate catheters must possess both flexibility and strong support.
Enhance thrombus capture.	The thrombus is more friable, with a tendency to fragment, complicating retrieval.	Thrombectomy devices incorporate localized flow restriction to minimize thrombus fragmentation, while aspiration systems utilize augmented suction force to improve capture rates.
Bifurcation-friendly design	The altered hemodynamics due to the anatomical complexity at vascular bifurcations can significantly impact the efficacy of thrombectomy procedures.	• Enhance precise vascular navigation capability of the thrombectomy system. • Improve the accuracy of imaging equipment in identifying vascular morphology and variations.

MeVO, medium vessel occlusion; LVO, large vessel occlusion.

Note: This table represents the authors’ conceptual synthesis of the key design objectives and technological directions required to address the unique challenges of MeVO thrombectomy. These design concepts are not intended to represent characteristics of any specific commercially available device. They should be regarded as a hypothesis-generating framework to guide future device development and evaluation.

**Table 7:** A hypothetical framework for interpreting discrepancies between randomized controlled trial results and real-world evidence for MeVO thrombectomy.

Dimension	RCT characteristics	Observational study characteristics	Key differences
Inclusion and exclusion criteria	Broad eligibility may dilute treatment effect; physician beliefs may lead to selective enrollment (7).	Clinician preference for patients with more severe symptoms and significant salvageable tissue	“Protocol-driven” vs. “Clinically driven”
Time window	Rigid time windows (≤12–24 h); post-awakening strokes generally excluded	Flexible extension to 24 h; comprehensive clinical-imaging assessment for all patients with salvageable tissue	“Clock time” vs. “Tissue time”
Imaging standards	Mandatory advanced perfusion imaging with strict mismatch thresholds; independent core lab adjudication	Rapid CTA/NCCT-based “clinical-vascular mismatch” decision-making; CTP as adjunct only	“Perfect imaging” vs. “Sufficient imaging”
Anesthesia management	Preference for GA to ensure immobility; potential for hemodynamic instability and preparation delays	Primary choice of conscious sedation/local anesthesia to minimize door-to-puncture time and maintain hemodynamic stability	“Process control” vs. “Physiological optimization”
Procedural delays	Consent, central adjudication, and imaging processing introduce systematic delays (6, 7).	Optimized “code stroke” workflows minimize door-to-puncture time	“Research pathways” vs. “Clinical pathways”
Operator experience and device evolution	Early-phase device-vessel mismatch; 80% SR use despite evolving technology; absence of individual credentialing (6, 66).	Accumulated operator expertise; dedicated low-profile devices; refined technique combinations (BEMP, SAVE)	“Technological limitations” vs. “Technological evolution”
Rescue strategy flexibility	Standardized protocols restrict rescue strategies; first-line device often mandated (6).	Standardized protocols restrict rescue strategies; first-line device often mandated (2)	“Rigid protocols” vs. “Individualized rescue”
High BMT efficacy and sample size	High control group success (mRS 0–2: 46.6–58.8%); very large sample sizes required to detect modest additive benefit	Observational studies may capture effect sizes in selected subgroups that are difficult to replicate in all-comers trial designs	“Statistical power dilution” vs. “Enriched subgroup signals”

RCT, randomized controlled trial; EVT, endovascular thrombectomy; M2, M2 segment of the middle cerebral artery; CTP, computed tomography perfusion; MRP, magnetic resonance perfusion; ASPECTS, Alberta Stroke Program Early CT Score; CTA, computed tomography angiography; NCCT, non-contrast computed tomography; GA, general anesthesia; SR, stent retriever.

This framework represents the authors’ hypothetical interpretation of design-level differences between RCTs and observational studies. These proposed mechanisms are based on group-level comparisons and have not been empirically validated as causal explanations for the observed efficacy gap. Each dimension should be interpreted with appropriate epistemological caution.

The DISCOUNT trial, which was terminated early due to a harm signal, further illustrates several of these dimensions: its narrow 6-h primary time window and exclusion of proximal M2 may have restricted enrollment of the population most likely to benefit from EVT, and the high expected rate of good outcomes with BMT alone limited the power to detect a modest additive benefit of thrombectomy.

**Table 8:** Heterogeneity of MeVO definitions across key included studies.

Study (author, year)	MeVO definition summary	Dominant M2 included?	Non-dominant M2 included?	M3/M4 Included?	ACA (A2-A4) included?	PCA (P2-P4) included?	Key definition specifics
Randomized controlled trials							
ESCAPE-MeVO (Goyal, 2025) (6)	M2, M3, A2, A3, P2, P3; excludes A1/P1	Yes	Yes	Yes (M3)	Yes (A2, A3)	Yes (P2, P3)	Proximal M2: ≤1 cm from bifurcation; distal M2: to circular sulcus; M3: opercular
ORIENTAL-MeVO (Hu, 2026) (8)	Codominant/nondominant M2 or M3; A1-A3; P1-P3	Yes (codominant)	Yes	Yes (M3)	Yes (A1-A3)	Yes (P1-P3)	Dominant M2 excluded; M2 diameter ≤2.0 mm
DISTAL (Psychogios, 2025) (7)	Nondominant/codominant M2-M4; A1-A3; P1-P3	No (excluded)	Yes	Yes	Yes	Yes	Only RCT to explicitly exclude dominant M2
DISCOUNT (Clarençon, 2024) (9)	Distal M2 (above mid-height of insula), M3; A1-A3; P1-P3	No (proximal M2 excluded by anatomical landmark)	Yes (distal M2 only)	Yes (M3)	Yes (A1-A3)	Yes (P1-P3)	Only RCT to exclude proximal M2 using an anatomical landmark (mid-height of insula) rather than functional dominance; maximum 3 passes
Key observational studies with distinct definitions							
TOPMOST (Meyer, 2021) (12)	Isolated PCA P2/P3 only	N/A	N/A	N/A	N/A	Yes (P2, P3)	Excludes anterior circulation MeVO entirely
TOPMOST (Meyer, 2023) (12)	Isolated ACA A2-A4 only	N/A	N/A	N/A	Yes (A2-A4)	N/A	Excludes A1, posterior circulation MeVO
DUSK (Mohammaden, 2024) (34)	M3/M4; A2/A3; P1/P2	N/A	N/A	Yes	Yes	Yes	Excludes all M2; PCA-P3 excluded
Hu, 2026 (8)	M3/M4; A1-A3; P1-P3	N/A	N/A	Yes	Yes	Yes	Excludes M2 entirely; diameter 0.75–2.0 mm
Saber, 2022 (67)	M3 MCA; any ACA segment	N/A	N/A	Yes (M3 only)	Yes (A1-A3)	No	Narrowest definition; only anterior DMVO
MAD-MT (Salim 2025) (51)	MCA M2, M3, M4 only	Yes	Yes	Yes	No	No	Excludes ACA/PCA MeVO entirely
Zhang, 2026 (68)	ACA A1, A2, A3 only	N/A	N/A	N/A	Yes (A1-A3)	N/A	Only ACA territory MeVO
All other observational studies	Variable definitions across M2-M4, A2-A4, P2-P4	Variable	Variable	Variable	Variable	Variable	See Table 5 for individual study definitions

RCT, randomized controlled trial; M2, M3, M4, middle cerebral artery segments; ACA, anterior cerebral artery; PCA, posterior cerebral artery; N/A, not applicable.

This table illustrates the substantial heterogeneity in MeVO definitions across included studies. The selected studies represent key examples of distinct definitional approaches. Detailed MeVO definitions for all 38 included observational studies are provided in Table 5. N/A denotes that a definitional dimension is not applicable to the study’s prespecified occlusion sites.

This focused engineering effort has yielded two core technological platforms. Dedicated low-profile stent retrievers (SRs) such as the Trevo NXT (3 mm/4 mm variants), Solitaire X, APERIO Hybrid 17, Tigertriever 13, and Catch Mini are designed with reduced strut dimensions and specialized cell structures, enabling safe navigation in arteries as small as 1.5 mm (16, 39, 40). Distal aspiration catheters, including the AXS Catalyst 5, 3MAX, and 4MAX, offer an alternative or complementary approach, with improved deliverability through slender profiles, tapered designs, and hydrophilic coatings (12).

A pragmatic, phenotype-driven approach to device category selection is suggested by current evidence. Medium-sized aspiration catheters may be favored for very distal occlusions, such as M3 or M4 segments. They are also preferred in cases of highly tortuous anatomy. This preference is due to their superior anatomical compatibility and lower risk of endothelial injury. Conversely, dedicated low-profile stent retrievers may be preferable for proximal M2 occlusions. This is particularly true when the thrombus burden is larger or the clot is more organized. These devices leverage their defined radial force and superior grip to achieve effective retrieval. This principle is termed thrombus-characteristic suitability (16, 41, 42). Detailed efficacy and safety metrics for specific device categories and advanced technique combinations (e.g., SAVE, Double SR, QUATTRO, BEMP) are summarized in Supplementary Table S7.

#### 3.3.2 Mainstream technical combinations

Beyond the choice between a primary SR or aspiration strategy, several advanced technique combinations have been adapted or specifically developed for the complexities of MeVOs. Detailed efficacy and safety metrics for each technique are provided in Supplementary Table S7.

Beyond the choice between a primary SR or aspiration strategy, several advanced technique combinations have been adapted or specifically developed for the complexities of MeVOs. Direct aspiration (ADAPT) involves navigating a large-bore distal aspiration catheter to the clot face and applying suction. The Solumbra/SPAN technique combines a stent retriever with an intermediate catheter that is advanced to provide proximal aspiration during retrieval. The SAVE technique deploys a stent retriever directly through a large-bore aspiration catheter; after deployment, the stent is pulled back into the catheter tip, and both are withdrawn under continuous aspiration, effectively “sandwiching” the clot (43, 44). The double stent retriever technique deploys two low-profile stent retrievers in a “Y” or “T” configuration within a bifurcation occlusion, increasing the surface area for clot engagement and retrieval force (45). The QUATTRO technique utilizes a sophisticated multi-device system with a long sheath, a distal access catheter, a microcatheter, and a microwire to create a stable “four-axis” platform for navigating complex anatomy and executing combined techniques (46). The Blind Exchange with Mini-Pinning (BEMP) technique combines a mini stent retriever with a slender distal aspiration catheter and has been associated with higher first-pass recanalization rates and lower sICH rates in MeVO compared to mini SR alone (22). Detailed efficacy and safety metrics for each technique are provided in Supplementary Table S7.

#### 3.3.3 A decision-making framework: from imaging phenotype to intervention

A precision medicine approach mandates a structured, pre-procedural analysis of multimodal imaging to guide device and technique selection (38). This operational framework can be broken down into a logical, sequential process.

The first step is a rapid diagnostic pathway. For any patient with suspected acute stroke and a significant or disabling deficit, activation of a standardized “one-stop” imaging protocol is critical. The recommended pathway is illustrated in Supplementary Figure S1, with technical specifications detailed in Supplementary Table S9. This pathway begins with non-contrast CT to exclude intracranial hemorrhage, followed by CT angiography to identify and localize the occlusion, and finally CT perfusion to physiologically characterize the tissue at risk. CT perfusion is indispensable for quantifying the ischemic core and the salvageable penumbra, enabling the definition of a “Target Mismatch” profile (e.g., core volume <30–50 mL, mismatch ratio ≥1.2, mismatch volume ≥10 mL)—the key imaging criterion for identifying patients most likely to benefit from thrombectomy (47, 48).

The imaging work-up should then be analyzed to delineate four key, strategy-informing phenotypes: the exact occluded segment and the functional eloquence of the downstream brain territory (site and territory); the tortuosity of the parent vessel and the estimated caliber of the target vessel (anatomy and access); clot length and density on CTA/NCCT (thrombus burden and characteristics); and suspicion for underlying intracranial atherosclerotic disease versus a pure embolic occlusion (underlying pathology). A detailed guide for mapping these phenotypes to specific device choices is provided in Supplementary Table S10.

The synthesis of this analysis then guides the selection of a streamlined therapeutic approach. For example, a proximal M2 occlusion with large clot burden favors a combined technique (SAVE, BEMP) or a stent retriever; a distal M3/M4 occlusion with tortuous access favors a low-profile stent retriever or a small-bore aspiration catheter; and suspected underlying ICAD mandates preparation for potential rescue angioplasty with or without stenting. The clinical rationale and levels of evidence supporting these phenotype-to-technique correlations are synthesized in Supplementary Table S11, and a practical, evidence-informed summary ofrecommended techniques for specific anatomical and clot scenarios is provided in Supplementary Table S12.Quality of Evidence Assessment (GRADE).

Applying the GRADE framework to the body of evidence yields the following certainty ratings for core clinical questions in MeVO thrombectomy. A detailed GRADE Summary of Findings table, including domain-level justifications for each rating, is provided in Supplementary Table S17.

Applying the GRADE framework to the body of evidence yielded the following certainty ratings for core clinical questions in MeVO thrombectomy. For the comparative effectiveness of EVT versus BMT, the certainty of evidence was rated as low. Although four RCTs provide high-level evidence, the majority of included studies are observational with inherent risk of residual confounding (risk of bias). Furthermore, the results of the four pivotal RCTs are inconsistent, with two neutral (ESCAPE-MeVO, DISTAL), one positive (ORIENTAL-MeVO), and one terminated early due to a harm signal (DISCOUNT) (inconsistency). The early termination of DISCOUNT and the unavailability of its complete peer-reviewed outcome data further increase the risk of bias. These limitations warranted downgrading by two levels. The certainty of evidence for the efficacy and safety of new dedicated devices and techniques was rated as very low. The evidence originates primarily from single-arm studies or technical comparisons without medical control arms, which are subject to selection bias and confounding by indication (risk of bias downgraded by two levels). Small sample sizes and low event rates further limit the precision of effect estimates (imprecision). Regarding the role of advanced imaging for diagnosis and eligibility selection, the certainty of evidence was rated as low. Robust and consistent observational data demonstrate an association between favorable imaging profiles and improved outcomes. However, key perfusion parameters—such as the Hypoperfusion Intensity Ratio (HIR)—were originally developed and validated in LVO populations, and their application to MeVO is therefore extrapolative, constituting indirectness that warranted downgrading by one level. For the impact of anesthesia management on outcomes, the certainty of evidence was also rated as low. Evidence originates from non-randomized comparisons subject to selection bias and residual confounding (risk of bias). Moreover, results are conflicting, with some studies suggesting potential harm with general anesthesia while others show no difference compared with conscious sedation (inconsistency). These limitations warranted downgrading by two levels. A detailed GRADE Summary of Findings table, including domain-level justifications for each rating, is provided in Supplementary Table S17.

## 4 Discussion

### 4.1 A hypothetical framework for interpreting neutral RCT outcomes

The mixed results of the four pivotal MeVO trials—ESCAPE-MeVO (neutral), DISTAL (neutral), ORIENTAL-MeVO (positive), and DISCOUNT (terminated early due to harm)—necessitate a sophisticated, multidimensional interpretation rather than a simplistic dismissal or endorsement of EVT. To deconstruct the potential factors contributing to these results, we propose a hypothetical framework comprising eight dimensions (Table 7). This framework aims to reconcile the divergent trial findings with positive real-world experience. Each dimension represents a speculative, author-proposed explanation. It should be regarded as hypothesis-generating rather than empirically validated.

The first dimension concerns inclusion and selection criteria. Pragmatic trial designs with broad eligibility criteria may dilute the treatment effect. This is because they enroll patients with inherently favorable natural history. These patients establish a high “ceiling” for recovery with best medical therapy alone. In contrast, real-world practice selectively enrolls patients with more severe deficits and clear salvageable tissue, enriching for those most likely to benefit from EVT. This dimension also encompasses the “patient spectrum” issue. Randomization may overlook clear beneficiaries, such as younger patients and those with dominant-hemisphere M2 occlusions. At the same time, it may include non-intervention candidates. This inadvertently dilutes the observable treatment effect. The DISCOUNT trial illustrates this dimension: its exclusion of proximal M2 occlusions (using the mid-height of the insula as an anatomical landmark) may have further restricted enrollment of patients with the greatest potential for benefit from EVT.

A second key dimension is the time window. RCTs enforce rigid time windows (≤12–24 h) that automatically exclude patients beyond these boundaries. Real-world practice extends to 24 h, using comprehensive clinical and imaging assessment to identify all patients with salvageable tissue, irrespective of chronological time. This shift from “clock time” to “tissue time” captures patients with favorable collateral profiles who may still benefit from late intervention. DISCOUNT employed a narrower primary time window (≤6 h, extendable to 24 h only if MRI FLAIR was negative), which may have limited enrollment of patients presenting beyond 6 h who nonetheless had salvageable tissue.

Third, imaging standards differ substantially between trial and real-world settings. Trials mandate advanced perfusion imaging with strict mismatch thresholds (e.g., core <50 mL, mismatch ratio ≥1.2), potentially excluding patients who could benefit but do not meet these criteria. Real-world practice often relies on rapid CTA/NCCT-based “clinical-vascular mismatch” decision-making, prioritizing speed and clinical relevance over physiological perfection. This difference in imaging strategy may disproportionately impact MeVO, where perfusion abnormalities are smaller and more susceptible to artifact.

Anesthesia management represents a fourth dimension. Many trial protocols favor general anesthesia for procedural control, introducing potential hemodynamic risks (hypotension, prolonged preparation time) that may secondarily injure ischemic tissue. Real-world practice increasingly prefers conscious sedation to maintain hemodynamic stability, enable intraoperative neurological monitoring, and minimize door-to-puncture times.

Fifth, procedural delays inherent to trial protocols—including informed consent processes, central adjudication, and advanced imaging processing—introduce systematic delays that can negate potential treatment benefits in time-sensitive MeVO intervention (7, 20, 49). Optimized real-world “code stroke” workflows minimize time to reperfusion, preserving the biological benefit of “time is brain.”

The sixth dimension relates to operator experience and device evolution. Trials conducted during early phases of MeVO-specific device development and operator learning curves may not reflect current achievable outcomes. Contemporary practice benefits from several key advances. These include dedicated low-profile stent retrievers and aspiration catheters, refined technique combinations (e.g., BEMP, SAVE), and accumulated operator expertise. As a result, higher technical success and potentially better clinical outcomes have been achieved.

Seventh, rescue strategy flexibility differs markedly. Standardized trial protocols may restrict rescue strategies. This is done to maintain internal validity. In contrast, real-world practice employs flexible, individualized rescue interventions. These include rescue stenting, intra-arterial thrombolysis, and multi-device combinations. Such interventions can improve final recanalization rates in refractory occlusions.

Finally, high BMT efficacy and sample size limitations constitute an eighth dimension. Modern BMT, including high rates of IVT, leads to good outcomes in a substantial proportion of MeVO patients. The control group success rates in recent trials (mRS 0–2: 46.6–58.8%) set a formidable bar for EVT to clear. Demonstrating a statistically significant additive benefit requires very large sample sizes. Such sample sizes may be infeasible within individual trials. This is particularly true given the relatively modest absolute benefit margin expected in this population. The DISCOUNT trial, which was terminated early after an interim analysis suggested harm, exemplifies how a high expected rate of good outcomes with BMT alone may leave insufficient statistical room to detect a modest benefit—or even expose excess harm—when EVT is applied to an insufficiently selected population.

This framework suggests a key insight. Neutral RCT results may reflect dilution from broad inclusion criteria, evolving technology, and protocol-driven constraints. They do not necessarily negate EVT’s potential value in appropriately selected patients. Each dimension is further elaborated in Table 7.

To provide empirical context for this framework, Table 4 systematically compares the baseline characteristics of the four pivotal RCTs with representative observational data. It highlights key differences in stroke severity, imaging selection, and occlusion definitions. These differences may contribute to the observed efficacy gap.

A critical design feature of the DISTAL trial that further illustrates this framework is its explicit exclusion of dominant M2 segment occlusions. Dominant M2 occlusions represent a clinically important MeVO subgroup. They typically supply larger territories of eloquent cortex compared to non-dominant or more distal occlusions. As a result, they are associated with more severe neurological deficits and larger infarct volumes. Consequently, patients with dominant M2 occlusions may have the greatest potential to benefit from successful reperfusion—a benefit that was systematically excluded from the DISTAL trial by design. This exclusion likely contributed to the neutral overall result. It enriched the trial population with patients who had a more favorable natural history. These patients had a higher likelihood of good outcomes with best medical therapy alone. This directly reflects the “Inclusion and Selection Criteria” and “Patient Spectrum” dimensions of our framework. The patients who might have derived the most substantial benefit from EVT were not represented in the trial, thereby compressing the observable treatment effect. Similarly, the DISCOUNT trial excluded proximal M2 occlusions using an anatomical landmark (mid-height of the insula), which in many cases overlaps with the dominant M2 territory. This parallel exclusion may have further restricted enrollment of the subgroup with the greatest potential for benefit, and likely contributed to the early termination due to harm. This design choice has direct implications for the generalizability of the DISTAL findings: the neutral result cannot be assumed to apply to patients with dominant M2 occlusions, who constitute a substantial proportion of clinical MeVO cases. Until dedicated randomized evidence becomes available for this subgroup, clinicians managing dominant M2 occlusions must rely on observational data, which consistently suggest a benefit of EVT in appropriately selected patients with significant deficits and salvageable tissue. Based on the observational studies included in this review, the potential absolute benefit of EVT in dominant M2 occlusions is informally estimated at approximately 15–20% for 90-day functional independence, compared with best medical therapy alone. This estimate is derived from narrative synthesis of the included observational data and should be regarded as hypothesis-generating; it is not a formal pooled effect size and requires prospective validation in a dedicated randomized trial. This limitation is systematically addressed in our analysis of MeVO definition heterogeneity (Table 8). Furthermore, it is acknowledged as a key constraint on the applicability of current RCT evidence to real-world practice. This and other definitional and selection differences are systematically summarized in Table 4.

This concern regarding selective enrollment is further supported by recent empirical data. A dual-center study compared patients randomized in MeVO trials with those treated in routine clinical practice. It found that trial participants had significantly lower baseline stroke severity (median NIHSS 5 vs. 10). They also had smaller hypoperfusion volumes (27 mL vs. 48 mL). These findings suggest a systematic selection bias toward milder deficits in trials (50). These findings reinforce the need for consecutive patient recruitment and imaging-based enrichment strategies in future trials to ensure that the populations most likely to benefit from EVT are adequately represented.

This framework represents the authors’ synthesis and interpretation of study-level design differences. None of the proposed mechanisms have been empirically validated as causal explanations for the observed efficacy gap between RCTs and observational studies. This framework should be regarded as a hypothesis-generating tool to guide future research rather than an established explanatory model.

### 4.2 From framework to practice: patient selection

The “Eight Key Dimensions” analysis demonstrates the high context-dependence of MeVO thrombectomy benefit. Identifying patients most likely to derive a net clinical benefit from EVT requires a structured, multi-dimensional approach. This approach integrates clinical severity, imaging mismatch, anatomical considerations, and time from onset. For the purposes of this review, a “disabling deficit” is operationally defined as any neurological deficit that significantly impairs a patient’s occupational capacity or activities of daily living, conceptually corresponding to a modified Rankin Scale (mRS) score of ≥2–3. To translate the aforementioned conceptual framework into a structured assessment, we propose a hypothesis-generating scoring system (Supplementary Table S5). This tool is derived from author synthesis of the reviewed evidence and expert consensus, and assigns semi-quantitative weights across key domains: clinical deficit severity, imaging mismatch profile, time from onset, and anatomical/procedural factors. The threshold of NIHSS ≥5 for the +2 score was chosen as an intermediate value that synthesizes the enrollment criteria of the four pivotal RCTs: ESCAPE-MeVO (NIHSS >5 or 3–5 with disabling deficit), DISTAL (NIHSS ≥4 or disabling deficit), ORIENTAL-MeVO (NIHSS ≥6), and DISCOUNT (NIHSS ≥5). This midpoint captures the clinical severity level at which current evidence most consistently suggests a potential net benefit from EVT. Notably, the DISCOUNT trial, which required NIHSS ≥5, was terminated early due to a harm signal, underscoring that even at this threshold, patient selection must integrate additional factors such as imaging mismatch and anatomical considerations. However, this threshold has not been empirically validated as an optimal cut-off and should be interpreted with caution.

It is important to note that identical NIHSS scores may represent substantially different functional impacts depending on the occlusion site. For instance, an NIHSS of 4 from an isolated M3 branch occlusion causing mild arm weakness is categorically different from an NIHSS of 4 from a dominant proximal M2 occlusion causing aphasia. The latter represents a disabling deficit with far greater treatment urgency and potential for benefit. Therefore, the proposed scoring system should be interpreted in conjunction with the functional eloquence of the affected territory, as captured in the “Occluded Vessel Location” dimension of the tool.

Central to the imaging mismatch assessment is the identification of salvageable tissue. An emerging biomarker for this purpose is the Hypoperfusion Intensity Ratio (HIR), defined as the volumetric ratio of brain tissue with severely delayed contrast arrival (Tmax >10 s) to tissue with any perfusion delay (Tmax >6 s). A higher HIR reflects poor collateral circulation and has been shown to be a robust, independent predictor of follow-up infarct volume and functional outcomes in AIS (14, 51). Preliminary evidence specifically in MeVO populations has demonstrated that HIR is associated with follow-up infarct volume, providing an important initial validation signal for its application in distal occlusions (52). However, it should be noted that HIR was originally developed and validated in LVO populations, including the DEFUSE 2 study and related cohorts (53); its application to MeVO therefore remains extrapolative and carries greater uncertainty. Smaller perfusion defects are more susceptible to artifacts and may not be captured with the same reliability using standard perfusion thresholds (51). This limitation highlights a broader challenge in the field: the absence of MeVO-specific, prospectively validated imaging selection criteria.

In addition to perfusion-based biomarkers, traditional collateral assessment remains an important component of imaging evaluation in MeVO. This assessment uses CTA-based collateral scores. However, standardized scoring systems have not been specifically validated for distal occlusions. Furthermore, the interpretation of advanced perfusion imaging in the context of MeVO requires specific training, as perfusion abnormalities are smaller and more susceptible to artifact compared with LVO.

Recent data-driven enrichment analyses have demonstrated that the clinical benefit of successful recanalization in MeVO can be substantially amplified by applying pragmatic criteria such as age ≤80 years, baseline NIHSS ≥10, and AI-derived ischemic core volume ≤9 mL (54). These findings provide empirical support for the imaging and clinical enrichment framework proposed in this review, and suggest that future trials may achieve greater statistical power by incorporating such enrichment strategies.

It is crucial to emphasize that this tool is entirely conceptual and hypothesis-generating. Its component domains, weights, and optimal cut-off values have not been empirically derived or prospectively validated. It is intended solely to structure multidisciplinary case discussion and guide future research, not to replace clinical judgment or serve as a standalone decision aid. It must not be used for clinical decision-making in its current form. Prospective, multicenter validation is required before any potential clinical application.

### 4.3 Subgroup-driven optimization and rescue strategies

As illustrated in Figure 1, the anatomical complexity and access difficulty vary substantially across MeVO sites. The heterogeneity of MeVOs demands the abandonment of a “one-size-fits-all” technical strategy. This heterogeneity arises not only from anatomical variations but also from fundamental etiological differences. A clinically important distinction exists between primary and secondary MeVOs. Primary MeVOs are characterized by *de novo* thrombus formation in the distal vasculature. They are often related to intrinsic arterial disease or atrial fibrillation. In contrast, secondary MeVOs result from thrombus fragmentation and distal embolization of a proximal source, frequently an LVO (5, 55). Emerging data suggest secondary MeVOs may constitute 20 to 40% of cases, and patients with secondary MeVOs often present with more severe initial deficits due to the “double hit” of the initial proximal occlusion (56, 57). This etiological differentiation has direct implications for treatment planning: secondary MeVOs may respond differently to thrombectomy techniques, and their prognosis is influenced by both the original proximal occlusion and the distal embolic fragment.

Future practice must therefore pivot toward personalized approaches based on occlusion subtype and etiology. For instance, combined SR/aspiration techniques may be optimal for proximal M2 occlusions with large clot burden, while pure low-profile aspiration may suffice for distal M3/M4 branches with friable clot. In patients with secondary MeVO, careful assessment of the proximal source is essential to guide complete revascularization. Furthermore, the management of underlying intracranial atherosclerotic disease (ICAD), a common cause of refractory occlusion, requires a pre-planned strategy for rescue angioplasty and/or stenting (17, 58).

Given that procedural failure or complications remain a reality, establishing a clear framework for rescue interventions is paramount. The selection of rescue strategy should be informed by the suspected etiology of the occlusion: primary MeVO with underlying ICAD may benefit from early rescue stenting, while secondary MeVO may require proximal source control in addition to distal recanalization. Figure 4 outlines a systematic decision-making algorithm for managing EVT failure. It provides a stepwise approach based on the cause of failure: whether it is due to recalcitrant thrombus, underlying stenosis, difficult access, or a procedural complication like perforation. The algorithm integrates MeVO-specific operative thresholds, including a suggested limit of 3–4 device passes before reassessing the risk–benefit ratio, and incorporates intraoperative cone-beam CT as a decision point for detecting silent perforation. This flowchart is designed to guide operators through high-stakes scenarios, balancing the pursuit of recanalization against escalating risks.

**Figure 4:**
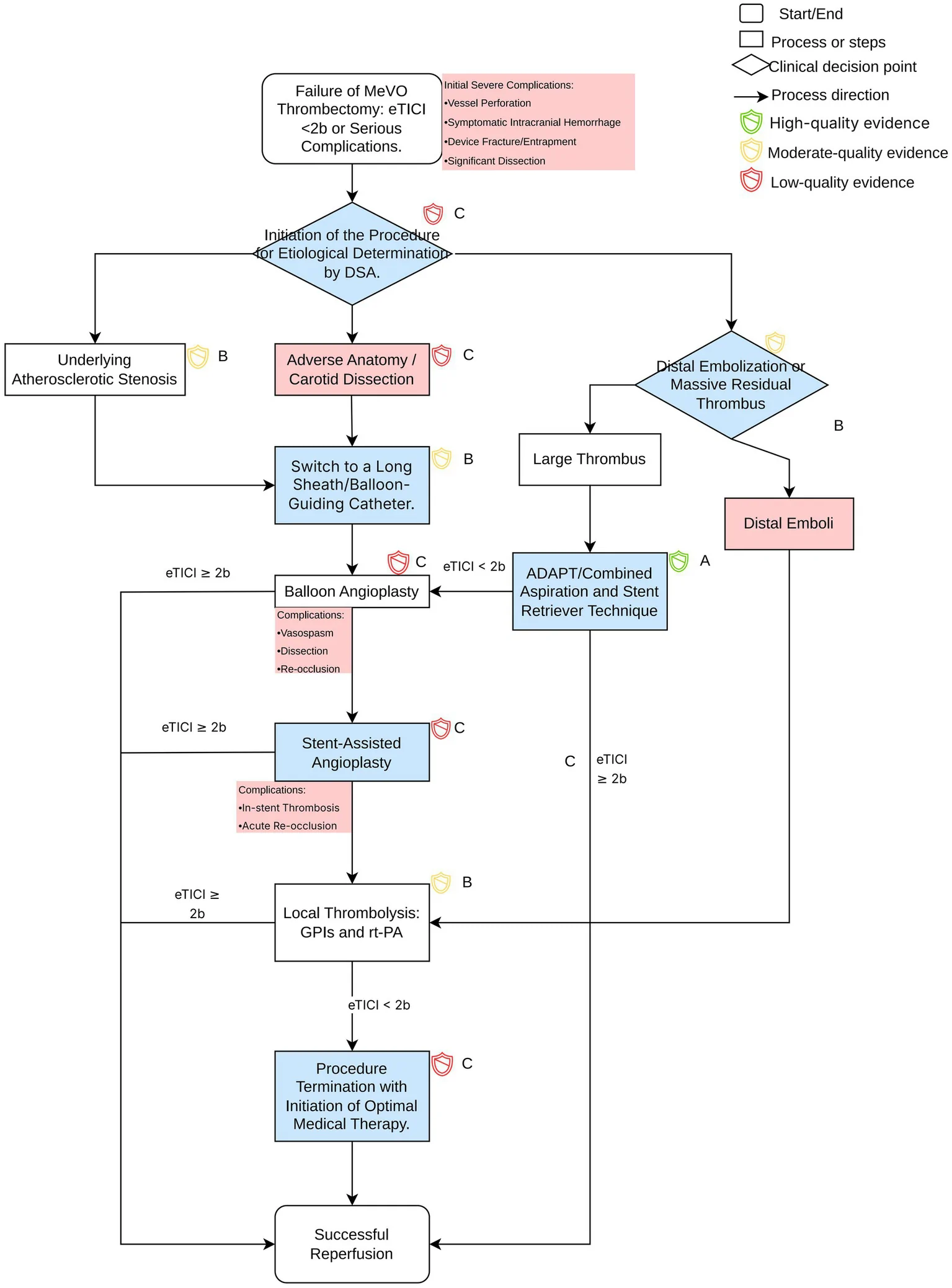
Systematic decision-making algorithm for managing failed EVT in MeVO thrombectomy. eTICI, modified thrombolysis in cerebral infarction; DSA, digital subtraction angiography; ADAPT, a direct aspiration first pass technique; GPIs, glycoprotein IIb/IIIa inhibitors; rt-PA, recombinant tissue-type plasminogen activator. The flowchart provides a stepwise approach based on the cause of failure: whether it is due to recalcitrant thrombus, underlying intracranial atherosclerotic disease (ICAD), difficult vascular access, or a procedural complication such as vessel perforation. Intraoperative cone-beam CT (CE-CBCT) should be considered as a decision point for assessing silent perforation before proceeding with additional passes. In the MeVO context, a maximum of 3–4 device passes is suggested before reassessing the risk–benefit ratio, given the higher perforation risk associated with distal, thin-walled vessels compared with LVO thrombectomy, where current guidelines suggest a pass limit of 5–6 attempts. This algorithm represents the authors’ conceptual synthesis of the reviewed evidence and expert consensus. It has not been prospectively validated and should be regarded as a hypothesisgenerating framework to guide clinical reasoning rather than a validated clinical tool.

## 5 Limitations

Several limitations of this systematic review should be acknowledged.

First, the quality of the included evidence was heterogeneous. Among the four randomized controlled trials (RCTs), two yielded neutral results (ESCAPE-MeVO, DISTAL), one showed a statistically significant benefit (ORIENTAL-MeVO), and one was terminated early due to a harm signal (DISCOUNT). The incomplete outcome data from DISCOUNT further contribute to the heterogeneity of the evidence base. The majority of included studies were observational in design, inherently subject to selection bias and confounding. Although most observational studies were assessed as moderate risk of bias by the ROBINS-I tool, residual confounding remains a concern.

Second, substantial heterogeneity was observed across the included studies in study design, patient populations (notably baseline NIHSS and imaging selection criteria), MeVO definitions, and outcome measures. A structured analysis of this heterogeneity is presented in Table 8. This analysis particularly addresses the marked variation in MeVO definitions. It examines whether dominant M2, M3/M4, or posterior circulation occlusions were included. Complete study-by-study outcome data are provided in Supplementary Table S8. These data further illustrate the heterogeneity across studies. This heterogeneity represents a fundamental barrier to evidence synthesis and precluded meta-analysis, thereby limiting our ability to derive pooled effect estimates.

Third, the literature search was conducted in three databases (PubMed/MEDLINE, Embase, and Web of Science), yielding a total of 776 records before deduplication. However, other databases such as the Cochrane Library were not searched, which may have resulted in missed relevant studies.

Fourth, only English-language publications were included, which may introduce language bias. Given the substantial volume of MeVO thrombectomy research originating from East Asian centers—particularly Japan and South Korea, where dedicated low-profile devices such as the Catch Mini, Tigertriever 13, and Trevo NXT have been extensively evaluated—the exclusion of non-English publications may disproportionately affect the generalizability of conclusions regarding specific devices and techniques. Furthermore, studies published in European non-English-language journals (e.g., German, French) may have been missed. Although efforts were made to include all relevant studies regardless of publication venue, the possibility remains that some eligible studies were inadvertently omitted due to these language and database constraints.

Fifth, publication bias cannot be ruled out, as studies with positive findings may be more likely to be published.

Sixth, this review was not prospectively registered (e.g., in PROSPERO). Although key methodological decisions—including eligibility criteria, outcome measures, and analysis plans—were prespecified by the authors before data extraction commenced, the absence of an externally time-stamped protocol means that this self-report is unverifiable. This creates the potential for unreported selective outcome reporting and constitutes a substantive methodological limitation, not merely a procedural one. It does not fully satisfy PRISMA 2020 item 24a, which requires reviewers to evaluate registration status. No internally dated protocol document is available to share as supplementary material, which represents an additional methodological constraint.

Seventh, data extraction was performed by one reviewer and verified by a second reviewer, rather than by fully independent dual extraction. The complete extracted data are transparently reported in Supplementary Table S8. This verification-based approach, while systematic, may increase the risk of transcription bias compared with independent dual extraction.

Eighth, the risk of bias assessments were conducted using the 2016 version of the ROBINS-I tool. This version does not include the refinements introduced in the draft ROBINS-I V2 (November 2025). One notable refinement absent from the 2016 version is the assessment of immortal time bias in the intervention classification and selection domains.

Ninth, the applicability of the proposed Eight Key Dimensions framework and the patient selection scoring system (Supplementary Table S5) is limited by their hypothetical and unvalidated nature. Both tools represent author-proposed conceptual syntheses that require prospective empirical validation.

Tenth, sICH definitions varied across included studies. Although the Heidelberg Bleeding Classification was specified as the reference standard, some studies employed alternative definitions, limiting comparability of safety outcomes.

Eleventh, the generalizability of findings to specific MeVO subgroups is limited. This particularly applies to dominant M2 occlusions, which were explicitly excluded from the DISTAL trial, and to proximal M2 occlusions, which were excluded from the DISCOUNT trial using an anatomical landmark (mid-height of the insula).

Twelfth, the DISCOUNT trial was included based on its published protocol and publicly reported early termination due to harm; however, complete peer-reviewed results have not yet been published. Its effect estimates remain incomplete and should be interpreted with caution.

These limitations exist despite adherence to PRISMA 2020 guidelines wherever feasible.

### 5.1 Future directions and translation to practice

Emerging technologies, including artificial intelligence (AI)-assisted MeVO detection, perfusion quantification, and procedural planning, hold promise for addressing current limitations in distal vessel recanalization. However, their clinical utility remains largely unproven: AI performance for MeVOs remains suboptimal, with lower sensitivity for M2 occlusions (approximately 40–64%) and a non-negligible false-negative rate (59, 60). Furthermore, automated perfusion analysis may fail to capture mild perfusion delays (Tmax 4–6 s) associated with small-volume ischemia (61). A fundamental safety principle must be upheld: a negative AI screen should never rule out a clinically suspected MeVO. Rigorous prospective validation is required before these technologies can be integrated into routine clinical practice. Other innovations remain at preclinical or early clinical stages. These include robotic navigation systems and advanced thrombectomy strategies, such as sonothrombolysis and dual-stent retrieval. Their efficacy and safety in MeVO-specific populations await further investigation.

The immediate research priority is the prospective validation of precision selection tools, such as the proposed scoring system, in multicenter settings. Concurrently, comprehensive health economic analyses are urgently needed. These analyses should evaluate the cost-effectiveness of MeVO thrombectomy. They must consider the high upfront costs of specialized devices and expertise against the long-term savings from reduced disability.Sustained progress hinges on parallel advancements in three key areas: (1) Refining and validating imaging-based selection criteria through collaborative research; (2) Developing and rigorously testing next-generation devices engineered specifically for the biomechanical challenges of the distal vasculature (16, 22, 39, 40); and (3) Improving the speed, accuracy, and accessibility of multimodal imaging to make precision selection feasible in all stroke-capable centers (62).

## 6 Conclusion

### 6.1 Key findings

Current randomized evidence does not demonstrate an overall functional benefit of endovascular thrombectomy for unselected MeVO stroke, while procedure-related hemorrhagic risk appears higher than with best medical therapy. EVT may be reasonable in carefully selected patients with disabling deficits, salvageable tissue, and favorable anatomy, when performed by experienced teams using distal-appropriate techniques.

### 6.2 Key recommendations

Guided by the comprehensive management pathway summarized in Figure 5, we propose the following actionable recommendations to optimize MeVO care. First, clinicians should maintain high clinical suspicion for MeVO in patients with cortical symptoms such as aphasia, neglect, or isolated motor and sensory deficits, even when NIHSS scores are modest, and expedite vascular imaging. Second, a structured imaging protocol should be implemented, utilizing a rapid “one-stop” CT, CT angiography, and CT perfusion pathway to confirm the occlusion, exclude hemorrhage, and most importantly, define the tissue pathophysiology via perfusion imaging. Third, intervention decisions should be based on a precision selection framework that combines clinical severity, including disabling deficits, with favorable imaging pathophysiology such as target mismatch, using tools like the proposed scoring system (Supplementary Table S5) to guide rather than replace clinical judgment. Fourth, in-hospital processes from door to imaging to groin puncture should be streamlined to minimize delays, given the time-critical nature of penumbralsalvage. Fifth, the thrombectomy strategy should be tailored to the specific anatomical and clot phenotype, including site, access, thrombus burden, and underlying pathology, as outlined in the decision-making framework (see Supplementary Figure S1 and Supplementary Tables S9–S11). Sixth, a predefined management algorithm for procedural failures and complications such as vessel perforation should be in place (Figure 4). Finally, clinicians should participate in registries and prospective data collection to refine selection criteria and evaluate new technologies in real-worldpractice.

**Figure 5:**
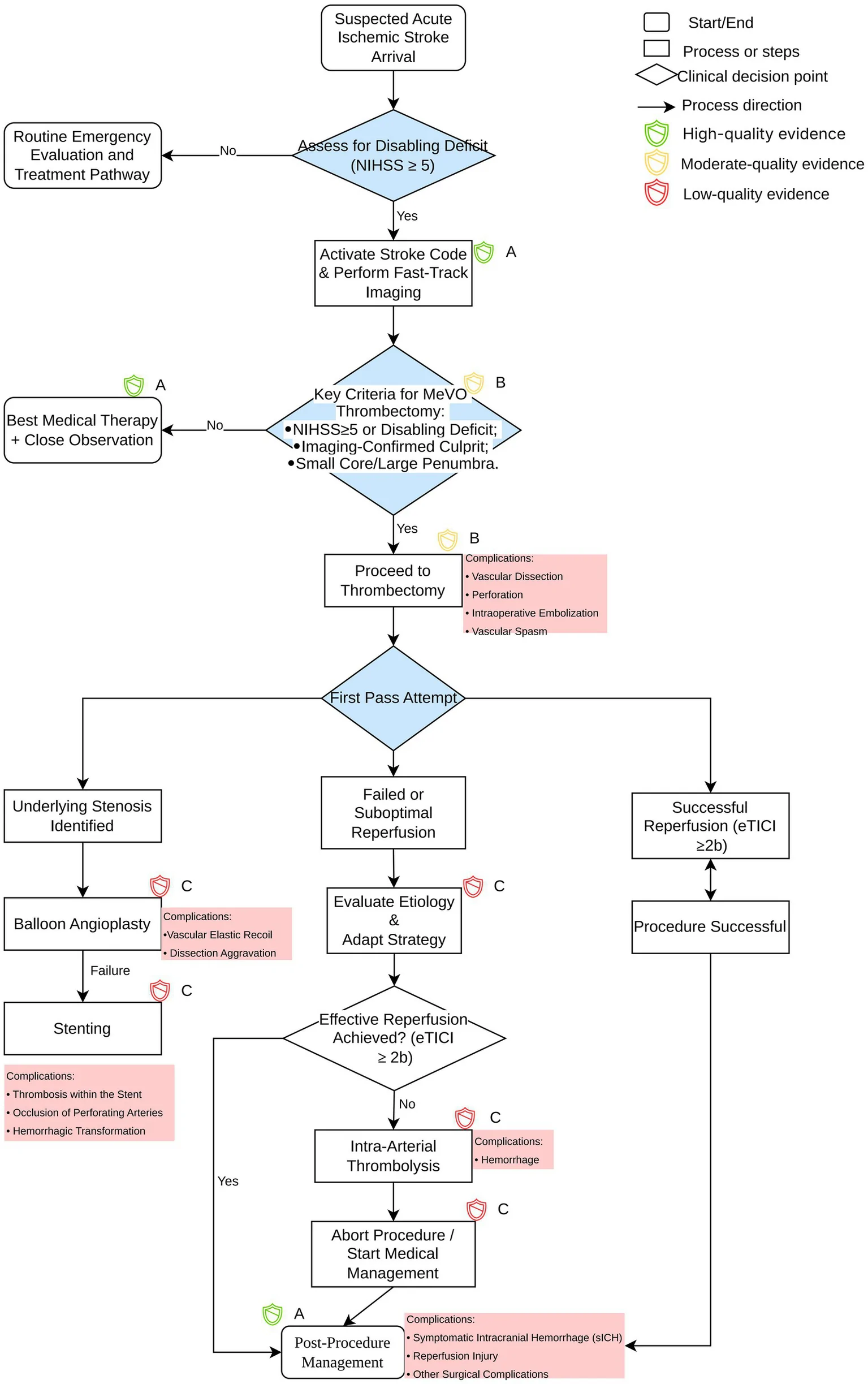
Comprehensive acute stroke management pathway. NIHSS ≥ 5, National Institutes of Health Stroke Scale score of 5 or greater (indicating a moderate to severe deficit); eTICI ≥ 2b, modified Thrombolysis in Cerebral Infarction score of 2b or greater (indicating substantial reperfusion); sICH, symptomatic intracranial hemorrhage. Note: This management pathway represents the authors’ conceptual synthesis of the reviewed evidence and established guidelines. It has not been prospectively validated and should be regarded as a framework to guide clinical reasoning rather than a validated clinical protocol.

### 6.3 Clinical significance

This systematic review synthesizes the current state of evidence and provides an integrated, practical framework for advancing MeVO stroke care. Its principal contributions are fourfold. First, it establishes the necessity for a “precision-based” paradigm, moving beyond blanket indications toward imaging-phenotype and clinically guided patient selection. Second, it offers an individualized technical roadmap, explicitly linking specific occlusion characteristics to evidence-informed device and technique choices. Third, it constructs a clear management framework for procedural challenges and complications, enhancing procedural safety. Finally, it delineates concrete future directions for research, device development, and evidence translation, culminating in a proposed RCT design checklist (Supplementary Table S13) to guide the generation of high-quality evidence for this complex population.

The evolution of MeVO thrombectomy is a testament to the ongoing refinement of stroke care. By embracing precision, personalization, and continuous evidence generation, clinicians can better navigate this complex frontier to optimize outcomes for a significant proportion of stroke patients.

## Data Availability

Publicly available datasets were analyzed in this study. All data are available from the original publications cited in the reference list.
