# Supplementary material for: Evidence, strategies, and pathways for thrombectomy in medium vessel occlusion stroke: a systematic review
Source: Front Neurol. 2026 Sep 29;17:1838008. doi: 10.3389/fneur.2026.1838008 (PMC13658856; doi:10.3389/fneur.2026.1838008)
Supplement: Supplementary file 1 [file Supplementary_file_1.docx]

**Supplementary Table S16. Domain‑level risk of bias assessment for included randomized controlled trials (RoB 2).**

Abbreviations: RoB 2, revised Cochrane Risk of Bias tool for randomized trials; D1, bias arising from the randomization process; D2, bias due to deviations from intended interventions; D3, bias due to missing outcome data; D4, bias in measurement of the outcome; D5, bias in selection of the reported result.

| Study | D1 | D2 | D3 | D4 | D5 | Overall | Weight |
| --- | --- | --- | --- | --- | --- | --- | --- |
| Goyal 2025 | Low | Low | Low | Low | Low | Low | 530 |
| Hu 2026 | Low | Low | Low | Low | Low | Low | 563 |
| Psychogios 2025 | Low | Low | Low | Low | Low | Low | 543 |
| Clarençon 2024 | Low | Low | Some concerns | Low | Low | Some concerns | N/A |

Note: Note: Four RCTs were assessed using the RoB 2 tool. Three completed RCTs (ESCAPE‑MeVO, DISTAL, ORIENTAL‑MeVO) were rated as low risk of bias across all domains. The DISCOUNT trial was rated as ‘Some concerns’ overall due to incomplete outcome data (Domain 3) resulting from early termination after a pre‑specified interim analysis suggested worse functional outcomes, higher mortality, and increased symptomatic intracranial hemorrhage in the EVT group. Complete peer‑reviewed outcome data for DISCOUNT are not yet available. The corresponding traffic light plot for all four RCTs is provided in Supplementary Figure S4.

**Supplementary Table S17. GRADE Summary of Findings (SoF) table for key clinical questions in MeVO thrombectomy.**

Abbreviations: EVT, endovascular thrombectomy; BMT, best medical therapy; mRS, modified Rankin Scale; RCT, randomized controlled trial; LVO, large vessel occlusion; MeVO, medium vessel occlusion; HIR, hypoperfusion intensity ratio; GA, general anesthesia.

| Outcome | Study Design (No. of Studies) | Risk of Bias | Inconsistency | Indirectness | Imprecision | Publication Bias | Certainty of Evidence (GRADE) |
| --- | --- | --- | --- | --- | --- | --- | --- |
| EVT vs BMT for functional independence (mRS 0–2) | 4 RCTs + 38 observational | Serious (–1)¹ | Serious (–1)² | Not serious | Not serious | Undetected | Low |
| Efficacy and safety of new devices/techniques | Observational (registries, case series) | Very serious (–2)³ | Not serious | Not serious | Serious (–1)⁴ | Undetected | Very low |
| Role of advanced imaging for diagnosis/selection | Observational (multiple cohorts) | Not serious | Not serious | Serious (–1)⁵ | Not serious | Undetected | Low |
| Impact of anesthesia on outcomes | Observational (non-randomized) | Serious (–1)⁶ | Serious (–1)⁷ | Not serious | Not serious | Undetected | Low |

notes:
¹ Downgraded for risk of bias: the majority of included studies are observational with inherent risk of residual confounding, even after statistical adjustment. Additionally, the DISCOUNT trial was terminated early after an interim analysis suggesting harm; its complete peer‑reviewed outcome data are not yet available, further increasing the risk of bias in the overall body of evidence.
² Downgraded for inconsistency: results of the four pivotal RCTs are inconsistent (ESCAPE‑MeVO and DISTAL neutral; ORIENTAL‑MeVO positive; DISCOUNT terminated early due to a harm signal).
³ Downgraded two levels for risk of bias: evidence primarily originates from single‑arm studies or technical comparisons without medical control arms, which are subject to selection bias and confounding by indication.
⁴ Downgraded for imprecision: small sample sizes and low event rates limit the precision of effect estimates.
⁵ Downgraded for indirectness: key perfusion parameters (e.g., HIR) were originally developed and validated in LVO populations; their application to MeVO is extrapolative.
⁶ Downgraded for risk of bias: non‑randomized comparisons of anesthesia modalities are subject to selection bias and residual confounding.
⁷ Downgraded for inconsistency: some studies suggest potential harm with GA, while others show no difference compared with conscious sedation.
