# Supplementary material for: Evidence, strategies, and pathways for thrombectomy in medium vessel occlusion stroke: a systematic review
Source: Front Neurol. 2026 Sep 29;17:1838008. doi: 10.3389/fneur.2026.1838008 (PMC13658856; doi:10.3389/fneur.2026.1838008)
Supplement: Supplementary file 2 [file Table_1.docx]

Supplementary Table S1. Detailed Search Strategy

| Database | Search # | Query | Results | Date |
| --- | --- | --- | --- | --- |
| PubMed | #1 | (MeVO[Title/Abstract]) OR ("Medium Vessel Occlusion"[Title/Abstract]) | 283 | 2026-05-24 |
|  | #2 | (Thrombectomy[Title/Abstract]) OR (Thrombectomies[Title/Abstract]) OR ... OR (Mechanical Clot Disruptions[Title/Abstract]) | 24,359 | 2026-05-24 |
|  | #3 (Final) | #1 AND #2 | 211 | 2026-05-24 |
| Embase | #1 | 'thrombectomy'/exp OR thrombectomy:ab,ti | 58,669 | 2026-05-24 |
|  | #2 | 'medium vessel occlusion':ab,ti OR mevo:ab,ti | 437 | 2026-05-23 |
|  | #3 | #1 AND #2 | 309 | 2026-05-23 |
| Web of Science | #1 | TS=("Thrombectomy" OR "Thrombectomies" OR ... OR "Mechanical Clot Disruptions") | 29,595 | 2026-05-23 |
|  | #2 | TS=("Medium Vessel Occlusion" OR "MeVO") | 339 | 2026-05-23 |
|  | #3 | #1 AND #2 | 65 | 2026-05-23 |

Notes: No filters or language restrictions were applied across all databases. The final combined result set was deduplicated before screening. The PubMed Search Details column has been omitted for brevity; the complete query translation is available from the corresponding author upon request.
