# Supplementary material for: Evidence, strategies, and pathways for thrombectomy in medium vessel occlusion stroke: a systematic review
Source: Front Neurol. 2026 Sep 29;17:1838008. doi: 10.3389/fneur.2026.1838008 (PMC13658856; doi:10.3389/fneur.2026.1838008)
Supplement: Supplementary file 3 [file Table_10.docx]

Supplementary Table S10. Instrument Selection for MeVO Thrombectomy Based on Imaging Characteristics

Abbreviations: M2, M2 segment of middle cerebral artery; A2, A2 segment of anterior cerebral artery; MeVO, medium vessel occlusion; LVO, large vessel occlusion; ICA, internal carotid artery; ADAPT, a direct aspiration first-pass technique; ICH, intracranial hemorrhage; EVT, endovascular therapy/mechanical thrombectomy; SR, stent retriever; CA, contact aspiration.

| Imaging Feature | Impact on Device Delivery & Manipulation (Accessibility) | Recommended Instrument Selection Strategy |
| --- | --- | --- |
| Vessel Caliber | | |
| Imaging Feature Impact on Device Delivery & Manipulation (Accessibility) Recommended Instrument Selection Strategy  Vessel Caliber Conventional MeVO: Vessel Diameter >2.0mm  (e.g., proximal M2, A2 segments) | Permits passage of smaller-profile stents and aspiration catheters. Characteristics are closer to LVO, making it more suitable for EVT ^1^. | •Stent Diameter: Small-diameter stents (e.g., Solitaire-X 3mm/4mm) and SRs (e.g., Aperio 3.5x28mm, Catch Mini 3x15mm).  • Aspiration Catheter: Distal aspiration catheters, such as the 3MAX.  • Microcatheter: Headway 17, etc.  • Support System: An intermediate catheter (e.g., Sofia 5F, Jet 7) positioned in the M1 segment for support. |
| Finer MeVO: Vessel Diameter < 2.0 mm(e.g., distal M2, M3, A3, distal P2, and P3 segments) | Compared to conventional MeVO, the smaller lumen presents greater technical challenges for thrombectomy, with a higher risk of vessel perforation and ICH. | •Stent Diameter: Use with caution or may be contraindicated. The only option, if necessary, is the smallest-profile stent (e.g., 2.0 mm diameter).  • Aspiration Catheter: First-line choice. Ultra-slim aspiration catheters (e.g., 3MAX, 4MAX) or direct aspiration via a microcatheter.  • Microcatheter: As a last resort (e.g., Trevo Pro 14, Cerebase DA), though less efficient than dedicated thrombectomy devices. |
| Vessel Tortuosity | | |
| Straight M1 segment, direct access post-bifurcation | Coronary artery experience can be referenced, though further research is needed ^2^. | Stent/Catheter: Most devices designed for MeVO can navigate successfully. Dual-lumen or angled microcatheters are important tools for accessing challenging branch vessels ^3^.  Combined technique (SR + CA) may achieve faster reperfusion in certain scenarios, such as after initial failure ^2^. |
| Acute-angled bifurcation at M1 terminus, tortuous path to target vessel | Tortuous vasculature is associated with lower final reperfusion success rates and requires more technical adaptations ^4^. | Stent/Catheter: Prioritize devices with the lowest profile and high flexibility (e.g., Embotrap II 2.0 mm)  ^5^. Sub-millimeter magnetically steerable microcatheters represent future advancements ^6^.  Aspiration Catheter: Ultra-slim aspiration catheters (e.g., 3MAX, 4MAX). |
| Severe tortuosity of access vessels (ICA, aortic arch) | Tortuous vessels are more susceptible to injury, correlating with a higher post-procedural ICH risk ^5^.Tortuous ICA is an independent risk factor for spontaneous dissection ^7^. | SRs and CA (e.g., ADAPT) may improve the rate of first-pass effect (FPE). More flexible distal access catheters or long sheaths are recommended to enhance support ^5^. |
| Bifurcation Angle | | |
| Acute-angled bifurcation | Current evidence is limited; coronary bifurcation lesion strategies can serve as a reference. | The blind exchange/mini-pinning (BEMP) technique was compared with mini SRs alone ^8^. |

**References**

1.	Jing X, Nogueira RG, Nguyen TN, et al. Endovascular treatment in acute intracranial distal medium vessel occlusion stroke: Study protocol and rationale. *Int J Stroke* 2025;20(6):763-8.

2.	Mohammaden MH, Haussen DC, Pisani L, et al. Stent-retriever alone vs. aspiration and stent-retriever combination in large vessel occlusion stroke: A matched analysis. *Int J Stroke* 2022;17(4):465-73.

3.	Lefèvre T, Pan M, Stankovic G, et al. CTO and Bifurcation Lesions: An Expert Consensus From the European Bifurcation Club and EuroCTO Club. *JACC Cardiovasc Interv* 2023;16(17):2065-82.

4.	Knapen RR, Olthuis SG, van Es AC, et al. Association of first-line thrombectomy technique and outcome in late-window large vessel occlusion strokes: A post hoc analysis of the MR CLEAN-LATE trial. *Int J Stroke* 2024;19(10):1113-22.

5.	Koge J, Tanaka K, Yoshimoto T, et al. Internal Carotid Artery Tortuosity: Impact on Mechanical Thrombectomy. *Stroke* 2022;53(8):2458-67.

6.	Zhang M, Yang L, Yang H, et al. A magnetically actuated microcatheter with soft rotatable tip for enhanced endovascular access and treatment efficiency. *Sci Adv* 2025;11(25):eadv1682.

7.	Zhang L, Liu X, Gong B, et al. Increased Internal Carotid Artery Tortuosity is a Risk Factor for Spontaneous Cervicocerebral Artery Dissection. *Eur J Vasc Endovasc Surg* 2021;61(4):542-9.

8.	Pérez-García C, Moreu M, Rosati S, et al. Mechanical Thrombectomy in Medium Vessel Occlusions: Blind Exchange With Mini-Pinning Technique Versus Mini Stent Retriever Alone. *Stroke* 2020;51(11):3224-31.
