# Supplementary material for: Evidence, strategies, and pathways for thrombectomy in medium vessel occlusion stroke: a systematic review
Source: Front Neurol. 2026 Sep 29;17:1838008. doi: 10.3389/fneur.2026.1838008 (PMC13658856; doi:10.3389/fneur.2026.1838008)
Supplement: Supplementary file 4 [file Table_11.docx]

Supplementary Table S11. Imaging Phenotype-Guided Interventional Strategy

Evidence Levels: [High], RCT/Meta-analysis; [Moderate], Cohort/Registry Studies; [Low], Expert Consensus/Case Series.

Major Risks: Perforation, Distal Embolism, Vasospasm, sICH/ICH.

Abbreviations: ADAPT, A Direct Aspiration First-Pass Technique; SR, Stent Retriever; ICA, Intermediate Catheter Aspiration; BGC, Balloon Guide Catheter; FPE, First-Pass Effect; eTICI, expanded Thrombolysis in Cerebral Infarction; mRS, modified Rankin Scale; sICH, symptomatic Intracranial Hemorrhage; ICH, Intracranial Hemorrhage; ATD, Artery-to-Artery Thrombosis with underlying stenosis.

| Interventional Strategy Imaging Phenotype | ADAPT | SR (2-3 mm) | Combined Technique (SR+ICA) | Dedicated/Ancillary Technique |
| --- | --- | --- | --- | --- |
| Site of Occlusion | | | | |
| M2 Segment (Proximal/Trunk) High Thrombus Burden, Straight Path | Recommendation [Moderate] Evidence • eTICI ≥2b: ~87% • FPE: 37.3% • Time: Short Risk: sICH: 3.0% | Strong Recommendation [High] Evidence • eTICI ≥2b: ~88% • FPE: 62% • mRS 0-2: 42% Risk: Events: 24% | Preferred (High Burden) [High] Evidence • eTICI ≥2b: ~87% • FPE: 37.3% • Outcome: Superior Risk: ICH: 46% | Consider BGC. [Moderate] Evidence Advantage: Proximal flow control reduces the risk of distal embolism. |
| M2 Segment (Distal) / M3 Segment Small Lumen, Tortuous Path | Use with Caution [Moderate] Evidence Advantage: Single system, simplified operation. Risk: Difficult catheter access, increased risk of perforation. | Recommendation [Moderate] Evidence (e.g., Trevo 3mm) Advantage: High FPE (62%), suitable for tortuous vessels. Risk: Vasospasm, distal embolism. | Consider Cautiously [Low] Evidence Risk: Bulky system, high risk of perforation in small vessels. | Preferred: Dedicated Distal Access Catheter (e.g., Tenzing) [Moderate] Evidence (Settecase et al., 2024) • Successful Access Rate: >95% Advantage: Specifically designed for small, tortuous vessels; enables CA. Risk: Limited ability to handle very large thrombus burdens. |
| M4 Segment and Beyond Extremely Fine Vessels | Not Recommended Risk: Extremely high risk of perforation | Not Recommended Risk: Poor device deliverability, extremely high risk | Contraindicated | Only Option: Wire/Microcatheter System [Low] Evidence Technique: Microwire mechanical maceration, microcatheter superselective aspiration. Advantage: The only tools capable of reaching this location. Risk: Highest risk of perforation, inconsistent recanalization rate. |
| Thrombus Characteristics | | | | |
| "Soft" Thrombus (Contrast Penetration) | Strong Recommendation [Moderate] Evidence Advantage: Easy to aspirate, high FPE potential. | Effective [Moderate] Evidence | Effective [Moderate] Evidence | Preferred: BGC + Large-Bore Aspiration Catheter [Moderate] Evidence Advantage: Combines proximal flow control with maximized aspiration force for highest FPE. Risk: Vascular access injury, vessel trauma. |
| "Hard"/Resistant Thrombus | Risk: Aspiration failure, distal embolization. | Strong Recommendation [Moderate] Evidence Advantage: Stent anchor and integrate resistant thrombus. | Strong Recommendation [High] Evidence Advantage: Dual mechanism, high recanalization rate (95%), e.g., SAVE technique. | Consider: BGC [Moderate] Evidence Advantage: Prevents antegrade flow from causing thrombus dislodgment. |
| Vascular Anatomy | | | | |
| Underlying Stenosis (ATD) | Risk: Ineffective, prone to re-occlusion. | Risk: Elastic recoil, re-occlusion. | Risk: Re-occlusion. | Preferred: Microballoon Angioplasty ± Bailout Stenting [Moderate] Evidence Technique: Pre-dilation with low-profile microballoons (e.g., 2.0mm). Advantage: Targets the underlying cause, addresses the stenosis. Risk: Acute dissection, re-occlusion. |
| Failed First Pass Recanalization | | | | |
| Refractory Occlusion (After Failed First Attempt) | Consider Switching Technique | Consider: Dual SR Technique [Low] Evidence • eTICI ≥2b: 92.6% Risk: sICH: 5.5% | Consider: QUATTRO Technique [Low] Evidence • eTICI ≥2b: 98% Risk: Perforation: 2.0% | Last Resort: Wire Maceration / Local Thrombolysis [Low] Evidence Technique: Gentle thrombus maceration with microwire or superselective intra-arterial thrombolysis. Risk: Hemorrhagic transformation, perforation. |
