# Supplementary material for: Evidence, strategies, and pathways for thrombectomy in medium vessel occlusion stroke: a systematic review
Source: Front Neurol. 2026 Sep 29;17:1838008. doi: 10.3389/fneur.2026.1838008 (PMC13658856; doi:10.3389/fneur.2026.1838008)
Supplement: Supplementary file 5 [file Table_12.docx]

Supplementary Table S12. Interventional Strategy Mapping Table

Evidence Levels: A (High), RCT/Meta-analysis; B (Moderate), Cohort/Registry Studies; C (Low), Expert Consensus / Case Series.

Major Risks: P, Perforation/Dissection; E, Distal Embolism; S, Vasospasm; F, Failure.

Abbreviations: M1/M2/M3/M4, respective segments of the middle cerebral artery; ICA-T, internal carotid artery terminus; BA, basilar artery; SR, stent retriever; CAT, contact aspiration thrombectomy; FPE, first-pass effect; CA, contact aspiration.

| Occlusion Site | Collateral Circulation | Thrombus Burden/Characteristics | Access Difficulty | Preferred Technique | Evidence & Risks |
| --- | --- | --- | --- | --- | --- |
| M1 / ICA-T / BA (Large Vessel) | Poor (associated with rapid infarct core progression) | High / Stiff (e.g., High-Burden "T-Type" Thrombus) | Simple - Difficult | Combined Technique (SR + CA) | A [E, P]  • FPE: ~37%  •Final Reperfusion: >95% (with SAVE technique)  • Prefers highly effective reperfusion to reduce distal embolization. |
| Proximal M2 / M2 Main Trunk (Medium Vessel) | Any Collateral Status | Moderate to High | Simple to Moderate | SR (2-3 mm) or Combined Technique | A [E, S]  • FPE: 62% (with SR)  • Final Reperfusion: ~88%  • RSs offer superior grip on stiff thrombi. |
| Proximal M2 / M2 Main Trunk | Any Collateral Status | Low to Moderate (anticipated to be softer) | Simple to Moderate | **A Direct Aspiration First-Pass Technique (ADAPT)** | **B [E, F]**  **• FPE: ~37%**  **• Final Reperfusion: ~87%**  **•Advantages: Rapid and straightforward procedure.** |
| Distal M2/M3 | Any Collateral Status | Low to Moderate | Tortuous, Difficult Access | **Dedicated Distal Access Catheter (e.g., Tenzing)** | B [P, F]  • Successful Access Rate: >95%  • Designed for tortuous anatomy to achieve CA. |
| Distal M2/M3 | Any Collateral Status | Moderate to High | Simple to Moderate | Low-Profile SR (e.g., Trevo 3x20 mm) | **B [S, E]** • **FPE: 62%** • Provides high reperfusion rates when vessel diameter permits. |
| M4 and Beyond (Small Vessel) | Any Collateral Status | Low | Extremely Difficult | Wire/Microcatheter System (for mechanical disruption/super-selective thrombolysis) | **C [P]** •The only tool that can reach the site. High risk, variable recanalization rate. |
| Any Occlusion Site | Tandem Occlusion (e.g., ICAS with embolic occlusion) | With Underlying Intracranial Stenosis (ICAS) | Regardless of Access Complexity | Microballoon Angioplasty ± Bailout Stenting | **B [P]** •Targets the underlying etiology and resolves the stenosis. |
| Any Occlusion Site (After Failed First Attempt) | Refractory Occlusion | Very Stiff/Organized (Chronic) | Any Level of Difficulty | Dual SR or QUATTRO Technique | **B/C [P, S]** •**Final Reperfusion: 93-98%**  •Serves as an effective rescue strategy.。 |
