# Supplementary material for: Evidence, strategies, and pathways for thrombectomy in medium vessel occlusion stroke: a systematic review
Source: Front Neurol. 2026 Sep 29;17:1838008. doi: 10.3389/fneur.2026.1838008 (PMC13658856; doi:10.3389/fneur.2026.1838008)
Supplement: Supplementary file 6 [file Table_13.docx]

Supplementary Table S13. MeVO thrombectomy RCT design checklist.

Abbreviations: NIHSS, National Institutes of Health Stroke Scale; Tmax, time to maximum; sICH, symptomatic intracerebral hemorrhage; SAH, subarachnoid hemorrhage; mRS, modified Rankin Scale; MoCA, Montreal Cognitive Assessment.

| Design Elements | Implementation Checklist |
| --- | --- |
| Master Protocol and Common Control Group | Establish a unified master protocol that clearly defines common eligibility criteria, endpoints, and a shared statistical platform.  Implement a common control group to enable precise control for variables.  Develop distinct thrombectomy strategies while allowing simultaneous comparison against the common control group. |
| Stringent Enrollment Criteria | • Time Window: Within 24 hours of onset  • NIHSS Score: ≥5, or 3–5 with disabling deficits significantly impacting occupational or daily life  • Imaging Mismatch: Infarct core volume ≤50 mL, mismatch ratio (Tmax >6 s cerebral volume / ischemic core volume) ≥1.2, and mismatch volume ≥10 mL  • Exclusion: Presence of intracranial tumors, sICH, SAH, or perforation  • Pre-stratified randomization by dominant versus non-dominant hemisphere occlusion should be implemented to ensure balanced representation of this clinically important subgroup. |
| Rescue Therapy Restrictions | • Multiple unsuccessful thrombectomy attempts: If beyond 4–5 attempts, evaluate whether to continue the procedure.  • Intraoperative vascular perforation, severe sICH, or SAH: Requires immediate cessation for hemostasis to save the patient's life.  • Develop a standardized rescue strategy flowchart for the treatment arm, ensuring strict adherence to the protocol.  • Time from symptom onset to first pass attempt (not just door-to-puncture) should be mandatorily reported, as procedural time is frequently underreported in existing trials and is a critical determinant of outcome. |
| Arms Stopping and Adding | • A treatment arm may be stopped early if it demonstrates clear futility or superiority.  • New treatment arms may be added upon request, following predefined procedures, when emerging evidence supports their inclusion. |
| Operator and Adjudicator Credentialing | Requiring each certified center and every performing surgeon to complete a minimum of 15 stroke thrombectomy (ST) procedures annually as part of credentialing under tiered stroke center certification ^1, 2^. |
| Patient-Reported Outcome (PRO) | mRS score at 90 days, 90-day all-cause mortality, with cognitive function assessment (e.g., MoCA or equivalent) added given the eloquent cortex involvement in many MeVO cases. Inclusion of patient-reported outcomes beyond mRS at 90 days is recommended to capture domain-specific deficits that may not be adequately reflected by global disability scales. |

Note: This checklist represents the authors' conceptual synthesis of the methodological lessons derived from this review. It has not been empirically validated and should be regarded as a hypothesis-generating framework to inform the design of future MeVO thrombectomy trials.

**References**

1.	Stein LK, Mocco J, Fifi J, et al. Correlations Between Physician and Hospital Stroke Thrombectomy Volumes and Outcomes: A Nationwide Analysis. *Stroke* 2021;52(9):2858-65.

2.	Bulwa Z, Chen M. Stroke Center Designations, Neurointerventionalist Demand, and the Finances of Stroke Thrombectomy in the United States. *Neurology* 2021;97(20 Suppl 2):S17-s24.
