# Supplementary material for: Evidence, strategies, and pathways for thrombectomy in medium vessel occlusion stroke: a systematic review
Source: Front Neurol. 2026 Sep 29;17:1838008. doi: 10.3389/fneur.2026.1838008 (PMC13658856; doi:10.3389/fneur.2026.1838008)
Supplement: Supplementary file 7 [file Table_14.docx]

**Supplementary Table S14. Domain-level risk of bias assessment for included non-randomized studies using the Risk Of Bias In Non-randomized Studies of Interventions (ROBINS- I) tool.**

| Study | D1 | D2 | D3 | D4 | D5 | D6 | D7 | Overall | Weight |
| --- | --- | --- | --- | --- | --- | --- | --- | --- | --- |
| Abecassis IJ 2024 | Moderate | Low | Low | Low | Low | Moderate | Low | Moderate | 1 |
| Alessiani M 2025 | Moderate | Low | Low | Low | Low | Moderate | Low | Moderate | 1 |
| Dmytriw AA 2024 | Moderate | Low | Low | Low | Low | Moderate | Low | Moderate | 1 |
| Gaub M 2025 | Moderate | Low | Low | Low | Low | Moderate | Low | Moderate | 1 |
| Grossberg JA 2023 | Moderate | Low | Low | Low | Low | Moderate | Low | Moderate | 1 |
| Ishiguro T 2024 | Serious | Low | Low | Low | Low | Moderate | Low | Serious | 1 |
| Jing XZ 2025 | Moderate | Low | Low | Low | Low | Moderate | Low | Moderate | 1 |
| Meyer L 2024 | Moderate | Low | Low | Low | Low | Moderate | Low | Moderate | 1 |
| Meyer L 2021 | Moderate | Low | Low | Low | Low | Moderate | Low | Moderate | 1 |
| Meyer L 2023 | Moderate | Low | Low | Low | Low | Moderate | Low | Moderate | 1 |
| Meyer L 2022 | Moderate | Low | Low | Low | Low | Moderate | Low | Moderate | 1 |
| Mohammaden MH 2025 | Moderate | Low | Low | Low | Low | Moderate | Low | Moderate | 1 |
| Mohammaden MH 2024 DUSK | Moderate | Low | Low | Low | Low | Moderate | Low | Moderate | 1 |
| Nguyen TN 2024 PLATO | Moderate | Low | Low | Low | Low | Moderate | Low | Moderate | 1 |
| Orscelik A 2024 | Moderate | Low | Low | Low | Low | Moderate | Low | Moderate | 1 |
| Palmisano V 2025 | Moderate | Low | Low | Low | Low | Moderate | Low | Moderate | 1 |
| Pérez-García C 2020 | Moderate | Low | Low | Low | Low | Moderate | Low | Moderate | 1 |
| Rizzo F 2024 | Moderate | Low | Low | Low | Low | Moderate | Low | Moderate | 1 |
| Rosi A 2024 | Moderate | Low | Low | Low | Low | Moderate | Low | Moderate | 1 |
| Saber H 2022 | Moderate | Low | Low | Low | Low | Moderate | Low | Moderate | 1 |
| Salim HA 2026 | Moderate | Low | Low | Low | Low | Moderate | Low | Moderate | 1 |
| Salim HA 2024 MT-IVT | Moderate | Low | Low | Low | Low | Moderate | Low | Moderate | 1 |
| Salim HA 2025 EVT vs BMM | Moderate | Low | Low | Low | Low | Moderate | Low | Moderate | 1 |
| Salsano G 2024 | Moderate | Low | Low | Low | Low | Moderate | Low | Moderate | 1 |
| Siegler JE 2025 | Moderate | Low | Low | Low | Low | Moderate | Low | Moderate | 1 |
| Siegler JE 2023 | Moderate | Low | Low | Low | Low | Moderate | Low | Moderate | 1 |
| Zhang K 2026 | Moderate | Low | Low | Low | Low | Moderate | Low | Moderate | 1 |
| Nedelcu S 2023 | Moderate | Low | Low | Low | Low | Moderate | Low | Moderate | 1 |
| Shimonaga K 2025 | Moderate | Low | Low | Low | Low | Moderate | Low | Moderate | 1 |
| Hu D 2026 | Moderate | Low | Low | Low | Low | Moderate | Low | Moderate | 1 |
| Alawieh AM 2023 | Moderate | Low | Low | Low | Low | Moderate | Low | Moderate | 1 |
| Elhorany M 2022 | Moderate | Low | Low | Low | Low | Moderate | Low | Moderate | 1 |
| Nagel S 2020 | Moderate | Low | Low | Low | Low | Moderate | Low | Moderate | 1 |
| Mehta A 2025 | Moderate | Low | Low | Low | Low | Moderate | Low | Moderate | 1 |
| Valente I 2024 | Moderate | Low | Low | Low | Low | Moderate | Low | Moderate | 1 |
| Hofmeister J 2025 | Moderate | Low | Low | Low | Low | Moderate | Low | Moderate | 1 |
| Berberich A 2025 | Moderate | Low | Low | Low | Low | Moderate | Low | Moderate | 1 |
| MAD-MT HIR Analysis | Moderate | Low | Low | Low | Low | Moderate | Low | Moderate | 1 |
| Virtanen et al 2025 | Moderate | Low | Low | Low | Low | Moderate | Low | Moderate | 1 |
| Settecase et al 2024 | Moderate | Low | Low | Low | Low | Low | Low | Low | 1 |
| Schwartzmann et al 2024 | Moderate | Low | Low | Low | Low | Moderate | Low | Moderate | 1 |
| Yedavalli et al 2024 DMVO | Moderate | Low | Low | Low | Low | Moderate | Low | Moderate | 1 |
| Salim et al 2024 Aspirin | Moderate | Low | Low | Low | Low | Moderate | Low | Moderate | 1 |
| Salim et al 2024 ENT | Moderate | Low | Low | Low | Low | Moderate | Low | Moderate | 1 |
| Loehr et al 2024 ASSIST | Moderate | Low | Low | Low | Low | Moderate | Low | Moderate | 1 |
| Mohammaden et al 2024 BGC | Moderate | Low | Low | Low | Low | Moderate | Low | Moderate | 1 |
| Madaelil et al 2024 | Low | Low | Low | Low | Low | Low | Low | Low | 1 |
| Regenhardt et al 2024 | Moderate | Low | Low | Low | Low | Moderate | Low | Moderate | 1 |
| He et al 2025 | Moderate | Low | Low | Low | Low | Moderate | Low | Moderate | 1 |
| Hansen et al 2025 | Low | Low | Low | Low | Low | Low | Low | Low | 1 |
| Garcia et al 2024 | Moderate | Low | Low | Low | Low | Moderate | Low | Moderate | 1 |
| Salim et al 2024 EVT Failure | Moderate | Low | Low | Low | Low | Moderate | Low | Moderate | 1 |
| Amuluru et al 2024 | Moderate | Low | Low | Low | Low | Moderate | Low | Moderate | 1 |
| Yedavalli et al 2024 CBV | Moderate | Low | Low | Low | Low | Moderate | Low | Moderate | 1 |
| Mujanovic et al 2024 | Moderate | Low | Low | Low | Low | Moderate | Low | Moderate | 1 |
| Schäfer et al 2024 | Moderate | Low | Low | Low | Low | Moderate | Low | Moderate | 1 |
| German Stroke Registry 2024 | Moderate | Low | Low | Low | Low | Moderate | Low | Moderate | 1 |
| Guenego et al 2024 | Moderate | Low | Low | Low | Low | Moderate | Low | Moderate | 1 |
| Radu et al 2024 | Moderate | Low | Low | Low | Low | Moderate | Low | Moderate | 1 |
| Nome et al 2024 | Moderate | Low | Low | Low | Low | Moderate | Low | Moderate | 1 |
| Bo et al 2024 | Moderate | Low | Low | Low | Low | Moderate | Low | Moderate | 1 |
| Monteiro et al 2024 | Moderate | Low | Low | Low | Low | Moderate | Low | Moderate | 1 |
| Matsukawa et al 2024 | Moderate | Low | Low | Low | Low | Moderate | Low | Moderate | 1 |
| Kuwahara et al 2024 | Moderate | Low | Low | Low | Low | Moderate | Low | Moderate | 1 |
| Dmytriw et al 2024 Perforation | Moderate | Low | Low | Low | Low | Low | Low | Low | 1 |
| Schwarz et al 2023 | Moderate | Low | Low | Low | Low | Moderate | Low | Moderate | 1 |
| Enriquez et al 2023 | Moderate | Low | Low | Low | Low | Moderate | Low | Moderate | 1 |
| TOPMOST 2024 | Moderate | Low | Low | Low | Low | Moderate | Low | Moderate | 1 |
| TOPMOST 2024 Secondary | Moderate | Low | Low | Low | Low | Moderate | Low | Moderate | 1 |
| Guenego et al 2022 | Moderate | Low | Low | Low | Low | Moderate | Low | Moderate | 1 |
| Roussel et al 2022 | Moderate | Low | Low | Low | Low | Moderate | Low | Moderate | 1 |
| BEMP Technique Study 2022 | Moderate | Low | Low | Low | Low | Moderate | Low | Moderate | 1 |
| Bala et al 2024 | Low | Low | Low | Low | Low | Low | Low | Low | 1 |
| REVISAR Registry 2024 | Low | Low | Low | Low | Low | Low | Low | Low | 1 |
| TOPMOST 2023 | Low | Low | Low | Low | Low | Low | Low | Low | 1 |
| Yedavalli et al 2024 HT | Moderate | Low | Low | Low | Low | Low | Low | Low | 1 |
| Shi et al 2023 | Moderate | Low | Low | Low | Low | Moderate | Low | Critical | 1 |

Abbreviations: ROBINS‑I = Risk Of Bias In Non-randomized Studies of Interventions. D1 = Bias due to confounding. D2 = Bias in selection of participants into the study. D3 = Bias in classification of interventions. D4 = Bias due to deviations from intended interventions. D5 = Bias due to missing data. D6 = Bias in measurement of the outcome. D7 = Bias in selection of the reported result. Overall = Overall risk of bias judgement.

Risk of bias judgement categories: Low = Low risk of bias. Moderate = Moderate risk of bias. Serious = Serious risk of bias. Critical = Critical risk of bias.

Notes: All included studies were assessed independently by two reviewers. Disagreements were resolved through discussion or consultation with a third reviewer.
