# Supplementary material for: Evidence, strategies, and pathways for thrombectomy in medium vessel occlusion stroke: a systematic review
Source: Front Neurol. 2026 Sep 29;17:1838008. doi: 10.3389/fneur.2026.1838008 (PMC13658856; doi:10.3389/fneur.2026.1838008)
Supplement: Supplementary file 8 [file Table_15.docx]

Supplementary Table S15. Characteristics of Included Studies

Abbreviations: BEMP, balloon-guided enhanced microcatheter protection; BGC, balloon guide catheter; BMT, best medical management/best medical therapy/medical management; CBV, cerebral blood volume; CE-CBCT, contrast-enhanced cone-beam CT; CS, conscious sedation; CTA, CT angiography; CTP, CT perfusion; DSA, digital subtraction angiography; DRA, digital subtraction angiography (Note: typically synonymous with DSA); END, early neurological deterioration; SR, stent retriever; CA, contact aspiration; ENT, embolization to new territory; eTICI, expanded thrombolysis in cerebral infarction; EVT, endovascular therapy/mechanical thrombectomy; FIV, final infarct volume; FPE, first-pass effect; GA, general anesthesia; HIR, hypoperfusion intensity ratio; IVT, intravenous thrombolysis; LA, local anesthesia; MRA, MR angiography; mTICI, modified thrombolysis in cerebral infarction; NCCT, non-contrast CT.

| Study (First Author, Year) | Study Design | Country / Centers | MeVO Definition (Original) | Occlusion Sites Included | Key Inclusion Criteria | Exclusion of Proximal LVO? | EVT Technique Details | Control Intervention (if any) | Rescue Therapy Permitted? | Primary Outcome | Primary Outcome Result | Safety Outcomes (sICH, mortality) | Other Key Findings | Imaging Modality（CTA/MRI/DSA） | Time Window (mean or median) | Risk of Bias (Tool & Judgment) | Notes |
| --- | --- | --- | --- | --- | --- | --- | --- | --- | --- | --- | --- | --- | --- | --- | --- | --- | --- |
| Abecassis IJ, 2024 | Multicenter retrospective cohort | 29 centers, mostly USA | Distal occlusions not included in major MT trials | A1/A2, M2, M3/4, P1/2 | NIHSS ≤5, underwent MT | Yes (proximal LVO excluded) | ADAPT 39.4%, SR 22.0%, Combined 35.6% | None (single-arm) | Yes (combined as rescue) | 90d mRS 0-2 | 64.6% | sICH 6.9%, mortality 8.8% | Distal occlusion associated with NIHSS improvement (aOR 2.03); ADAPT better than Combined for 90d outcome | CTA / DSA | Onset-to-puncture 447±394 min | ROBINS-I: Moderate | Data extracted only for distal occlusion subgroup (n=257) |
| Alessiani M, 2025 | Retrospective observational study (single center) | Italy, San Camillo Forlanini Hospital (Rome), single center | DMVOs defined as occlusion in A2, A3, distal M2 or its single branch, M3, P2, P3, PICA, SCA, AICA | A2, A3, distal M2 or its single branch, M3, P2, P3, PICA, SCA, AICA | AIS with primary or secondary DMVO; treated with MT alone or IVT alone; age ≥18 years | Yes (excluded ICA, M1, tandem lesions, proximal/dominant M2, VA/BA, and patients with concomitant LVO plus DMVO) | Direct aspiration (ADAPT) – all MT patients treated with direct aspiration; 17/59 MT patients also received bridging IVT | IVT alone (alteplase) | Not reported (no mention of rescue therapy) | Safety and efficacy of MT alone vs IVT alone in DMVO (change in NIHSS from admission to discharge, 3-month mRS, 3-month mortality rate, recanalization rate, hemorrhagic transformation, procedural complications) | After propensity score adjustment: no significant differences between MT and IVT groups for 3-month mRS>2 (p=0.270), ∆NIHSS (p=0.614), hemorrhagic transformation (p=0.260), or mortality (p=0.813). MT achieved mTICI≥2b in 95% of cases. | Hemorrhagic transformation: MT 33.8% vs IVT 20.8% (p=0.135, after PSM p=0.260) – not strictly sICH. 90-day mortality: MT 11.8% vs IVT 14.5% (p=0.678, after PSM p=0.813). No procedural complications in MT group. | Secondary DMVO (n=39) had worse 3-month mRS>2 (56.4% vs 29.4%, p=0.006) and higher hemorrhagic transformation (43.5% vs 19.1%, p=0.007) than primary DMVO. In primary DMVO subgroup (n=68), MT vs IVT showed no significant differences. | CT/CTA (perfusion used for extended window, not detailed) | Onset-to-treatment time (OTT): MT 245.88±100.78 min, IVT 203.97±77.78 min | ROBINS-I V2: Moderate (retrospective design, selection bias, small sample size in subgroups, despite propensity score adjustment) | Single-center Italian real-world data; distinguishes primary vs secondary DMVO; MT group included 17 bridging patients; no procedural complications reported in MT group; hemorrhagic transformation rate higher than typical sICH definition |
| Clarençon F, 2024 (DISCOUNT) | Phase 3, multicenter, prospective, randomized, open-label, blinded-outcome (PROBE) trial — Terminated early | France, 22 comprehensive stroke centers | Distal M2 (above mid-height of insula), M3; A1, A2, A3; P1, P2, P3 | Distal M2, M3; A1–A3; P1–P3 | NIHSS ≥5; distal occlusion; within 6h (or ≤24h if MRI FLAIR negative) | Yes | Stent retriever (≤3mm) and/or aspiration catheter; maximum 3 passes | Best medical treatment (BMT) alone (including IVT if eligible) | No (protocol capped at 3 passes; no intracranial stenting for refractory occlusion) | Good clinical outcome (mRS 0–2) at 90 days | Trial stopped early after pre-specified interim analysis: harm signal in EVT arm (worse functional outcomes, higher mortality, more sICH) | Complete peer-reviewed results not yet published; interim analysis indicated increased sICH and mortality in EVT arm | This trial provides important cautionary evidence against routine EVT in non-selected distal occlusion patients. | CT or MRI (local standard); DSA for EVT | Within 6h (or ≤24h if MRI FLAIR negative) | RoB 2: Some concerns (incomplete outcome data due to early termination) | Results are based on the published protocol and public reports of early termination. Final peer-reviewed data are unavailable. |
| Dmytriw AA, 2024 | Retrospective multicenter cohort study (37 academic centers in North America, Asia, Europe) | 37 centers across North America, Asia, Europe | Based on Saver et al. (Stroke 2020) definition of medium vessel occlusion (medium-proximal or primary medium-distal vessels) | Not explicitly listed in abstract/methods but likely includes distal M2, M3, A2, A3, P2, P3, etc. (see Table S2 for details) – from context, medium vessel occlusions including M2, M3, A2, A3, P2, P3 | Consecutive adult patients with AIS due to medium vessel occlusion treated with MT alone or MT+IVT; data from Sep 2017 to Jul 2021 | Yes (excluded patients with intra-arterial thrombolysis, not undergoing MT; study specifically includes medium vessel occlusion, proximal LVO excluded by definition) | MT alone vs MT+IVT. MT devices: aspiration and/or stent retriever per operator discretion (see Table S1 for device types) | MT alone (no IVT) | Not explicitly reported; likely at operator discretion (salvage techniques allowed as per standard practice) | 90-day modified Rankin Scale (mRS) score 0–2 | In multivariable analysis after propensity score matching (n=670), addition of IVT was not significantly associated with mRS 0-2 (OR, 1.37; 95% CI: 0.99, 1.89; P = .054). Unadjusted OR was 1.44 (1.06-1.96, P=0.019) but not significant after adjustment. | sICH not specifically reported; any intracranial hemorrhage: OR 1.25 (0.88-1.76, P=0.21) for MT+IVT vs MT alone. 90-day mortality: OR 0.78 (0.49-1.24, P=0.29). Both not significant. | No difference in successful reperfusion (TICI 2b-3: aOR 1.64, P=0.055), first-pass effect (aOR 1.27, P=0.17), or number of passes. MT alone group had longer onset-to-puncture (350 vs 210 min, P<0.001) and onset-to-recanalization (397 vs 273 min, P<0.001). | CTA, DSA (angiography), and noncontrast CT; MRI used for follow-up (diffusion-weighted MRI shown in Figure 2). | Median onset-to-puncture: MT alone 350 min (IQR not given), MT+IVT 210 min (P<0.001); median onset-to-recanalization: 397 vs 273 min. | ROBINS-I V2: Moderate (retrospective design, despite propensity score matching, potential residual confounding; no core lab adjudication for imaging outcomes) | Large international registry (MAD-MT Consortium); primary comparison is MT+IVT vs MT alone (not IVT alone). Propensity score matching performed on all Table 1 variables. Primary outcome not significant after adjustment. No separate analysis of primary vs secondary MeVO. |
| Gaub M, 2025 | Retrospective multicenter registry analysis (STAR registry) | International, 35 participating centers (primarily US, plus Europe, Asia, South America) | M2 segment occlusion based on IMS III anatomical criteria: MCA occlusions distal to the lenticulostriates with antegrade filling of at least one classic M2 branch (post-bifurcation frontal or temporal divisions, pre-bifurcation posterior temporal or holotemporal branches). | M2 trunk, frontal division, temporal division | Acute ischemic stroke due to MCA M2-segment occlusion; underwent mechanical thrombectomy; data from Jan 2013 – Jun 2024 | Yes (M1 occlusions excluded by definition; M2 occlusions specifically selected) | Frontline aspiration (direct aspiration first pass technique, ADAPT) vs stent retriever alone or combined (stent retriever + aspiration) | Not applicable (comparison of two EVT techniques, not EVT vs medical therapy) | Yes (at operator discretion; details not specified) | Functional independence (modified Rankin Scale score 0–2) at 90 days | After propensity score matching (n=711 per group): aspiration 49.9%, SR/combined 44.0%; OR 1.27 (95% CI 1.03–1.57), p=0.02 | sICH: aspiration 3.5%, SR/combined 6.2%; OR 0.55 (0.33–0.91). 90-day mortality: aspiration 19.1%, SR/combined 17.9%; OR 1.09 (0.83–1.42), p=0.52 (not significant) | Complete recanalization (TICI 2c–3) favored aspiration (61.2% vs 48.7%, OR 1.66). Complete first-pass effect favored aspiration (35.0% vs 27.6%, OR 1.42). Procedure time shorter with aspiration (median 28 vs 51 min). Subgroup analysis: temporal M2 and left-sided occlusions derived greater benefit from aspiration. Each minute increase in procedure time reduced functional independence probability less with aspiration (0.35% vs 1.61% per minute, p<0.001). | CTA, DSA, noncontrast CT (ASPECTS), MRI not specified | Median onset-to-arterial puncture: not reported separately for matched cohort; unmatched: aspiration 444.5±426.8 min, SR/combined 459±786.3 min (p=0.648 after matching). Procedural time (puncture-to-recanalization): aspiration median 28 min (IQR 15–49.5), SR/combined 51 min (IQR 35–78), p<0.001 | ROBINS-I V2: Moderate (retrospective registry, operator-dependent technique selection, no core lab adjudication for imaging outcomes, despite propensity score matching) | Study compares frontline aspiration vs stent retriever/combined for M2 occlusions, not EVT vs medical management. Useful for technical sub-analysis of MeVO. Large sample size (n=1734). Propensity score matching used but procedure time not matched, which may bias outcomes. |
| Goyal M, 2025 | Phase 3, multicenter, prospective, randomized, open-label, blinded‑outcome (PROBE) trial | 5 countries (Canada, USA, Germany, UK, Hungary, etc.) – 58 sites | Occlusion of M2 or M3 segment of MCA, A2 or A3 of ACA, or P2 or P3 of PCA (explicitly excludes A1, P1). Proximal M2: from main bifurcation to 1 cm distal; distal M2: thereafter to circular sulcus; M3: opercular segment. | M2 (proximal & distal), M3, A2, A3, P2, P3 | Adults with AIS due to MeVO; NIHSS >5 or 3‑5 with disabling deficit; within 12 h of last known well; favorable baseline imaging (no large infarct, good collaterals or perfusion mismatch); pre‑stroke mRS 0‑1 | Yes (ICA, M1, A1, P1, vertebral, basilar occlusions excluded) | First-line: Solitaire X stent retriever (± aspiration). Subsequent attempts at operator discretion using any approved device or combination. | Guideline‑based usual care alone (includes IVT if indicated, stroke unit care, early rehab, secondary prevention) | Yes – after first attempt, any approved device allowed | Modified Rankin Scale score 0‑1 (excellent functional outcome) at 90 days | EVT+usual care: 41.6% (106/255); usual care alone: 43.1% (118/274); adjusted rate ratio 0.95 (0.79‑1.15), P=0.61 (not significant) | sICH: EVT 5.4% (14/257), control 2.2% (6/272). 90‑day mortality: EVT 13.3% (34/255), control 8.4% (23/274); adjusted HR 1.82 (1.06‑3.12) | mRS 0‑2: 54.1% vs 58.8% (aRR 0.92). Barthel ≥95: 53.5% vs 64.7% (aRR 0.81). Infarct volume median: 31.9 vs 29.1 ml. Serious AEs: 33.9% vs 25.7%. No quality‑of‑life difference. Recanalization (MeVO‑eTICI 2b‑3) 75.1%. Higher mortality and sICH with EVT. | Noncontrast CT or MRI (ASPECTS), CTA or MRA (collaterals), perfusion CT or MR (mismatch) – per local protocol; DSA for EVT guidance | Onset‑to‑randomization median: EVT 270 min (IQR 160‑438), control 253 min (148‑396). Onset‑to‑recanalization median: 359 min (EVT group). | RoB 2: Low – randomized, blinded outcome assessment, minimal missing data, intention‑to‑treat analysis, protocol deviations balanced. | Landmark negative RCT for MeVO EVT. Majority (84.7%) had M2/M3 occlusions. Higher mortality and sICH in EVT group. No benefit of EVT over medical management alone within 12 h. |
| Grossberg JA, 2023 (published online 2022; print 2023) | International multicenter retrospective registry study (STAR registry) | 32 centers (global, primarily US and Europe) | Proximal medium vessel occlusion (pMeVO): M2 segment of MCA, A1 segment of ACA, P1 segment of PCA (specifically includes A1 and P1, which are often considered proximal segments) | M2, A1, P1 | Adult AIS patients undergoing MT for pMeVO (as defined) between 2015‑2021; data from STAR registry | Yes – excludes ICA, M1, vertebrobasilar LVO. Also excludes distal MeVO (M3, A2, P2, etc.) and rescue thrombectomy after prior intervention. | Frontline aspiration (ADAPT) vs stent retriever (SRT) vs primary combined (PC). Operator preference. | Not applicable (comparison of EVT techniques, not EVT vs medical therapy). Also compares pMeVO vs LVO outcomes. | Yes – at operator discretion after frontline technique (not specified further) | 90‑day good functional outcome (modified Rankin Scale score 0‑2) | In propensity‑matched pMeVO cohort: ADAPT 45%, SRT/PC 47% (p > 0.05). No significant difference between techniques. | sICH/PH2: ADAPT 4%, SRT/PC 9% (p < 0.01, favoring ADAPT). 90‑day mortality: not significantly different between techniques (rates not separately reported for pMeVO; overall ~ not given). | Independent predictors of good outcome in pMeVO: successful recanalization (aOR 3.2), procedure time ≤1 h (aOR 2.2), attempts ≤4 (aOR 2.8). “Golden hour” (≤60 min) and “3‑pass” rules apply to pMeVO. Procedure time shorter with ADAPT (33 vs 51 min, p<0.001); number of attempts higher with ADAPT (2.5 vs 2, p<0.001). Futility analysis: ADAPT outcomes more sensitive to procedure time, SRT/PC more sensitive to number of attempts. | Noncontrast CT (ASPECTS), CTA / MRA, DSA for EVT; 24h CT or MRI for hemorrhage | Onset‑to‑groin (matched cohort): ADAPT 412±380 min, SRT/PC 440±430 min (p>0.05). Procedure time (puncture‑to‑recanalization): ADAPT 32.8±29 min, SRT/PC 51.4±33 min (p<0.001). | ROBINS‑I V2: Moderate (retrospective registry, no core lab adjudication, operator‑dependent technique selection, propensity score matching used but residual confounding possible) | pMeVO definition includes A1 and P1 (proximal segments). No comparison with medical management. Shows comparable outcomes between techniques but safety (sICH) favored ADAPT. Procedural time and attempts have differential impact by technique. |
| Ishiguro T, 2024? (在线发表于2024，PMCID: PMC12832319，具体卷期未提供) | Prospective single‑center observational study (before‑after design: conventional SRs until July 2023, then Tron FX II exclusively) + bench testing | Japan, single center (Tokyo Women’s Medical University) | Occlusions located in MCA M2 segment or beyond (M3, etc.), ACA, PCA – defined as medium vessel occlusion (MeVO). Large vessel occlusion (LVO) includes ICA terminus, M1, vertebral or basilar artery. | M2, M3, ACA, PCA (MeVO subgroup); also includes M1 and ICA (LVO) but reported separately | Consecutive patients undergoing MT for acute ischemic stroke between April 2021 and March 2025; combined technique (stent retriever + aspiration) used as first‑line; Tron used exclusively after July 2023 | Not excluded overall; but analysis of MeVO + M1 occlusion combined was performed separately. The MeVO definition excludes M1, ICA, vertebrobasilar. | Combined technique (stent retriever + aspiration catheter + balloon guiding catheter) for all patients. Conventional SRs: Solitaire X, Trevo NXT, EmboTrap III; Tron FX II (low radial/axial force) used after July 2023. | Historical control group using conventional stent retrievers (treated before July 2023) | Yes – up to 3 passes with same device; not specified beyond that | Incidence of post‑procedural subarachnoid hemorrhage (SAH) assessed by dual‑energy CT immediately after MT, with or without symptoms | Overall SAH: Tron group 6.6% (5/76), conventional SR group 19.8% (24/121), p=0.012. In MCA occlusion + MeVO combined: Tron 7.7% vs 23.9%, p=0.027. Symptomatic SAH (with neurological deterioration) in MCA+MeVO: Tron 1.9% vs 12.7%, p=0.044. | Symptomatic SAH (≥4 NIHSS increase): overall Tron 1.3% vs conventional 7.4%, p=0.092 (not significant). 90‑day mortality not reported. No separate sICH (parenchymal hematoma) reported; focus on SAH. | Bench testing: Tron had lowest radial force, axial flexibility, retrieval traction force, and vessel deviation. Multivariate analysis: Tron use (OR 0.25) and fewer SR passes (OR 1.72 per pass) independently associated with lower SAH risk. No difference in recanalization (effective recan 92.1% vs 90.1%), first‑pass rate (48.7% vs 51.2%), or 90‑day good outcome (32.9% vs 25.6%, p=0.33). | Noncontrast CT (ASPECTS), CTA for occlusion site; dual‑energy CT (virtual noncontrast) for SAH assessment immediately post‑MT; DSA for thrombectomy | Puncture‑to‑recanalization time (mean±SD): Tron 45.8±24.5 min, conventional 52.7±31.0 min (p=0.080). Onset‑to‑puncture not reported. | ROBINS‑I V2: Serious (non‑randomized before‑after design, potential time‑period bias, operator experience may improve over time, no adjustment for temporal trends, small sample size for MeVO subgroup, no core lab adjudication for SAH although blinded assessment by three neuroradiologists) | Pilot study with bench testing. Primary outcome is post‑procedural SAH (not sICH). No comparison with medical management. Combined technique used in all patients. The study suggests low‑force SR may reduce SAH without compromising recanalization. MeVO subgroup analysis limited by small numbers (M2, ACA, PCA). |
| Hu W, 2026 (N Engl J Med 2026;394:1894-904) | Phase 3, multicenter, prospective, randomized, open‑label, blinded‑outcome (PROBE) trial | China, 48 centers | Occlusion of codominant or nondominant M2 or M3 segment of MCA; A1, A2, or A3 of ACA; P1, P2, or P3 of PCA. Codominant/nondominant M2 diameter ≤2.0 mm. Excludes dominant M2. | M2 (codominant/nondominant), M3, A1‑A3, P1‑P3 | Adult (≥18), pre‑stroke mRS 0‑2, NIHSS ≥6, within 24 h of last known well, occlusion of MeVO per definition, imaging showing <50% ischemic involvement of at‑risk territory or perfusion mismatch (penumbra/core ratio >1.4 and mismatch volume ≥10 ml) | Yes – excludes ICA, M1, vertebral, basilar occlusions. Also excludes dominant M2 and proximal segments (A1? Actually A1 included as MeVO per definition, but M1 excluded) | Operator discretion: stent retriever, aspiration, combined, angioplasty, stents, intraarterial thrombolysis. First‑pass most common combined SR+aspiration (46.9%). General anesthesia used in 33.9%. | Medical management alone (standard care per guidelines: antiplatelet, IVT if eligible, etc.) | Yes – at operator discretion; multiple passes and adjunctive techniques allowed | Functional independence (modified Rankin Scale score 0, 1, or 2) at 90 days (original planned ordinal shift violated proportional odds assumption) | Thrombectomy group: 58.6% (164/280); control group: 46.6% (132/283); adjusted rate ratio 1.24 (95% CI 1.07–1.44), P=0.004 | sICH (SITS‑MOST criteria): thrombectomy 4.7% (13/275), control 2.2% (6/271); adjusted risk ratio 2.21 (0.87–5.63). 90‑day mortality: thrombectomy 11.1% (31/280), control 10.2% (29/283); adjusted risk ratio 1.11 (0.70–1.76) | Excellent outcome (mRS 0‑1): 48.9% vs 33.2% (aRR 1.47). Successful reperfusion (eTICI 2b50‑3): 74.4%. Patency at 24‑72h: 82.1% vs 46.2%. Any ICH: 11.4% vs 6.0%. Subgroup analysis suggested benefit in NIHSS ≥8, M2, A, P occlusions, but not in M3 or time ≥8h. | CTA or MRA for occlusion; noncontrast CT or DWI for infarct; CT or MR perfusion for mismatch; DSA for EVT; follow‑up CTA/MRA at 24‑72h | Onset‑to‑randomization median: 5.0 h (IQR 3.4‑8.2) in both groups. Onset‑to‑arterial access median: 5.3 h. Onset‑to‑revascularization median: 6.4 h. Puncture‑to‑revascularization median: 1.0 h (IQR 0.7‑1.6). | RoB 2: Low – randomized, blinded outcome assessment (central adjudication), independent statistician, intention‑to‑treat analysis, minimal missing data (11/563), prespecified primary outcome change due to proportional odds violation was done before analysis. | Positive RCT for MeVO EVT in patients with moderate‑to‑severe deficits (NIHSS ≥6). Population predominantly Chinese with high large‑artery atherosclerosis etiology. Lower IVT use (36.6%) than previous trials. M2 diameter restriction ≤2 mm for nondominant/codominant. Mortality no difference, sICH higher but not statistically significant. |
| Jing XZ, 2025 (Radiology 2025;317(2):e251040) | Multicenter retrospective registry study | China, 25 stroke centers across 14 provinces | Distal medium vessel occlusion (DMVO) – excludes M2 (even nondominant) and proximal segments. Based on lumen diameter 0.75–2.0 mm per consensus (Saver et al.). | M3, M4 of MCA; A1, A2, A3 of ACA; P1, P2, P3 of PCA (specifically excludes M2) | Acute ischemic stroke due to isolated primary DMVO (as defined), pre‑stroke mRS ≤2, time from onset to treatment <24 h, data from Sep 2019 – Sep 2024 | Yes – excludes ICA, M1, M2 (even nondominant), vertebral/basilar, and multivessel occlusions | Operator discretion: combined (47.2%), aspiration only (30.9%), stent retriever only (21.9%). First‑line strategy at discretion. Successful reperfusion (mTICI ≥2b‑3) in 85.8%. | Standard medical management (SMM) alone per Chinese guidelines (IVT if eligible, antiplatelet/anticoagulant, risk factor management) | Not explicitly reported; at operator discretion within EVT group | Ordinal shift in 90‑day modified Rankin Scale (mRS) score distribution | IPTW analysis: adjusted common OR 1.38 (95% CI 1.18–1.61), P<0.001 favoring EVT. Also EVT had higher mRS 0‑1 (43.7% vs 36.1%, aRR 1.61) and mRS 0‑2 (60.9% vs 53.5%, aRR 1.39). | sICH (NIHSS increase ≥4): EVT 11.5% (61/529), SMM 10.4% (57/546); adjusted RR 1.24 (0.92–1.66), P=0.16. 90‑day mortality: EVT 8.3% (44/529), SMM 8.9% (49/546); adjusted RR 0.74 (0.51–1.12), P=0.17. | Subgroup: EVT benefit in NIHSS ≥6 (aRR 1.62) but not in NIHSS 1‑5 (aRR 0.79), interaction P<0.001. Successful reperfusion strongly associated with good outcomes (mRS 0‑1 aRR 2.78, mRS 0‑2 aRR 3.22). No difference in safety by first‑line device. | CTA / MRA for occlusion; noncontrast CT / DWI; DSA for EVT; follow‑up imaging not described for SMM group | Median onset‑to‑admission: 376 min (IQR 234‑545); onset‑to‑treatment for EVT not separately reported. Inclusion within 24 h. | Median onset‑to‑admission: 376 min (IQR 234‑545); onset‑to‑treatment for EVT not separately reported. Inclusion within 24 h. | Largest DMVO study excluding M2 (n=1075). Positive for EVT vs medical management. High sICH rate (11.5% vs 10.4%) but not significant. Low IVT use (22.1%) compared to RCTs. Population Asian, high large‑artery atherosclerosis. |
| Meyer L, 2024 | International, retrospective, multicenter observational registry (TOPMOST) | Europe (Germany, Switzerland, France, Spain, Belgium, etc.), USA, Asia (Singapore); multiple centers | Primary isolated distal medium vessel occlusions (DMVOs) of posterior and anterior cerebral arteries: P2/P3 segments of PCA, A2–A4 segments of ACA | PCA: P2, P3; ACA: A2, A3, A4 (excludes MCA, M2, etc.) | Acute ischemic stroke due to primary isolated DMVO (PCA or ACA), endovascular treatment, data from Jan 2010 – Oct 2021 | Yes – excludes proximal LVO (ICA, M1, basilar, vertebral, P1, A1, etc.) | Aspiration catheters, stent retrievers, intra-arterial thrombolysis, at operator discretion | Not applicable – comparison of anesthetic strategies: local anesthesia ± conscious sedation (LA±CS) vs general anesthesia (GA) | Yes – multiple attempts allowed; details not specified | Rate of complete reperfusion (modified Thrombolysis in Cerebral Infarction score 3, mTICI 3) on final angiography | LA±CS: 73.9% (88/119), GA: 71.9% (82/114); P=0.729 (no significant difference). In ACA DMVO subgroup, GA favored over LA±CS (aOR 3.07, 95% CI 1.24–7.57, P=0.015). | sICH (ECASS II): LA±CS 2.5% (3/119), GA 1.8% (2/114); P=1.0. 90‑day mortality: LA±CS 15.7% (13/119), GA 17.2% (15/114); P=0.913. | No difference in successful reperfusion (mTICI 2b‑3), first‑pass effect, procedure time, or number of attempts. Functional outcome (mRS 0‑1) univariable favored LA±CS but not after adjustment. Anterior circulation site associated with worse functional outcome. | CTA, MRA, DSA | Median symptom onset to groin puncture: 196 min (IQR 145–300) | ROBINS‑I V2: Moderate (retrospective, non‑randomized, selection bias likely, no core lab for mTICI, missing data, but multivariable adjustment) | Study compares anesthetic strategies, not EVT vs medical management. Only primary isolated PCA and ACA occlusions; MCA MeVO excluded. GA group had higher baseline NIHSS and more ACA occlusions. GA may benefit ACA DMVO for complete reperfusion. |
| Meyer L, 2021 | International, retrospective, multicenter case‑control study (propensity score matched 1:1) | Europe (Germany, Switzerland, France, Spain, Belgium, Sweden, etc.), USA, Asia (Singapore); 23 comprehensive stroke centers | Primary isolated distal medium vessel occlusion (DMVO) of the posterior cerebral artery (PCA): P2 segment (from PCA–posterior communicating artery branch point to quadrigeminal cistern) and P3 segment (from quadrigeminal cistern to calcarine fissure) | PCA: P2, P3 (excludes P1, excludes anterior circulation MeVOs, excludes MCA) | Acute ischemic stroke due to isolated PCA P2/P3 occlusion; treated with mechanical thrombectomy or standard medical treatment (±IVT); data from Jan 2010 – Jun 2020 | Yes – excludes PCA P1, basilar, vertebral, M1, ICA, etc. Also excludes proximal large vessel occlusions. | Stent retriever with/without aspiration (72.0%), primary aspiration (26.6%), intra‑arterial tPA (1.4%). First‑pass effect rate 45.5%. General anesthesia in 43.3%. | Standard medical treatment with or without intravenous thrombolysis (IVT) | Not explicitly reported; multiple thrombectomy attempts allowed (median 1–2 passes) | Improvement in National Institutes of Health Stroke Scale (NIHSS) score from baseline to discharge (mean change, early neurological improvement) | Mean NIHSS decrease: EVT −3.9 (95% CI −5.4 to −2.5), medical −2.4 (95% CI −3.2 to −1.6); mean difference −1.5 (95% CI −3.2 to 0.8), P=0.06 (not significant). In subgroups: NIHSS ≥10 (mean diff −5.6, P=0.04) and no IVT (mean diff −3.0, P=0.005) favored EVT. | sICH (ECASS II): EVT 4.3% (4/92), medical 4.3% (4/92), P>0.99. 90‑day mortality: EVT 11.8% (9/76), medical 15.8% (9/57), P=0.40 (not significant).In‑hospital mortality: 4.4% vs 5.4%. | Successful reperfusion (mTICI 2b‑3): 87.4%; complete reperfusion (mTICI 3): 76.2%. Excellent functional outcome (mRS 0‑1) at 90 days: EVT 66.2% vs medical 54.4% (P=0.16). First‑pass effect associated with early neurological improvement (aOR 2.32). No significant difference in functional independence (mRS 0‑2) at 90 days. | CTA, MRA, DSA; MRI or CT with angiography/perfusion | Median onset‑to‑groin puncture (EVT): 197 min (IQR 148‑277); onset‑to‑imaging (medical): 141 min (IQR 104‑216); onset‑to‑IVT (medical): 151 min (IQR 120‑201) | ROBINS‑I V2: Moderate (retrospective, non‑randomized, selection bias despite PSM, missing data, no core lab for imaging, but propensity matching performed) | Primary outcome was NIHSS change (not mRS). Only PCA P2/P3 occlusions; anterior circulation MeVOs and M2/M3 excluded. EVT was safe and technically feasible but did not significantly improve 90‑day mRS. Subgroup analyses suggested benefit in higher NIHSS (≥10) and non‑IVT patients. |
| Meyer L, 2023 | International, retrospective, multicenter case‑control study (propensity score matched 1:1) | Europe (Germany, Switzerland, France, Spain, etc.), USA, Asia (Singapore); multiple comprehensive stroke centers (TOPMOST registry) | Primary isolated distal medium vessel occlusion (DMVO) of the anterior cerebral artery (ACA) distal to the A1 segment: A2 segment (anterior communicating artery to dorsal middle convex arch), A3 segment (around genu of corpus callosum), A4 segment (frontal portion running backward within median fissure) | ACA: A2, A3, A4 (excludes A1 and proximal vessels) | Acute ischemic stroke due to primary isolated ACA DMVO (A2–A4); treated with mechanical thrombectomy or best medical treatment (±IVT); data from Jan 2013 – Oct 2021 | Yes – excludes A1 occlusions, concomitant large vessel occlusions, DMVOs of other territories, secondary DMVOs, baseline intracerebral hemorrhage | Stent retriever with/without aspiration (first-line in 80/94, 85%), primary aspiration (8/94, 9%), intra‑arterial fibrinolytics (4/94, 4%). General anesthesia in 57%. Median procedure time 39.5 min, median passes 1. | Best medical treatment (BMT) with or without intravenous thrombolysis (IVT) | Not explicitly reported; multiple thrombectomy attempts allowed (median 1–2 passes) | Favorable functional outcome (modified Rankin Scale score 0–2) at 90 days | After PSM: MT 49% (18/37), BMT 49% (19/39); P=0.99 (no significant difference). Unadjusted before PSM: MT 57% (31/54), BMT 39% (18/46); P=0.07. | sICH (Heidelberg/ECASS II): MT 2% (2/94), BMT 2% (1/60); overall 2%. 90‑day mortality after PSM: MT 22% (8/37), BMT 31% (12/39); P=0.36 (not significant). | Successful reperfusion (mTICI 2b‑3): 81% (76/94); complete reperfusion (mTICI 3): 67% (63/94); first‑pass effect: 41% (39/94). Improved reperfusion associated with favorable outcome (OR 4.2, P=0.002). No difference in 24‑hour NIHSS change (−2 vs −1, P=0.52). Procedure‑related complications: iatrogenic dissection/perforation 3%, distal embolization 1%. | CTA, MRA, DSA; noncontrast CT or MRI; perfusion imaging used in some cases | Median onset‑to‑groin puncture (MT): 195 min (Q1–Q3 133–300). Onset‑to‑imaging (BMT): not separately reported. | ROBINS‑I V2: Moderate (retrospective, non‑randomized, selection bias despite PSM, missing outcome data, no core lab for imaging, but propensity matching performed) | Only primary isolated ACA occlusions (A2–A4); excludes A1. MT safe and feasible but no superiority over BMT for 90‑day functional outcome. Successful reperfusion strongly predicted good outcome. Low sICH rate (2%). |
| Meyer L, 2022 | International, retrospective, multicenter, observational registry subanalysis (TOPMOST) | Europe (Germany, Switzerland, France, Spain, etc.), USA, Asia (Singapore); multiple comprehensive stroke centers | Primary isolated distal medium vessel occlusion (DMVO) of the posterior cerebral artery (PCA): P2 segment (from PCA–posterior communicating artery branch point to quadrigeminal cistern) and P3 segment (from quadrigeminal cistern to calcarine fissure) | PCA: P2, P3 (excludes P1, excludes anterior circulation MeVOs) | Acute ischemic stroke due to primary isolated PCA DMVO (P2/P3); treated with endovascular thrombectomy; data from Jan 2014 – Jun 2020 | Yes – excludes PCA P1, basilar, vertebral, M1, ICA; excludes secondary DMVO and proximal large vessel occlusions | First‑pass aspiration only (29%, 41/141) vs first‑pass stent retriever (71%, 100/141). Stent retriever group: with combined aspiration 27%, alone 44%. General anesthesia in 43.2%. Median procedure time 38.5 min. | Not applicable (comparison of aspiration vs stent retriever, no non‑EVT control) | Yes – multiple attempts allowed; rescue strategies: same device (58‑61%), changed device (∼18%), procedure ended (∼10%) | First‑pass effect (FPE) defined as complete reperfusion (mTICI 3) after first thrombectomy attempt | Aspiration: 53.7% (22/41), stent retriever: 44% (44/100); P=0.297 (not significant). Multivariable: FPE associated with shorter procedure time (aOR 0.97 per min, P<0.001), not with technique. | sICH (ECASS II): overall 2.8% (4/141); aspiration 2.4% (1/41), stent retriever 3.0% (3/100). 90‑day mortality: aspiration 13.2% (5/41), stent retriever 10.4% (7/100); P=0.750. | Final mTICI 3: aspiration 75.6%, stent retriever 78% (P=0.758). Early neurological improvement (ENI): aspiration 64.9%, stent retriever 52.2% (P=0.192). 90‑day mRS 0‑1: aspiration 60.5%, stent retriever 68.6% (P=0.40). FPE independently associated with ENI (aOR 3.27, P=0.025). Periprocedural complications: aspiration 4.9%, stent retriever 11% (P not significant). | CTA, MRA, DSA; noncontrast CT (PC‑ASPECTS) | Median onset‑to‑groin puncture: 197 min (IQR 146–275) overall; aspiration 190 min, stent retriever 200 min (P=0.791) | ROBINS‑I V2: Moderate (retrospective, non‑randomized, operator‑dependent technique selection, no core lab for mTICI, missing follow‑up data in stent retriever group, but propensity adjustment not used; comparison only between EVT techniques) | Study compares aspiration vs stent retriever for posterior circulation DMVO (PCA P2/P3), not EVT vs medical management. No control group without EVT. FPE rates similar, safety acceptable. Useful for technical sub‑analysis of MeVO. |
| Meyer L, 2022 | International, retrospective, multicenter, observational registry subanalysis (TOPMOST) | Europe (Germany, Switzerland, France, Spain, etc.), USA, Asia (Singapore); multiple comprehensive stroke centers | Primary isolated distal medium vessel occlusion (DMVO) of the posterior cerebral artery (PCA): P2 segment (from PCA–posterior communicating artery branch point to quadrigeminal cistern) and P3 segment (from quadrigeminal cistern to calcarine fissure) | PCA: P2, P3 (excludes P1, excludes anterior circulation MeVOs) | Acute ischemic stroke due to primary isolated PCA DMVO (P2/P3); treated with endovascular thrombectomy; data from Jan 2014 – Jun 2020 | Yes – excludes PCA P1, basilar, vertebral, M1, ICA; excludes secondary DMVO and proximal large vessel occlusions | First‑pass aspiration only (29%, 41/141) vs first‑pass stent retriever (71%, 100/141). Stent retriever group: with combined aspiration 27%, alone 44%. General anesthesia in 43.2%. Median procedure time 38.5 min. | Not applicable (comparison of aspiration vs stent retriever, no non‑EVT control) | Yes – multiple attempts allowed; rescue strategies: same device (58‑61%), changed device (∼18%), procedure ended (∼10%) | First‑pass effect (FPE) defined as complete reperfusion (mTICI 3) after first thrombectomy attempt | Aspiration: 53.7% (22/41), stent retriever: 44% (44/100); P=0.297 (not significant). Multivariable: FPE associated with shorter procedure time (aOR 0.97 per min, P<0.001), not with technique. | sICH (ECASS II): overall 2.8% (4/141); aspiration 2.4% (1/41), stent retriever 3.0% (3/100). 90‑day mortality: aspiration 13.2% (5/41), stent retriever 10.4% (7/100); P=0.750. | Final mTICI 3: aspiration 75.6%, stent retriever 78% (P=0.758). Early neurological improvement (ENI): aspiration 64.9%, stent retriever 52.2% (P=0.192). 90‑day mRS 0‑1: aspiration 60.5%, stent retriever 68.6% (P=0.40). FPE independently associated with ENI (aOR 3.27, P=0.025). Periprocedural complications: aspiration 4.9%, stent retriever 11% (P not significant). | CTA, MRA, DSA; noncontrast CT (PC‑ASPECTS) | Median onset‑to‑groin puncture: 197 min (IQR 146–275) overall; aspiration 190 min, stent retriever 200 min (P=0.791) | ROBINS‑I V2: Moderate (retrospective, non‑randomized, operator‑dependent technique selection, no core lab for mTICI, missing follow‑up data in stent retriever group, but propensity adjustment not used; comparison only between EVT techniques) | Study compares aspiration vs stent retriever for posterior circulation DMVO (PCA P2/P3), not EVT vs medical management. No control group without EVT. FPE rates similar, safety acceptable. Useful for technical sub‑analysis of MeVO. |
| Mohammaden MH, 2025 | Multicenter retrospective analysis of prospectively collected database; propensity score matched (1:1) intention‑to‑treat analysis | USA and Europe; 14 comprehensive stroke centers | Distal medium vessel occlusion (DMVO) according to Distal Thrombectomy Summit Group consensus, excluding MCA‑M2 (dominant/co‑dominant) and ACA‑A1. Included MCA‑M3/4, ACA‑A2/3, PCA‑P1/P2‑3. | MCA: M3, M4; ACA: A2, A3; PCA: P1, P2, P3 | DMVO as defined; EVT within 24 hours from last known well; data from prospectively collected database | Yes – excludes proximal LVO (ICA, M1, M2 dominant/co‑dominant, A1, basilar, vertebral); excludes multivessel DMVO and proximal LVO with concomitant DMVO | First‑line techniques: stent retriever alone (22.4% GA, 15.1% non‑GA), contact aspiration alone (38.8% vs 35.8%), combined (37.3% vs 37.8%), IA‑tPA (1.5% vs 6.7%), wire maceration (0% vs 4.7%). BGC used less in GA (11.9% vs 26.8%). | Not applicable (comparison of anesthesia modalities: general anesthesia vs non‑general anesthesia [monitored anesthesia care, conscious sedation, local anesthesia]) | Yes – multiple passes allowed; rescue strategies at operator discretion | Shift in degree of disability measured by 90‑day modified Rankin Scale (mRS) ordinal distribution | Matched ITT: common OR 1.19 (95% CI 0.52–2.86), P=0.67 (no significant difference between GA and non‑GA). | sICH: GA 15.5% (9/58), non‑GA 4.5% (3/56); OR 3.24 (0.83–12.68), P=0.09. 90‑day mortality: GA 23.6% (13/55), non‑GA 20% (11/55); OR 1.43 (0.48–4.27), P=0.52. | Secondary outcomes: successful reperfusion (eTICI 2B‑3): GA 90.2% vs non‑GA 78.7%, OR 2.38 (0.80–7.07), P=0.12; 90‑day mRS 0‑2: GA 45.5% vs non‑GA 43.6%, OR 1.14 (0.44–2.96), P=0.79; 90‑day mRS 0‑1: GA 32.7% vs non‑GA 36.4%, OR 0.65 (0.24–1.81), P=0.41. Procedural complications: 4.9% each (OR 1.00). Subgroup analyses no effect modification. As‑treated analysis similar results. | CTA, MRA, DSA; noncontrast CT | Median onset‑to‑puncture (LKW to puncture): GA 330.5 min (IQR 222–661.9), non‑GA 267 min (183.5–511.5) after matching. Procedure time: GA 44 min (32.5–60.5), non‑GA 51 min (33.75–78.5). | ROBINS‑I V2: Moderate (retrospective non‑randomized, selection bias despite PSM, no core lab for occlusion site or reperfusion, variability in DMVO definition and anesthesia protocols, small sample size, but intention‑to‑treat and as‑treated analyses performed) | Study compares anesthesia modalities (GA vs non‑GA), not EVT vs medical management. All patients received EVT. No significant differences in outcomes between GA and non‑GA. Higher sICH rate in GA group but not statistically significant. Matched analysis performed. |
| Mohammaden MH, 2024 (DUSK study) | Multicenter retrospective cohort study with inverse probability of treatment weighting (IPTW) and multivariable regression | 7 comprehensive stroke centers (1 in Europe, 6 in United States) | Distal medium vessel occlusion (DMVO) per Distal Thrombectomy Summit Group consensus, excluding MCA‑M2 (dominant/co‑dominant) and ACA‑A1. Included MCA‑M3/M4, ACA‑A2/A3, PCA‑P1/P2. (PCA‑P3 excluded because all such patients in cohort were treated with MM.) | MCA: M3, M4; ACA: A2, A3; PCA: P1, P2 | Isolated DMVO on initial CTA; pre‑stroke mRS ≤2; LKN to imaging/treatment ≤24 h; 90‑day follow‑up data; treated Jan 2017 – Jun 2021 | Yes – excludes primary proximal LVO (ICA, M1, M2, A1, basilar, vertebral); excludes multivessel occlusion; excludes PCA‑P3 (all in MM); excludes ACA‑A1 due to definition variability | Operator discretion: stent retriever, aspiration, combined, etc. No restriction on device/technique. Successful reperfusion (mTICI ≥2b) in 89.4% (160/179). First‑line techniques not specified in detail. | Medical management (MM) alone, with or without IV thrombolysis (40.8% of MM group received IVT) | Not explicitly reported; at operator discretion | Shift in degree of disability as measured by 90‑day modified Rankin Scale (mRS) ordinal distribution | IPTW analysis: adjusted common OR 1.25 (95% CI 0.95–1.64), P=0.110 (no significant difference). Multivariable analysis: aOR 1.14 (0.74–1.75), P=0.556. | sICH: EVT 5.6% (10/179), MM 6.3% (9/142); multivariable aOR 0.57 (0.21–1.58), P=0.277; IPTW aOR 0.46 (0.24–0.85), P=0.013. 90‑day mortality: EVT 17.3% (31/179), MM 9.2% (13/142); multivariable aOR 1.73 (0.80–3.90), P=0.173; IPTW aOR 1.20 (0.78–1.85), P=0.395. | Good outcome (mRS 0‑2): EVT 53.6%, MM 60.6%, aOR 1.32 (0.97–1.80), P=0.075. Excellent outcome (mRS 0‑1): EVT 36.9%, MM 40.8%, aOR 1.32 (0.94–1.85), P=0.098. Subgroup analysis: EVT associated with higher likelihood of excellent outcome in NIHSS ≥8 (aOR 3.00, 1.69–5.32, Pinteraction=0.001). No other significant subgroup effects. | CTA for occlusion; CT perfusion in subset (40.2% EVT, 46.5% MM); DSA for EVT | Median LKN‑to‑imaging/treatment: EVT 340 min (IQR 200–694), MM 249 min (120–629). (Note: For EVT, puncture time used as surrogate for imaging time.) | ROBINS‑I V2: Moderate (retrospective non‑randomized, selection bias likely, no core lab for occlusion site or reperfusion, imbalanced baseline NIHSS and occlusion sites, but IPTW and multivariable adjustment performed) | Study compares EVT vs MM for isolated non‑M2 DMVO. Overall no significant difference in primary outcome. EVT may benefit patients with higher NIHSS (≥8). Higher mortality in EVT unadjusted but not after adjustment. sICH lower in EVT by IPTW but not by multivariable. Ongoing RCTs awaited. |
| Nguyen TN, 2024 (PLATO study) | International multicenter retrospective case‑control study (30 sites, 8 countries) | 30 sites in Europe (Germany, Switzerland, Finland, France, Spain, Portugal, etc.) and North America (USA, Canada) | Isolated posterior cerebral artery (PCA) occlusion (P1, P2, P3, fetal, or bilateral PCA). Excludes concomitant basilar artery occlusion or multiple vessel occlusion outside PCA territory. | PCA: P1 (53.1%), P2 (40.5%), other (P3, fetal, bilateral – 6.4%) | Isolated PCA occlusion; age ≥18; presentation within 24 h of symptom onset; EVT performed with or without IVT; prestroke mRS 0–3; treated Jan 2015 – Aug 2022 | Yes – excludes basilar artery occlusion, multiple vessel occlusion outside PCA territory, and concomitant large vessel occlusions | First‑line technique: stent retriever (SR) alone (13.2%, n=43), contact aspiration (CA) alone (32.5%, n=106), combined (SR+CA) (54.3%, n=177). General anesthesia more frequent in SR group (72.1%). Median passes: combined group 2, others 1. | Not applicable (comparison of three EVT techniques, no non‑EVT control) | Yes – multiple passes allowed; after first pass, operator could switch technique (data not collected on switching) | First‑pass effect (FPE) – complete reperfusion (eTICI 2c/3) after first thrombectomy pass | FPE: SR 62.8% (27/43), CA 42.5% (45/106), combined 39.6% (70/177). Adjusted: CA vs SR aOR 0.45 (0.19–1.06), P=0.07; combined vs SR aOR 0.35 (0.16–0.80), P=0.01. | sICH (defined per protocol): overall 5.6% (18/326); SR 7.0%, CA 5.8%, combined 5.1% (P=0.892). 90‑day mortality: overall 10.9% (35/321); SR 11.9%, CA 15.4%, combined 8.0% (P=0.156). No significant differences. | Final successful reperfusion (eTICI 2b‑3) 81%, no difference between groups. Functional independence (mRS 0‑2) at 90 days: overall 50.0%; adjusted CA vs SR aOR 0.52 (0.28–0.95), P=0.04; combined vs SR aOR 0.50 (0.24–1.06), P=0.07. Excellent outcome (mRS 0‑1) no difference. FPE associated with higher mRS 0‑2 (58% vs 43.4%, P=0.01). | CTA or MRA for occlusion; CT perfusion in 56%; DSA for EVT; pc‑ASPECTS assessed on noncontrast CT | Median time from last known well to puncture: 3.8 h (IQR 2.4–7.3); 70% within 0–6 h, 30% within 6–24 h | ROBINS‑I V2: Moderate (retrospective, non‑randomized, operator‑dependent technique selection, no core lab for imaging, small SR group, but multivariable adjustment for confounders performed) | Study compares first‑line EVT techniques for isolated PCA occlusion, not EVT vs medical management. SR alone associated with higher FPE and better functional independence vs CA. Safety similar across groups. |
| Nguyen TN, 2024 (PLATO study technique subanalysis) | International multicenter retrospective case‑control study (30 sites, 8 countries) | 30 sites in Europe (Germany, Switzerland, Finland, France, Spain, Portugal, etc.) and North America (USA, Canada) | Isolated posterior cerebral artery (PCA) occlusion (P1, P2, P3, fetal, or bilateral PCA). Excludes concomitant basilar artery occlusion or multiple vessel occlusion outside PCA territory. | PCA: P1 (53.1%), P2 (40.5%), other (P3, fetal, bilateral – 6.4%) | Isolated PCA occlusion; age ≥18; presentation within 24 h of symptom onset; EVT performed with/without IVT; prestroke mRS 0–3; treated Jan 2015 – Aug 2022 | Yes – excludes basilar artery occlusion, multiple vessel occlusion outside PCA territory, and concomitant large vessel occlusions | First‑line technique: stent retriever (SR) alone (13.2%, n=43), contact aspiration (CA) alone (32.5%, n=106), combined (SR+CA) (54.3%, n=177). General anesthesia more frequent in SR group (72.1%). Median passes: combined group 2, others 1. | Not applicable (comparison of three EVT techniques, no non‑EVT control) | Yes – multiple passes allowed; after first pass, operator could switch technique (switching data not collected) | First‑pass effect (FPE) – complete reperfusion (eTICI 2c/3) after first thrombectomy pass | FPE: SR 62.8% (27/43), CA 42.5% (45/106), combined 39.6% (70/177). Adjusted: CA vs SR aOR 0.45 (0.19–1.06), P=0.07; combined vs SR aOR 0.35 (0.16–0.80), P=0.01. | sICH (defined per protocol): overall 5.6% (18/326); SR 7.0%, CA 5.8%, combined 5.1% (P=0.892). 90‑day mortality: overall 10.9% (35/321); SR 11.9%, CA 15.4%, combined 8.0% (P=0.156). No significant differences. | Final successful reperfusion (eTICI 2b‑3) 81%, no difference between groups. Functional independence (mRS 0‑2) at 90 days: overall 50.0%; adjusted CA vs SR aOR 0.52 (0.28–0.95), P=0.04; combined vs SR aOR 0.50 (0.24–1.06), P=0.07. Excellent outcome (mRS 0‑1) no difference. FPE associated with higher mRS 0‑2 (58% vs 43.4%, P=0.01). | CTA or MRA for occlusion; CT perfusion in 56%; DSA for EVT; pc‑ASPECTS assessed on noncontrast CT | Median time from last known well to puncture: 3.8 h (IQR 2.4–7.3); 70% within 0–6 h, 30% within 6–24 h | ROBINS‑I V2: Moderate (retrospective, non‑randomized, operator‑dependent technique selection, no core lab for imaging, small SR group, but multivariable adjustment for confounders performed) | Study compares first‑line EVT techniques for isolated PCA occlusion, not EVT vs medical management. SR alone associated with higher FPE and better functional independence vs CA. Safety similar across groups. |
| Orscelik A, 2024 | Retrospective multicenter registry study (STAR registry) | 32 comprehensive stroke centers (international, mostly US) | Distal medium vessel occlusion (DMVO) per Distal Thrombectomy Summit Group consensus: MCA M2–M4 segments, any segment of ACA, any segment of PCA | MCA: M2, M3, M4; ACA: any segment; PCA: any segment | Adult patients with DMVO undergoing MT; data from 2012–2022; complete clinical and procedural data in STAR Registry | Yes – excludes proximal vessel occlusions (ICA, M1, basilar, vertebral, etc.) | ADAPT, stent retriever, Solumbra (combined), other. BGC used in 231 patients (15.3%), non‑BGC in 1277 (84.7%). Femoral access most common (99.1% in BGC group). | Not applicable (comparison of BGC vs non‑BGC, no non‑EVT control) | Yes – multiple passes allowed; rescue techniques at operator discretion | Favorable functional outcome (90‑day modified Rankin Scale score 0–2) | BGC 38.6% (73/189), non‑BGC 45.2% (458/1013); unadjusted P=0.11; multivariate aOR 0.89 (0.59–1.35), P=0.60 | sICH (ECASS II PH2): BGC 4.2% (9/214), non‑BGC 5.1% (60/1176); P=0.68; multivariate aOR 0.73 (0.30–1.54), P=0.40. 90‑day mortality not directly reported; discharge mortality: BGC 8.9% (18/202), non‑BGC 13.5% (137/1013); multivariate aOR 0.47 (0.23–0.88), P=0.03. | Technical: BGC associated with lower mTICI ≥2C (43.2% vs 52.7%, P=0.01), longer puncture‑to‑intracranial access (15 vs 8 min, P<0.01), longer puncture‑to‑recanalization (97 vs 34 min, P<0.01). Distal embolization lower in BGC (8.8% vs 14.9%, P=0.03). In Solumbra subgroup: lower FPE (17.4% vs 30.7%, P=0.03). Procedure time differences diminished over years. | CTA, CT perfusion, DWI, DSA | Median onset‑to‑puncture not given; procedure times: puncture‑to‑intracranial access (BGC 15 min, non‑BGC 8 min); puncture‑to‑final recanalization (BGC 97 min, non‑BGC 34 min) | ROBINS‑I V2: Moderate (retrospective, non‑randomized, selection bias due to operator choice of BGC, no core lab adjudication, but large multicenter registry with multivariate adjustment) | Study compares BGC vs non‑BGC for DMVO, not EVT vs medical management. BGC associated with lower recanalization quality and longer procedure times but reduced distal embolization. No significant difference in 90‑day functional outcome or sICH. |
| Palmisano V, 2025 | Retrospective review of a prospective multicenter database (5 Italian centers) | Italy, 5 stroke centers | Any occlusion at or distal to the M2 segment of MCA and A1 segment of ACA (anterior circulation distal medium vessel occlusions) | M2, M2/M3, M3, M4, A2 (majority M2, 89.9%) | AIS due to anterior circulation DMVO; treated with MT using JET D reperfusion catheter (0.054 inch inner lumen) as first-line approach; Jan 2020 – Dec 2021 | Yes – excludes isolated proximal arterial occlusions (e.g., ICA, M1, A1? Actually A1 was excluded as per definition “at or distal to M2 and A1” – but A1 itself not included; patients with proximal occlusion alone were excluded); also excludes posterior circulation strokes, age <18, mRS ≥3, incomplete data | First-line: direct aspiration (DA) alone vs combined technique (CT = aspiration + stent retriever). All cases used the same JET D reperfusion catheter (outer diameter 1.65 mm, distal inner lumen 0.054 inches). Stent retrievers used: EmboTrap II, Solitaire X, Trevo NXT. Guiding catheter in proximal ICA. | Not applicable (comparison of two EVT techniques: DA vs CT, no non‑EVT control) | Yes – second-line strategies with adjunctive devices at physician discretion (15.8% DA, 18.4% CT) | First‑pass complete recanalization (mTICI 3 after single pass) and 90‑day modified Rankin Scale score 0‑2 (functional independence) | First‑pass mTICI 3: DA 40.0% vs CT 10.5% (P<0.001); 90‑day mRS 0‑2: DA 63.7% vs CT 36.1% (P=0.001). Multivariable: DA independent predictor of first‑pass mTICI 3 (aOR 0.33, 0.12‑0.95, P=0.04). | sICH not separately defined; overall hemorrhagic complications: DA 7.4% vs CT 18.4% (P=0.035); parenchymal hematoma (potential sICH surrogate): DA 4.2% vs CT 10.5% (P=0.136). 90‑day mortality: DA 9.9% vs CT 27.8% (P=0.004). | Final mTICI 2b‑3: DA 89.5% vs CT 71.1% (P=0.003); final mTICI 3: 58.9% vs 28.9% (P<0.001). Shorter puncture‑to‑reperfusion time (65±43 vs 101±60 min, P<0.001). Fewer passes (1.52±0.93 vs 2.10±1.30, P=0.001). Predictors of good 90‑day outcome: lower age, final mTICI 2b‑3 (aOR 10.82), final mTICI 3 (aOR 2.80). | CTA, DSA; ASPECTS on noncontrast CT | Mean puncture‑to‑reperfusion time: DA 65±43 min, CT 101±60 min (P<0.001). Onset‑to‑puncture not reported. | Study compares DA vs CT for anterior DMVO using same dedicated distal aspiration catheter (JET D). DA showed superior first‑pass complete reperfusion, final reperfusion, shorter procedure time, better 90‑day functional outcome, lower mortality, and fewer hemorrhagic complications. Majority of occlusions were M2 (90%). | Study compares DA vs CT for anterior DMVO using same dedicated distal aspiration catheter (JET D). DA showed superior first‑pass complete reperfusion, final reperfusion, shorter procedure time, better 90‑day functional outcome, lower mortality, and fewer hemorrhagic complications. Majority of occlusions were M2 (90%). |
| Pérez‑García C, 2020 | Single‑center retrospective analysis of a prospectively maintained database | Spain, single comprehensive stroke center | Medium vessel occlusions (MeVOs) defined as occlusions located in mid/distal M2, M3 of MCA; A1‑A3 of ACA; P1 and distal PCA. Associated with significant clinical deficit (NIHSS ≥5 or ≤5 with disabling deficit). | MCA: mid M2, distal M2, M3; ACA: A1, A2, A3; PCA: P1, distal PCA | Patients with acute ischemic stroke undergoing MT for MeVO (as defined) between Feb 2017 – Jan 2020 | Yes – excludes basilar artery occlusion, distal ICA or M1 occlusions, proximal M2 occlusions (by definition) | BEMP technique: mini stent retriever (Catch Mini 3×15/3×20 mm or Aperio 3.5×28 mm) + low‑profile DAC (Arc Mini or 3MAX, 0.035″ inner diameter) via blind exchange and mini‑pinning, with balloon guide catheter (Flowgate2) proximal flow arrest. Control group: mini stent retriever alone (same devices without DAC). | Not applicable (comparison of two EVT techniques: BEMP vs mini stent retriever alone, no non‑EVT control) | Yes – rescue therapy with other devices/techniques after failure of frontline approach (22% in mini SR, 7.1% in BEMP) | MeVO first‑pass recanalization – expanded Thrombolysis in Cerebral Ischemia (eTICI) 2c/3 after single pass | BEMP 57% (32/56), mini SR 34% (17/50); P=0.017. Multivariable: BEMP independently associated (OR 2.72, 1.19–6.22, P=0.018). | sICH (Heidelberg classification): BEMP 1.9% (1/52, excluding rescue cases), mini SR 12.8% (5/39); P=0.038. 90‑day mortality: BEMP 20.8% (11/53), mini SR 22.4% (11/49); P=0.835 (not significant). | End‑of‑procedure eTICI 2c/3: BEMP 66% vs 46% (P=0.037). Need for rescue therapy lower with BEMP (7.1% vs 22%, P=0.028). Emboli to new territory lower with BEMP (1.8% vs 12%, P=0.035). No difference in 90‑day functional independence (mRS 0‑2): BEMP 50.9% vs mini SR 53.1% (P=0.831). Subarachnoid hemorrhage or parenchymal hematoma rates similar. | CTA for occlusion, DSA for thrombectomy, noncontrast CT and cone‑beam CT for hemorrhagic complications | Mean onset‑to‑puncture: BEMP 4.3±3.3 h, mini SR 4.5±2.2 h (P=0.215). Mean puncture‑to‑recanalization: BEMP 44.9±21.6 min, mini SR 55.4±30.9 min (P=0.077) | ROBINS‑I V2: Moderate (single‑center, retrospective, non‑randomized, temporal bias – early period used mini SR alone, later period used BEMP; however, prospective database, blinded angiogram review, and multivariable adjustment performed) | Study compares BEMP technique vs mini stent retriever alone for MeVOs, not EVT vs medical management. BEMP associated with higher first‑pass complete recanalization and lower sICH, but no difference in 90‑day functional outcome. sICH rate calculated after excluding rescue cases. |
| Palmisano V, 2025 | Retrospective review of a prospective multicenter database (5 Italian centers) | Italy, 5 stroke centers | Any occlusion at or distal to the M2 segment of MCA and A1 segment of ACA (anterior circulation distal medium vessel occlusions) | M2, M2/M3, M3, M4, A2 (majority M2, 89.9%) | AIS due to anterior circulation DMVO; treated with MT using JET D reperfusion catheter (0.054″ inner lumen) as first-line approach; Jan 2020 – Dec 2021 | Yes – excludes isolated proximal arterial occlusions (e.g., ICA, M1, A1? Actually A1 itself not included); posterior circulation strokes; age <18; pre‑stroke mRS ≥3; incomplete data | First-line: direct aspiration (DA) alone vs combined technique (CT = aspiration + stent retriever). All cases used JET D catheter (outer 1.65 mm, inner 0.054 inches). Stent retrievers: EmboTrap II, Solitaire X, Trevo NXT. Guiding catheter in proximal ICA. | Not applicable (comparison of two EVT techniques: DA vs CT, no non‑EVT control) | Yes – second-line strategies with adjunctive devices at physician discretion (DA 15.8%, CT 18.4%) | First‑pass complete recanalization (mTICI 3 after single pass) and 90‑day functional independence (mRS 0‑2) | First‑pass mTICI 3: DA 40.0% vs CT 10.5% (P<0.001); 90‑day mRS 0‑2: DA 63.7% vs CT 36.1% (P=0.001). Multivariable: DA independent predictor of first‑pass mTICI 3 (aOR 0.33, 0.12‑0.95, P=0.04). | First‑pass mTICI 3: DA 40.0% vs CT 10.5% (P<0.001); 90‑day mRS 0‑2: DA 63.7% vs CT 36.1% (P=0.001). Multivariable: DA independent predictor of first‑pass mTICI 3 (aOR 0.33, 0.12‑0.95, P=0.04). | Final mTICI 2b‑3: DA 89.5% vs CT 71.1% (P=0.003); final mTICI 3: 58.9% vs 28.9% (P<0.001). Shorter puncture‑to‑reperfusion time (65±43 vs 101±60 min, P<0.001). Fewer passes (1.52±0.93 vs 2.10±1.30, P=0.001). Predictors of good 90‑day outcome: lower age, final mTICI 2b‑3 (aOR 10.82), final mTICI 3 (aOR 2.80). | CTA, DSA; ASPECTS on noncontrast CT | Mean puncture‑to‑reperfusion time: DA 65±43 min, CT 101±60 min (P<0.001). Onset‑to‑puncture not reported. | ROBINS‑I V2: Moderate (retrospective, non‑randomized, technique choice based on center preference (3 centers favored DA, 2 favored CT), no core lab adjudication, but independent blinded angiogram evaluation and multivariate adjustment performed) | Study compares DA vs CT for anterior DMVO using same dedicated distal aspiration catheter (JET D). DA superior in first‑pass complete reperfusion, final reperfusion, shorter procedure time, better 90‑day functional outcome, lower mortality, and fewer hemorrhagic complications. Majority M2 occlusions (90%). sICH not separately reported; use parenchymal hematoma as proxy with caution. |
| Rizzo F, 2024 | Prospective single‑center observational study | Italy, single comprehensive stroke center (Bologna) | Medium vessel occlusion: M3 or distal M2 segment of MCA, A2 or A3 of ACA, P2 or P3 of PCA (per Saver et al., Stroke 2020) | Distal M2, M3; A2, A3; P2, P3 | Consecutive patients with AIS due to primary MeVO (as defined) treated with IVT, EVT alone, or BT between Jan 2019 – Jul 2021 | Yes – excludes proximal LVO (ICA, M1, basilar, vertebral), and secondary MeVOs (from clot migration) | EVT performed with stent retriever, aspiration, or combined technique per operator discretion. No restriction on devices. BT group received IVT (alteplase) followed by EVT. EVT‑only group received EVT alone (IVT ineligible or contraindicated). | Not a controlled trial; comparisons between IVT alone, EVT alone, and BT. No non‑treatment control. | Yes – at operator discretion; not detailed. | Good functional outcome defined as modified Rankin Scale (mRS) score 0–2 at 3 months | BT: 63.9% (53/83); EVT‑only: 31.6% (12/38); IVT‑only: 61% (36/59). Multivariable analysis: BT independently associated with mRS 0‑2 (OR 2.76, 95% CI 1.08–7.07) compared to EVT‑only. | sICH (SITS‑MOST): overall 1.1% (2/180); EVT‑only 2.6% (1/38), BT 0% (0/83), IVT‑only 1.7% (1/59). 90‑day mortality: overall 2.2% (4/180); EVT‑only 2.6% (1/38), BT 1.2% (1/83), IVT‑only 3.4% (2/59). No significant differences. | Successful recanalization (mTICI ≥2b): BT 74.4% (58/78), EVT‑only 56.4% (22/39) (p=0.049). Mean NIHSS at discharge: BT 4.4±5.2, EVT‑only 8.7±7.6 (p<0.001). Procedural complications: vasospasm 4.1% (5/121), SAH 1.7% (2/121), no neurological deterioration. | Noncontrast CT, multiphase CTA, CT perfusion (all patients), DSA for EVT; ColorViz used in subset. | Median onset‑to‑imaging: IVT 123 min, EVT‑only 203 min, BT 128 min (p=ns). Median stroke‑to‑needle (IVT): 164 min (IVT), 161.5 min (BT). Median onset‑to‑groin: EVT‑only 250 min, BT 225 min (p=ns). | ROBINS‑I V2: Moderate (prospective observational, non‑randomized, significant baseline imbalances: EVT‑only had higher NIHSS, lower ASPECTS, older age? Actually IVT group older, EVT‑only higher NIHSS. Selection bias for treatment allocation. However, multivariable adjustment performed.) | Study compares three reperfusion strategies, not just EVT vs medical management. BT associated with better outcomes than EVT‑only, likely due to selection bias (EVT‑only had worse baseline characteristics). Low sICH rates overall. Prospective design, single center. |
| Rosi A, 2024 | Retrospective before‑after cohort study (single center) | Switzerland, single comprehensive stroke center (Geneva University Hospitals) | MCA M2 segment occlusion (distal to M1, including proximal and distal M2). Reference to Ospel & Goyal, J Neurointerv Surg 2021 for MeVO definition. | MCA M2 segment (primary, secondary, or concomitant M2 occlusions) | AIS due to M2 occlusion treated with MTB; two 12‑month periods: before (May 2017–Jun 2018, control) and after (Jul 2018–Aug 2019, 3D‑RA group) introduction of 3D‑RA guidance; NIHSS ≥6 or <6 with disabling deficit | Yes – excludes isolated ICA or M1 occlusions (though patients with proximal occlusion could have secondary M2 occlusion after proximal recanalization) | Combined technique (stent retriever + distal aspiration) in majority (86% control, 91.2% 3D‑RA group); aspiration alone in remainder. 3D‑RA group: 3D‑RA acquisition (3.9 min) followed by selection of optimal 2D working projections (4.8 min) to guide MTB. Control group: standard AP, LL, and oblique DSA projections. | Not a control group in RCT sense; historical cohort treated without 3D‑RA guidance (standard DSA projections only) | Yes – multiple passes allowed; median 2 passes in both groups (IQR 1–4) | Reperfusion rate: mTICI ≥2b for overall MCA territory (primary endpoint in study); 90‑day mRS 0‑2 (secondary) | mTICI ≥2b: 3D‑RA group 100% (57/57), control 74.4% (32/43), P<0.001. 90‑day mRS 0‑2: 3D‑RA 49.1% (26/53), control 45% (18/40), P=0.834 (no significant difference). | sICH (defined as post‑procedural hemorrhage): 3D‑RA group 5.3% (3/57), control 2.3% (1/43), P=0.697. 90‑day mortality: not explicitly reported; mRS 6 included in mRS 3‑6 category (overall 50.9% in 3D‑RA, 55% in control). | 3D‑RA improved mTICI shift (93% improved vs 65.1%, P=0.001) and MeVO‑M‑TICI 2c‑3 rate (54.4% vs 34.9%, P<0.001). No difference in overall procedure time (48.7 vs 52.3 min, P=0.596). Additional time for 3D‑RA acquisition and projection selection was ~8.7 min. No increase in emboli to new territory (1.8% vs 2.3%). | Noncontrast CT, CTA, CTP (RAPID), DSA, 3D‑RA (Allura Clarity FD20) | Onset‑to‑treatment window: up to 8 h (or 8‑24 h with perfusion selection). Mean time from first M2 visualization to final recanalization: 3D‑RA 48.7±29.9 min, control 52.3±36.5 min (P=0.596). | ROBINS‑I V2: Moderate (retrospective before‑after design, potential temporal bias, non‑randomized, small sample, but consecutive patient inclusion and blinded outcome assessment?) | Study compares imaging guidance technique (3D‑RA vs standard DSA) for M2 occlusions, not EVT vs medical management. 3D‑RA improves recanalization rates without increasing procedural time, but does not significantly improve 90‑day functional outcome. General anesthesia used in all but 4 control patients. |
| Saber H, 2022 | Multicenter retrospective cohort study with inverse probability of treatment weighting (IPTW) | USA, 11 stroke centers (7 contributed MM data, 11 contributed EVT data) | Distal medium vessel occlusion (DMVO) in anterior circulation: M3 segment of MCA (defined from circular sulcus of insula to superior surface of Sylvian fissure) or any segment of ACA (A1, A2, A3) – excludes M2, posterior circulation | MCA M3, ACA (any segment: A1, A2, A3) | AIS with causative anterior circulation DMVO (M3 or ACA) within 24 h of last known well; no proximal LVO at presentation; pre‑stroke mRS 0–1; data from Jan 2015 – Dec 2019 | Yes – excludes concomitant proximal LVO (ICA, M1, etc.), and patients with proximal occlusion at presentation | Aspiration alone (49.0%), stent retriever with/without aspiration (43.5%), intra‑arterial alteplase (7.5%). First‑pass recanalization (mTICI 2b‑3) in 56.6%. Successful reperfusion (mTICI 2b‑3) in 81.2%. General anesthesia in 15.1%. | Aspiration alone (49.0%), stent retriever with/without aspiration (43.5%), intra‑arterial alteplase (7.5%). First‑pass recanalization (mTICI 2b‑3) in 56.6%. Successful reperfusion (mTICI 2b‑3) in 81.2%. General anesthesia in 15.1%. | Yes – multiple passes allowed; rescue strategies at operator discretion | 3‑month functional independence (modified Rankin Scale score 0–2) | IPTW‑adjusted: EVT 51.7% vs MM 44.0%; aOR 1.36 (95% CI 0.84–2.19), P=0.20 (not significant). | sICH (≥4 NIHSS increase + parenchymal hematoma): EVT 4.0% (weighted), MM 3.1% (weighted); P=0.90. 90‑day mortality: EVT 18.7% (weighted), MM 15.7% (weighted); aOR 1.24 (0.63–2.43), P=0.53. | Excellent outcome (mRS 0‑1): EVT 38.4% vs MM 26.7%; aOR 1.71 (1.02–2.87), P=0.04 (significant). In subgroup baseline NIHSS >5, EVT associated with higher excellent outcome (aOR 1.86, P=0.05). ACA vs M3 outcomes: ACA had lower functional independence (40.4% vs 56.7%, P=0.06) and more passes (2.1 vs 1.5, P=0.03). | CTA or MRA for occlusion; CT or MR perfusion as per standard protocol; DSA for EVT | Mean last‑known‑well to ED transfer: EVT 266.8±207.6 min, MM 241.9±202.9 min (P=0.40). All within 24 h. | ROBINS‑I V2: Moderate (retrospective, non‑randomized, selection bias – EVT group had higher NIHSS; IPTW used but residual confounding possible; MM data from only 7 of 11 centers) | Excludes M2 occlusions; focuses on M3 MCA and ACA. EVT did not significantly improve functional independence but improved excellent outcome (mRS 0‑1). Higher mortality in EVT not significant. Larger RCTs needed. |
| Salim HA, 2026 | Multicenter retrospective propensity‑matched cohort study (37 centers) | 37 academic centers across North America, Asia, Europe (MAD‑MT registry) | Distal medium vessel occlusion (DMVO) of MCA: M2, M3, M4 segments (per Saver et al., Stroke 2020) | MCA M2, M3, M4 | AIS due to MCA DMVO (M2–M4); treated with IAT or MT (with/without IVT); data from Sep 2017 – Jul 2023 | Yes – excludes proximal LVO (ICA, M1, etc.), and strokes due to large artery atherosclerosis, vasculitis, malignancy, SLE | IAT: intra‑arterial urokinase (250,000‑600,000 units over ≤2h). MT: aspiration, stent retriever, or combined (operator discretion). Both ± IVT. | Not applicable (comparison of IAT vs MT, both are EVT; no non‑EVT control) | Yes – multiple passes allowed; rescue strategies at operator discretion | Excellent functional outcome: 90‑day modified Rankin Scale score 0‑1 | After PSM: IAT 38% (7/18), MT 21% (34/160); P=0.20 (not significant) | sICH (Heidelberg classification): MT 6.7% (10/150), IAT 0% (0/18); P=0.60. 90‑day mortality: IAT 13% (2/16), MT 17% (27/158); P>0.99. | Functional independence (mRS 0‑2): IAT 50% (9/18), MT 40% (64/160); P=0.42. Successful reperfusion (mTICI 2b‑3): MT 90% vs IAT 68%, P=0.02. Puncture‑to‑recanalization time longer with IAT (55 vs 35 min, P=0.002). No significant differences in any ICH (39% vs 27%, P=0.31) or other complications. | CTA, DSA; follow‑up CT/MRI; no core lab adjudication | Median onset‑to‑puncture: IAT 261 min (IQR 152‑372), MT 227 min (IQR 170‑323); P=0.94. Puncture‑to‑recanalization: IAT 55 min (43‑64), MT 35 min (23‑57); P=0.002. | ROBINS‑I V2: Moderate (retrospective, non‑randomized, small IAT sample size (n=19) after matching, selection bias possible, no core lab, but propensity matching used) | Study compares IAT vs MT for MCA DMVO, not EVT vs medical management. IAT showed numerically better functional outcomes and fewer safety events, but differences not significant. Very small IAT group limits conclusions. |
| Salim HA, 2024 | Multicenter retrospective propensity‑matched cohort study (37 centers) | 37 academic centers across North America, Asia, Europe (MAD‑MT registry) | Distal medium vessel occlusion (DMVO) as defined by Saver et al. (Stroke 2020): includes medium proximal (M2, A1, P1) and medium distal vessels (M3/M4, A2/A3, P2/P3) | M2, M3, M4; A1, A2, A3; P1, P2, P3 | AIS due to DMVO; complete 90‑day mRS data; treated with either MT‑IVT or IVT alone; data from Sep 2017 – Jul 2023 | Yes – excludes large vessel occlusions (ICA, M1, basilar, etc.); also excludes patients treated with MT alone or no reperfusion therapy | MT‑IVT group: first‑line techniques – aspiration 23%, combined 64%, stent retriever 13%. Median passes 1 (IQR 1‑3). Successful reperfusion (mTICI 2b‑3) 91%, complete (2c‑3) 61%. General anesthesia in 34%. | IVT alone (intravenous thrombolysis without mechanical thrombectomy) | Not applicable for IVT alone group; in MT‑IVT group, multiple passes and rescue strategies at operator discretion | Functional independence: 90‑day modified Rankin Scale (mRS) score 0‑2 | After PSM: MT‑IVT 62% (197/320), IVT alone 63% (202/320); adjusted OR 1.00 (95% CI 0.66‑1.51), P>0.99 | sICH (Heidelberg): MT‑IVT 8.3% (26/320), IVT alone 6.9% (22/320); adjusted OR 0.78 (0.42‑1.45), P=0.44. 90‑day mortality: MT‑IVT 14% (44/320), IVT alone 11% (36/320); adjusted OR 0.75 (0.44‑1.29), P=0.30. | Any ICH significantly higher in MT‑IVT (32% vs 17%, aOR 0.43, P<0.001), driven by HI1 and SAH. Excellent outcome (mRS 0‑1): MT‑IVT 45%, IVT alone 50% (P=0.21). Subgroup analysis: “drip‑and‑ship” scenario had higher ICH risk with MT‑IVT (P=0.018). No difference by occlusion site (medium vs distal) except ICH in medium occlusions. | CTA, DSA; follow‑up CT/MRI; no core lab | Median onset‑to‑IVT needle: MT‑IVT 155 min, IVT alone 164 min (P=0.31). MT‑IVT: onset‑to‑puncture 227 min, puncture‑to‑recanalization 39 min, onset‑to‑recanalization 283 min. | ROBINS‑I V2: Moderate (retrospective, non‑randomized, selection bias possible despite PSM; missing data imputed; no core lab; but large sample, multinational) | Study compares MT‑IVT vs IVT alone, not EVT vs medical management. Both groups received IVT. MT‑IVT did not improve functional outcomes but increased any ICH, though sICH unchanged. Subgroup analysis suggests possible harm in “drip‑and‑ship” patients. |
| Salim HA, 2025 | Multicenter retrospective propensity‑matched cohort study (MAD‑MT registry)v | 37 academic centers across North America, Asia, Europe | Distal medium vessel occlusion (DMVO) of MCA (M2, M3, M4 segments) as defined by Saver et al. (Stroke 2020). Excludes ACA and PCA MeVOs. | MCA M2, M3, M4 (M2 = medium, M3/M4 = distal) | AIS due to MCA DMVO; treated with EVT or BMM (with/without IVT); data from Sep 2017 – Jul 2023 | Yes – excludes proximal LVO (ICA, M1, etc.); excludes intra‑arterial thrombolysis; excludes pre‑stroke mRS 5 | First‑line: aspiration 20%, combined 69%, stent retriever 11%. Successful reperfusion (mTICI 2b‑3) 90%, complete (2c‑3) 58%. Median passes 1. IVT given in 74% of EVT group. | Best medical management (BMM): includes IVT if eligible, antiplatelet, risk factor management, secondary prevention; no EVT | Yes – multiple passes allowed; rescue strategies at operator discretion | Functional independence: 90‑day modified Rankin Scale (mRS) score 0‑2 (one of 10 co‑primary endpoints) | After PSM: EVT 59% (187/391), BMM 59% (206/391); adjusted OR 1.00 (95% CI 0.67–1.50), P>0.99 | sICH (Heidelberg): EVT 8.4% (31/391), BMM 3.0% (11/391); adjusted OR 3.56 (1.69–7.48), P<0.001. 90‑day mortality: EVT 13% (42/391), BMM 13% (46/391); adjusted OR 1.48 (0.87–2.51), P=0.15. | Any ICH: EVT 37% vs BMM 19% (aOR 2.61, P<0.001). Subarachnoid hemorrhage more frequent in EVT (9.0% vs 1.7%, aOR 6.71). Subgroup analysis: EVT not beneficial for distal occlusions (M3/M4) for mRS 0‑2 (P for interaction=0.005). No significant difference in excellent outcome (mRS 0‑1): EVT 43% vs BMM 47% (aOR 0.81, P=0.26). | CTA, DSA; follow‑up CT/MRI; no core lab adjudication | Median onset‑to‑puncture: 250 min (IQR 172–359); puncture‑to‑recanalization: 38 min (26–58); onset‑to‑recanalization: 308 min (228–427). All within 24 h. | ROBINS‑I V2: Moderate (retrospective, non‑randomized, selection bias possible despite PSM; no core lab; residual confounding; but large multicenter sample, propensity matching with multiple imputation) | Study only includes MCA DMVO (M2–M4); excludes ACA/PCA. EVT did not improve functional outcomes but significantly increased sICH and any ICH compared with BMM. Subgroup suggests possible harm in more distal occlusions (M3/M4). |
| Salsano G, 2024 | Retrospective two‑center cohort study with inverse probability of treatment weighting (IPTW) | Italy, 2 comprehensive stroke centers | Primary distal medium vessel occlusion (DMVO) as defined by Saver et al. (Stroke 2020): includes distal or nondominant M2, M3, M4 of MCA; A2, A3 of ACA; P2, P3 of PCA | Distal/nondominant M2, M3, M4; A2, A3; P2, P3 | Consecutive AIS with primary DMVO on initial CTA; treated with IVT alone or EVT±IVT within 16 h of last known well; 3‑month mRS available; 2019‑2023 | Yes – excludes LVO (ICA, M1, dominant M2), secondary DMVO, tandem lesions | First‑line: contact aspiration 62%, stent retriever (± aspiration) 38%. Successful recanalization (TICI ≥2b) 90%, complete (TICI 3) 46%. Median passes 1 (IQR 1‑2). Procedure time mean 47 min. | IVT alone (intravenous thrombolysis without EVT) – all 64 patients in control group received IVT | Yes – multiple passes allowed; no specific details on rescue strategies | Excellent outcome (90‑day mRS 0‑1) and functional independence (90‑day mRS 0‑2) – both primary endpoints (co‑primary) | IPTW‑adjusted: mRS 0‑1 – aOR 1.58 (0.71‑3.51), P=0.27; mRS 0‑2 – aOR 2.02 (0.85‑4.85), P=0.12 (neither significant) | sICH (ECASS‑2): EVT 6.0% (3/50), IVT 9.4% (6/64); aOR 0.49 (0.10‑2.39), P=0.38. 90‑day mortality: EVT 16.0% (8/50), IVT 20.3% (13/64); aOR 0.50 (0.18‑1.41), P=0.19. | Early neurological improvement (day 1 NIHSS decrease) crude rate higher in EVT (48% vs 26.6%, P=0.03) but not after adjustment (aOR 2.22, P=0.073). SAH: EVT 8% vs IVT 12.5% (aOR 0.56). Percentage of cerebral volume lost: IVT 41.7% vs EVT 26.6% (P=0.187, adjusted estimate −19.17, P=0.104). Multivariable predictors of excellent outcome: lower baseline NIHSS (OR 0.72) and early neurological improvement (OR 20.25). | CTA for occlusion; CT perfusion (RAPID) for at‑risk volume; DSA for EVT; follow‑up CT for final infarct volume | Mean onset‑to‑IVT: EVT 161 min, IVT 109 min (P=0.003). Mean onset‑to‑groin puncture: 243 min; onset‑to‑recanalization: 288 min; procedure time: 47 min. All within 16 h. | ROBINS‑I V2: Moderate (retrospective, non‑randomized, selection bias – EVT group had higher baseline NIHSS and more anticoagulants; IPTW used but residual confounding possible; small sample size) | Both groups received IVT in EVT±IVT group (68% of EVT patients actually received IVT). The control group received IVT alone (no EVT). Primary outcomes showed no significant differences. EVT did not increase sICH or mortality. |
| Siegler JE, 2025 | International multicenter retrospective propensity‑matched cohort study (MAD‑MT registry) | 37 centers across 11 countries (Austria, Belgium, Canada, France, Germany, Japan, Italy, Portugal, Singapore, Taiwan, USA) | Distal or medium vessel occlusion (DMVO) as per Saver et al. (Stroke 2020): includes medium/proximal (M2, P1, A1) and medium/distal (M3, P2, A2 or more distal) | M2, M3; A1, A2; P1, P2 (and more distal) | Adults with DMVO undergoing EVT; device type (Trevo vs other) reported on first pass; complete pre‑stroke and 90‑day mRS data | Yes – excludes proximal LVO (ICA, M1, basilar, etc.) | First‑line: Trevo (with aspiration in 88.8%) vs other devices (including other stent retrievers and aspiration). Contact aspiration used in 82.4% of other device group. Combined technique common. | Not applicable (comparison of two EVT device strategies, no non‑EVT control) | Yes – multiple passes allowed; median passes: Trevo 1 (IQR 1‑2), other 2 (IQR 1‑3) | Favorable shift in 90‑day modified Rankin Scale (mRS) score (ordinal shift analysis) | Adjusted proportional OR 1.10 (95% CI 0.80‑1.51), P=0.56 (no significant difference). Median mRS: Trevo 2 (IQR 1‑4), other 3 (IQR 1‑4). | sICH not explicitly reported; parenchymal hematoma grade 1‑2: Trevo 9.5% (16/169), other 7.9% (14/178) (P=0.60). 90‑day mortality: Trevo 19.9% (52/261), other 20.7% (54/261); adjusted OR 0.93 (0.57‑1.51), P=0.76. | Trevo associated with fewer passes (adjusted β -0.25, P=0.03), higher odds of successful recanalization (TICI 2b‑3, aOR 1.97, P=0.02), greater 24‑h NIHSS improvement (adjusted β -1.74, P=0.01). No difference in first‑pass effect (aOR 1.14, P=0.60). Procedural complications: Trevo 8.5%, other 9.2% (P=0.91). | CTA, DSA; ASPECTS on noncontrast CT | Median last‑known‑well to puncture: Trevo 263 min (IQR 175‑392), other 260 min (180‑399). Median onset‑to‑recanalization: Trevo 303 min (220‑467), other 315 min (224‑469). | ROBINS‑I V2: Moderate (retrospective, non‑randomized, selection bias possible despite PSM; no core lab; missing data; but large multicenter cohort, propensity matching with sensitivity analyses) | Study compares Trevo vs other EVT devices for DMVO, not EVT vs medical management. Trevo associated with better technical efficacy (fewer passes, higher successful recanalization) and early neurological improvement, but no difference in 90‑day functional outcome or mortality. Safety profile similar. |
| Siegler JE, 2023 | International multicenter retrospective propensity‑matched cohort study | 37 centers across 10 countries (Austria, Belgium, Canada, France, Germany, Japan, Italy, Portugal, Singapore, Taiwan, USA) | Based on Saver et al. (Stroke 2020): proximal medium vessel occlusion (MeVO) = M2, A1, P1; distal medium vessel occlusion = M3, A2, P2, or further | M2, M3; A1, A2; P1, P2; and more distal branches | Patients with acute ischemic stroke due to medium or distal vessel occlusion (as defined) treated with endovascular thrombectomy using first‑line aspiration alone or stent‑retriever alone (no combined technique); complete key clinical data (age, pre‑mRS, past medical history, baseline NIHSS, ASPECTS, first‑line technique, flow control, device info) | Yes – excludes proximal large vessel occlusions (e.g., ICA, M1, basilar). Also excludes patients treated with combined technique (aspiration + stent‑retriever) on first pass. | First‑line: aspiration alone (contact aspiration catheter) vs stent‑retriever alone (no combined). Procedures performed at operator discretion. Successful recanalization (eTICI 2b‑3) achieved in 90.2% (aspiration) vs 82.7% (stent‑retriever) in unadjusted analysis. | Not applicable (comparison of two EVT techniques, no non‑EVT control) | Yes – multiple passes allowed; rescue strategies at operator discretion (data on number of passes reported) | Successful recanalization – final expanded Thrombolysis in Cerebral Infarction (eTICI) score 2b‑3 | Unadjusted: aspiration 90.2% (220/244), stent‑retriever 82.7% (162/196); OR 0.85 (0.74‑0.98), P=0.02. After multivariable adjustment: aOR 0.88 (0.72‑1.09), P=0.24 (not significant). In PS‑matched cohort (n=105 each): adjusted OR 0.94 (0.75‑1.18), P=0.60. | sICH not separately reported; any ICH (any type) in primary cohort: aspiration 25.3% (61/241), stent‑retriever 34.2% (66/193); adjusted OR 1.10 (0.96‑1.26), P=0.19. Parenchymal hematoma (PH1/2): aspiration 8.2% (16/195), stent‑retriever 9.3% (13/140); aOR 1.15 (0.85‑1.56), P=0.35. 90‑day mortality: aspiration 18.6% (42/226), stent‑retriever 18.5% (36/195); adjusted OR 0.96 (0.81‑1.13), P=0.62. | No significant difference in first‑pass effect (58% vs 55%, P=0.59), final eTICI 2c‑3, number of passes, procedural complications, 24‑hour NIHSS change, or 90‑day mRS shift. Distal occlusions (vs proximal) were independently associated with higher odds of excellent reperfusion (eTICI 2c‑3, aOR 2.97), greater NIHSS improvement (β 4.12), and favorable mRS shift (proportional OR 1.72). No significant interaction between technique and occlusion location. | CTA, DSA; ASPECTS on noncontrast CT | Median last‑known‑well to puncture: aspiration 278 min (IQR 190‑497), stent‑retriever 252 min (163‑399). Median last‑known‑well to recanalization: aspiration 328 min (226‑532), stent‑retriever 314 min (211‑464). | ROBINS‑I V2: Moderate (retrospective, non‑randomized, selection bias possible; residual confounders despite PS matching; no core lab; missing data; but large multicenter cohort, propensity matching and multivariable adjustment performed) | Study compares aspiration vs stent‑retriever alone for medium/distal occlusions, not EVT vs medical management. Primary outcome was technical (recanalization), not clinical. No significant differences in clinical outcomes. Combined techniques (aspiration + stent‑retriever) were excluded. |
| Zhang K, 2026 | Multicenter retrospective registry with propensity score matching (PSM) and inverse probability of treatment weighting (IPTW) | China, 25 centers | Anterior cerebral artery (ACA) occlusion (A1, A2, A3 segments) — considered a medium vessel occlusion (MeVO) | ACA A1, A2, A3 | Acute ischemic stroke due to ACAo confirmed by CTA/MRA/DSA; prestroke mRS ≤2; time from onset to treatment within 24 h; treated with EVT or SMM exclusively (no bridging? Actually EVT could be with or without IVT, SMM includes IVT if eligible) | Yes – excludes primary proximal large vessel occlusion (ICA, M1, etc.) with subsequent clot migration to ACA; excludes multiterritory occlusion | First‑line: aspiration alone 28.7%, combined aspiration + stent retriever 60.7%, stent retriever alone 10.7%. General anesthesia in 64.6%. Successful recanalization (mTICI 2b‑3) in 85.5%. | Standard medical management (SMM) alone: includes IVT if eligible (23.3% in SMM group), antiplatelet, risk factor management; no EVT | Yes – multiple passes allowed; details not specified | 90‑day modified Rankin Scale (mRS) score distribution (ordinal shift) | After PSM: adjusted OR 2.33 (95% CI 1.45–3.77), P<0.001 favoring EVT. After IPTW: adjusted OR 2.14 (1.59–2.89), P<0.001. | sICH (ECASS II): no significant difference between groups (PSM aRR 1.12, P=0.793; IPTW aRR 1.01, P=0.976). 90‑day mortality: PSM aRR 0.42 (0.14–1.30), P=0.133; IPTW aRR 0.62 (0.30–1.28), P=0.195. | Secondary outcomes: excellent outcome (mRS 0‑1) after IPTW: EVT 42.7% vs SMM 34.7%, aRR 1.77 (1.27–2.48); functional independence (mRS 0‑2): 62.7% vs 52.8%, aRR 2.35 (1.63–3.39). Subgroup: EVT beneficial in NIHSS ≥6 (OR 1.96), cardioembolic (OR 1.62), other causes (OR 6.18); not beneficial in large‑artery atherosclerosis (OR 0.88). Interaction by NIHSS and etiology significant. | CTA, MRA, DSA; CT perfusion (Fast‑Processing of Ischemic Stroke software) used in subset for mismatch | Mean onset‑to‑admission: SMM 391±207 min, EVT 392±243 min. Within 24 h. | ROBINS‑I V2: Moderate (retrospective, non‑randomized, selection bias possible despite PSM and IPTW; missing data imputed; no core lab; but large multicenter, sensitivity analyses performed) | Study focuses on isolated ACA occlusion (A1‑A3). EVT associated with better functional outcomes compared to SMM, without increased sICH or mortality. Benefits most pronounced in patients with NIHSS ≥6 and non‑atherosclerotic etiologies. Generalizability limited to Chinese population with lower IVT use. |
| Nedelcu S, 2023 | Single‑center retrospective cohort study | USA, single center (UMass Memorial Medical Center) | Based on Saver et al. (Stroke 2020): occlusion of M2‑4, A2‑3, P1‑2, PICA, AICA, SCA | M2, M3, M4; A2, A3; P1, P2; PICA, AICA, SCA | DMVO (as defined); NIHSS ≤6; ASPECTS >6; presentation within 24 h from last seen well; Jan 2018 – Dec 2021 | Yes – excludes LVO (ICA, M1, basilar) | Operator discretion: aspiration, stent retriever, or combined. Successful recanalization (TICI 2b‑3) in 87.2% (34/39). First‑line technique distribution not specified. | Best medical management (BMT): includes IVT if eligible (14.6% of BMT group), antiplatelet/anticoagulation, risk factor management | Yes – multiple passes allowed; details not specified | 90‑day good functional outcome (mRS 0‑2) and excellent outcome (mRS 0‑1) | Good outcome: EVT 69.2% (27/39), BMT 70.7% (29/41); OR 0.56 (0.17‑1.83), P=0.341. Excellent outcome: EVT 48.7% (19/39), BMT 56.1% (23/41); OR 0.45 (0.15‑1.34), P=0.152. | sICH not specifically reported; parenchymal hemorrhage (PH1+PH2): EVT 12.8% (5/39), BMT 9.8% (4/41); OR 0.9 (0.22‑3.62), P=0.879. 90‑day mortality: EVT 10.3% (4/39), BMT 4.9% (2/41); P=0.426. | No difference in NIHSS at discharge, ΔNIHSS, discharge mRS, or clinical deterioration. Subgroup with penumbra on CTP (29 patients) showed EVT associated with greater NIHSS improvement (P=0.047). No difference in any ICH (38.5% vs 24.2%, P=0.229). | CTA, CT perfusion (subset), DSA for EVT | Median onset‑to‑groin puncture (EVT): 455 min (IQR 231‑780). All within 24 h. | ROBINS‑I V2: Moderate (single‑center, retrospective, small sample, selection bias possible, but adjusted analyses performed) | Study focuses on mild strokes (NIHSS ≤6). EVT did not improve outcomes vs BMT. Limited statistical power. Subgroup analysis suggested potential benefit in patients with penumbra on CTP. |
| Shimonaga K, 2025 | Single‑center retrospective cohort study | Japan, single center (National Cerebral and Cardiovascular Center, Osaka) | M2 segment occlusion of middle cerebral artery | M2 segment (proximal/distal M2, no further specification) | M2 occlusion; MT attempted; data from Jan 2014 – Jul 2022 | Yes – excludes ATBI requiring angioplasty/stenting; excludes M1, ICA occlusions | SR alone (41%), CA alone (31%), CT (SR+aspiration, 28%). Balloon‑guided catheters used when possible. Local anesthesia with conscious sedation. | Not applicable (comparison of three EVT techniques, no non‑EVT control) | Yes – multiple passes allowed; rescue therapy in 16% (16/100) | Procedural outcomes (passes, time, recanalization, FPE, mFPE) and clinical outcome (90‑day mRS 0‑2) | 90‑day mRS 0‑2: 50.8% (68/134) overall; no significant difference between techniques. Subgroup by M1‑M2 angle: obtuse angle 45.3%, acute angle 55.7% (P=0.23). | sICH (NIHSS increase ≥4): overall 2.2% (3/134). SAH: 28.4% (38/134). Any ICH: 48.5% (65/134). Mortality not reported. | In obtuse angle group (n=64): CA associated with lower SAH (9% vs SR 39% vs CT 50%, P=0.02). In acute angle group (n=70): CT superior in passes (1.4 vs 1.8 vs 2.4, P=0.03), puncture‑recanalization time (48.5 vs 59.1 vs 69.4 min, P=0.04), and mFPE (67% vs 48% vs 21%, P=0.01). No technique difference in 90‑day mRS 0‑2. | DSA (biplanar), follow‑up CT/MRI | Mean puncture‑recanalization time: 61.5±3.2 min overall; acute angle 57.8 min, obtuse angle 65.5 min. Onset‑to‑puncture not reported. | ROBINS‑I V2: Moderate (single‑center, retrospective, small sample, operator‑dependent technique selection, no core lab, but consecutive patient inclusion) | Study compares three EVT techniques for M2 occlusion, not EVT vs medical management. M1‑M2 bifurcation angle influences technique outcomes: CA safer for obtuse angles (less SAH), CT more effective for acute angles (better mFPE). No difference in 90‑day functional outcome. |
| Hu D, 2026 | Single‑center retrospective cohort study with inverse probability weighting (IPW) | China, single center (Nanjing Pukou People’s Hospital) | Occlusion of M2‑M4, A1‑A3, P1‑P3 (proximal MeVO: M2, A1, P1; distal MeVO: M3‑M4, A2‑A3, P2‑P3) | M2, M3, M4; A1, A2, A3; P1, P2, P3 | AIS due to MeVO; IVT‑ineligible; presentation within 24 h; Jan 2021 – Dec 2024; prestroke mRS <2 | Yes – excludes concomitant LVO (ICA, M1, basilar); excludes IVT recipients; excludes prestroke mRS ≥2 | SR (Solitaire 4×20mm, EmboTrap II 3/4×22mm), aspiration (SOFIA 5F, 3MAX/4MAX, ACE60), or combined. Local anesthesia or conscious sedation preferred. | Conservative treatment (standard medical management without IVT): antiplatelet/anticoagulant, neuroprotective agents; no EVT, no IVT | Yes – intra‑arterial fibrinolytics or tirofiban used selectively after several failed attempts | Favorable functional outcome: 90‑day modified Rankin Scale (mRS) score 0‑1 | IPTW‑adjusted: EVT 54.6% vs conservative 51.0% (P=0.675); mRS 0‑2: 68.0% vs 67.6% (P=0.998). No significant difference. | sICH (Heidelberg): EVT 8.2% (8/97 weighted), conservative 0% (0/145 weighted), P=0.002. 90‑day mortality: EVT 6.2% vs conservative 8.3% (P=0.721). | Recanalization (complete or partial): EVT 74.2% vs conservative 27.6% (P<0.001). Subgroup analysis: EVT associated with favorable outcome only in NIHSS ≥6 (aOR 1.83, 1.17‑2.76, P=0.012). No interaction for age, occlusion location, atrial fibrillation, onset‑to‑door time, core volume, or Tmax volume. | CTA for occlusion; NCCT for HT; follow‑up CTA/MRA at 24‑72h; DSA for EVT | Median onset‑to‑admission: 521 min (IQR 386‑624); within 24 h | ROBINS‑I V2: Moderate (single‑center, retrospective, small EVT sample, selection bias possible despite IPTW; no core lab; but IPTW used to balance covariates) | Study focuses on IVT‑ineligible MeVO patients only – a distinct population where EVT is the sole reperfusion strategy. EVT did not improve overall functional outcomes but increased sICH. Subgroup suggests potential benefit in NIHSS ≥6. |
| Alawieh AM, 2023 | International multicenter retrospective registry study (STAR) | 32 centers (international, mostly USA) | DVO defined as M3/M4 segments of MCA, A2/A3 of ACA, P2/P3 of PCA (isolated distal occlusions, no proximal occlusion) | MCA M3/M4, ACA A2/A3, PCA P2/P3 | Consecutive patients undergoing EVT for DVO (as defined); data from STAR registry | Yes – excludes proximal LVO (ICA, M1, basilar) and excludes rescue thrombectomy for distal embolization from proximal occlusion | Frontline: ADAPT (aspiration), stent retriever (SR), or primary combined (PC). Operator preference. | Not applicable (comparison of EVT techniques, no non‑EVT control) | Yes – at operator discretion | 90‑day good functional outcome (modified Rankin Scale score 0‑2) | DVO overall: 44% (unadjusted). Propensity‑matched DVO cohort: ADAPT 47%, SR/PC 47% (no significant difference) | sICH (PH2 or NIHSS drop ≥4): overall DVO ~5‑6%; matched: ADAPT 3%, SR/PC 9% (P>0.05). 90‑day mortality not separately reported for DVO; overall 90‑day mortality for DVO ~? Not given. | Successful recanalization (mTICI 2b‑3) in DVO: 75%. Independent predictors of good outcome: younger age, lower admission NIHSS, IV‑tPA use, successful recanalization, procedure time ≤60 min (aOR 17.8), ≤2 attempts (aOR 2.34). Procedure time ≤60 min predicted good outcome in ADAPT but not SR/PC. ≤2 attempts predicted good outcome in SR/PC but not ADAPT. | CTA, DSA; follow‑up CT/MRI for hemorrhage | Mean onset‑to‑groin (matched): ADAPT 441±318 min, SR/PC 336±418 min. Procedure time: ADAPT 42±34 min, SR/PC 49±46 min. | ROBINS‑I V2: Moderate (retrospective, operator‑dependent technique selection, no core lab, propensity matching used) | Study compares frontline techniques for DVO, not EVT vs medical management. DVO outcomes comparable to LVO. Technical futility metrics: aspiration more sensitive to procedure time, stent retriever more sensitive to number of attempts. |
| Elhorany M, 2022 | Single‑center retrospective cohort study with propensity score matching (1:1) | France, single center (Pitié‑Salpêtrière Hospital, Paris) | Distal vessel occlusions: distal aspect of M2 (above mid‑height of insula), M3, any ACA segment, any PCA segment. Proximal M2 excluded. | M2 distal, M3; A1, A2, A3; P1, P2, P3 | Primary isolated distal vessel occlusion (as defined); treated with MT+BMT (2015‑2020) vs BMT alone (2006‑2015) | Yes – excludes proximal LVO (ICA, M1, proximal M2); excludes secondary DVOs from MT complication | Stent retriever (≤3 mm) with/without aspiration (75%), or 3MAX aspiration alone (25%). Conscious sedation in 84.1% (37/44), general anesthesia in 15.9% (7/44). | Best medical treatment (BMT) alone: includes IVT if eligible (41.7% of BMT group), antiplatelet, etc. No EVT. | Not explicitly reported | Good functional outcome: 90‑day modified Rankin Scale (mRS) score 0‑2 | Before PSM: MT+BMT 47.7% (21/44), BMT 58.3% (21/36); P=0.38. After PSM (n=28 each): MT+BMT 53.6% (15/28), BMT 57.1% (16/28); OR 0.87 (0.3‑2.4), P=1.00. | sICH (ECASS II): before PSM MT+BMT 4.5% (2/44), BMT 0% (0/36), P=0.5; after PSM MT+BMT 3.6% (1/28), BMT 0% (0/28), P=1.00. 90‑day mortality: before PSM 18.2% vs 13.9% (P=0.76); after PSM 7.1% (2/28) vs 10.7% (3/28), OR 0.64 (0.1‑4), P=1.00. | Successful recanalization (AOL ≥2 or mTICI ≥2b): MT+BMT 90.9% (40/44) vs BMT 47.2% (17/36) (P<0.001). Early neurological improvement (ENI): MT+BMT 45.5% vs BMT 38.9% (P=0.65). Excellent outcome (mRS 0‑1): after PSM 32.1% vs 39.3% (P=0.78). Procedural complications: distal embolization 4.5% (2/44), vessel perforation 4.5% (2/44). | MRI (DWI, TOF MRA) or CT; DSA for EVT; follow‑up CT/MRI at 24h | Median onset‑to‑door: MT+BMT 119 min (IQR 82‑199), BMT 164 min (119‑233); P=0.11 | ROBINS‑I V2: Moderate (single‑center, retrospective, PSM limited to 3 covariates, selection bias likely, but adjusted analysis performed) | Study compares MT+BMT vs BMT alone for distal vessel occlusions (excluding proximal M2). No significant difference in functional outcomes or mortality. sICH low but only in EVT group. Small sample size after matching. |
| Nagel S, 2020 | Single‑center retrospective cohort study (Heidelberg Recanalization Registry) | Germany, single center (University Hospital Heidelberg) | Distal arterial occlusions: distal to M1 segment of MCA (M2, M3), anterior cerebral artery (A2), or distal to top of basilar artery (P1, P2) | M2 (64.1% in IVT, 93.3% in EVT), M3 (15.6% vs 3.3%), A2 (3.1% vs 0%), P1 (3.1% vs 3%), P2 (14.1% vs 0%) | NIHSS ≤5; distal vessel occlusion (as defined); treated with IVT alone or EVT (±IVT); data up to Apr 2019 | Yes – excludes M1, ICA, basilar top (included? Actually top of basilar was included as "distal", but M1 excluded) | Stent retriever (90%), aspiration (10%). General anesthesia or conscious sedation. Successful recanalization (TICI 2b‑3) in 86.6%. | IVT alone (intravenous thrombolysis, with rescue EVT in 3 patients after neurological deterioration) | Yes – 3 patients (4.6%) in IVT group received rescue EVT after early neurological worsening | Excellent outcome (90‑day mRS 0‑1 or return to baseline) and good outcome (90‑day mRS 0‑2 or return to baseline) | Excellent outcome: EVT 70% (21/30), IVT 75% (48/64), P=0.65. Good outcome: EVT 73.3% (22/30), IVT 87.5% (56/64), P=0.14. | sICH (HBC class 2/PH2): EVT 13.3% (4/30), IVT 1.6% (1/64), P=0.01. 90‑day mortality: EVT 13.3% (4/30), IVT 1.6% (1/64), P=0.04. | EVT group had more M2 occlusions (93.3% vs 64.1%, P=0.02). Door‑to‑needle time shorter in EVT (38 vs 67 min). Door‑to‑vessel time shorter in EVT (1.75 vs 7.0 h). Multivariate analysis: only age predicted excellent outcome (OR 0.95 per year). | CTA, MRA, DSA; CT perfusion or MRI perfusion used in subset | Mean LSW‑to‑treatment: IVT 3.62 h, EVT 3.73 h. Door‑to‑needle: IVT 67 min, EVT 38 min (P=0.03). | ROBINS‑I V2: Moderate (single‑center, retrospective, small sample, selection bias – EVT group had more M2 occlusions, but multivariable adjustment performed) | Study compares EVT vs IVT alone for mild stroke (NIHSS ≤5) with distal occlusions. EVT was associated with higher sICH and mortality, despite similar functional outcomes. Very small EVT sample (n=30). |
| Mehta A, 2025 | Single‑center retrospective cohort study | USA, single center (Mount Sinai, New York) | DMVO: M2, M3, M4; A1, A2; P1, P2 (per Gibo et al. for MCA, standard definitions for ACA/PCA) | M2 (80.6%), M3 (5.8%), M4 (NR); A1/A2 (4.8%); P1/P2 (6.8%) | AIS due to DMVO (as defined); EVT performed; Dec 2014 – Jul 2023 | Yes – excludes ICA terminus, basilar, M1 | Aspiration alone (72%), combined aspiration + stent retriever (24%), other (4%). Operator discretion. | Not applicable (comparison of anesthesia modalities: CS vs GA) | Yes – multiple passes allowed; rescue strategies at operator discretion | Early neurological improvement (ENI: NIHSS decrease ≥8 at 24h), successful reperfusion (TICI 2b‑3), first‑pass effect, 90‑day good outcome (mRS 0‑3) | ENI: CS 20.6% vs GA 17.7% (P=0.73). Successful reperfusion: CS 79.8% vs GA 72.9% (P=0.33). 90‑d mRS 0‑3: CS 41.7% vs GA 34.4% (P=0.12). | ICH (PH1/2): CS 2.2% (5/193), GA 4.2% (4/69) (P=0.51). SAH: CS 10.96%, GA 10.42% (P=1.0). 90‑d mortality: CS 10.96% (25/193), GA 19.79% (19/69) (P=0.04 unadjusted; adjusted OR 0.51, P=0.16). | Multivariable analysis: CS associated with higher odds of ENI (aOR 2.59, P<0.05) and successful reperfusion (aOR 2.33, P<0.05). No difference in first‑pass effect, ICH, SAH, or 90‑day mRS 0‑3. Room‑to‑puncture time longer in GA (28 vs 22 min, P=0.01). | CTA, DSA | Median LKW‑to‑groin puncture: CS 292 min, GA 282.5 min. Puncture‑to‑recanalization: CS 38 min, GA 39 min (P=0.48). | ROBINS‑I V2: Moderate (single‑center, retrospective, selection bias – GA group had higher baseline NIHSS, but multivariable adjustment performed) | Study compares anesthesia modalities (CS vs GA) for DMVO, not EVT vs medical management or EVT techniques. Good outcome defined as mRS 0‑3. CS associated with better ENI and successful reperfusion in adjusted analysis. |
| Valente I, 2024 | Multicenter retrospective propensity‑matched cohort (16 centers) | Italy, 16 comprehensive stroke centers | Isolated M2 occlusion (genu or proximal branch of MCA) | M2 (superior, middle, inferior branches) | NIHSS ≤5; isolated M2 occlusion; immediate MT (iMT) – no prior best medical management; data Jan 2016 – Dec 2021 | Yes – excludes M1, ICA, basilar, and other LVO | Stent retriever ± aspiration, or direct aspiration. General anesthesia or conscious sedation/local anesthesia at operator discretion. | Not applicable (comparison of anesthesia modalities: GA vs CS/LA) | Yes – multiple passes allowed; details not specified | 90‑day excellent neurologic outcome: modified Rankin Scale (mRS) score 0‑1 | After PSM: GA 74.5% (35/47), CS/LA 72.3% (34/47); adjusted OR 1.12 (0.45‑2.79), P=0.815 | sICH not reported; symptomatic SAH (sSAH): GA 2.2% (1/47), CS/LA 13.5% (5/47), P=0.082. 90‑day mortality: GA 8.5% (4/47), CS/LA 4.3% (2/47), adjusted OR 2.09 (0.36‑12.02), P=0.408. | Secondary outcome mRS 0‑2: GA 87.2%, CS/LA 80.8% (P=0.401). Successful recanalization (mTICI 2b‑3): GA 89.4%, CS/LA 83.0% (P=0.374). No difference in NIHSS at 72h, intraprocedural dissection, or new territory embolism. | CTA, DSA | Median onset‑to‑groin: GA 345 min, CS/LA 272 min (after matching P=0.849). Groin‑to‑reperfusion: GA 40 min, CS/LA 38 min (P=0.349). | ROBINS‑I V2: Moderate (retrospective, non‑randomized, selection bias possible despite PSM; good outcome definition not standard 0‑2; but multicenter, blinded image review) | Study compares anesthesia modalities (GA vs CS/LA) for minor stroke (NIHSS ≤5) with isolated M2 occlusion undergoing iMT. No difference in 90‑day clinical outcomes. Not suitable for EVT vs medical management analysis. |
| Hofmeister J, 2025 | Single‑center retrospective cohort study | Switzerland, single center (Geneva University Hospitals) | M2 segment occlusion (medium vessel occlusion) | M2 segment only | Primary MeVO (M2) treated with MT; intraoperative 3D imaging (CE‑CBCT or 3DRA) at start of procedure; Oct 2022 – Sep 2023 | Yes – excludes M1, ICA, basilar | Combined technique (stent retriever + aspiration catheter) in all patients. Balloon‑guided catheter used. General anesthesia. | Not applicable (comparison of two imaging guidance modalities: CE‑CBCT vs 3DRA; same EVT technique) | Yes – multiple passes allowed | Visualization of proximal and distal clot edges and distal arteries on intraoperative imaging (DSA, 3DRA, CE‑CBCT) | Distal clot edge visualized: CE‑CBCT 60.0% vs 3DRA 2.7% (P<0.001). Distal arteries visualized: CE‑CBCT 74.3% vs 3DRA 8.1% (P<0.001). | sICH not separately reported; dissection 0%, perforation 0%, embolization to new territory 2.7‑2.8%. 90‑day mortality not reported. | CE‑CBCT associated with higher first‑pass effect (65.7% vs 37.8%, P=0.02), fewer passes (1.69 vs 2.23, P=0.043), higher final eTICI 3 (71.4% vs 43.2%, P=0.024). No difference in procedure time or clinical outcome (mRS). | DSA, 3DRA, CE‑CBCT (Vaso‑CT) | Mean first DSA to recanalization: CE‑CBCT 38.8 min, 3DRA 39.6 min (P=0.869) | ROBINS‑I V2: Moderate (single‑center, retrospective, small sample, non‑randomized, but consecutive patients) | Study compares imaging guidance techniques, not EVT vs medical management or EVT technique types. CE‑CBCT improves visualization and technical outcomes but not 90‑day mRS. |
| Berberich A, 2025 | International multicenter retrospective cohort study with IPTW (PLATO registry) | 31 sites across 9 countries (Europe, North America, Asia) | Isolated PCA occlusion: P1, P2, P3, or fetal PCA segments | P1 (56.7%), P2 (40.2%), P3 (3.2%), fetal PCA (included with P1) | Isolated PCA occlusion; EVT performed; presentation within 24 h; prestroke mRS 0–3; Jan 2015 – Aug 2022 | Yes – excludes basilar artery occlusion, multiple vessel occlusion, and other LVO (M1, ICA, etc.) | First-line: stent retriever 12.9%, contact aspiration 31.4%, combined 54.3%, IA lytic 1.4%. General anesthesia in GA group, local/conscious sedation in non-GA. | Not applicable (comparison of anesthesia modalities: GA vs non-GA, both receive EVT) | Yes – multiple passes allowed; median 1 (IQR 1–2) | 90-day ordinal shift in modified Rankin Scale (mRS) score distribution | IPTW: common OR 0.89 (95% CI 0.53–1.51), P=0.67 (no significant difference) | sICH (Heidelberg): non-GA 7.8% (15/193), GA 5.0% (9/179); IPTW OR 0.54 (0.17–1.76), P=0.31. 90-day mortality: non-GA 10.9% (21/193), GA 11.6% (21/181); IPTW OR 1.18 (0.60–2.33), P=0.64. | GA associated with higher successful reperfusion (eTICI 2b‑3: 83.6% vs 73.6%, aOR 1.70, P=0.005). No difference in functional independence (mRS 0‑2: 50.0% vs 47.7%), excellent outcome (mRS 0‑1: 30.8% vs 29.4%), or early neurological improvement. Subgroup analysis showed age interaction (P=0.017). | CTA, MRA, DSA; CT perfusion in 57% | Median time to treatment: 3.7 h (IQR 2.3–7.2); 70% within 0–6 h, 30% within 6–24 h | ROBINS‑I V2: Moderate (retrospective, non‑randomized, selection bias – GA group had higher NIHSS and lower pc‑ASPECTS; IPTW used but residual confounding possible) | Study compares anesthesia strategies (GA vs non-GA) for isolated PCA occlusion, not EVT vs medical management. No difference in functional outcomes despite higher reperfusion with GA. |
| Psychogios M (DISTAL), 2025 | Phase 3, multicenter, prospective, randomized, open-label, blinded-outcome (PROBE) trial | 55 hospitals in 11 countries (mainly Europe) | Occlusion of nondominant or codominant M2, M3/M4; A1/A2/A3; P1/P2/P3 (dominant M2 excluded) | M2 (nondominant/codominant) 44.0%, M3 26.9%, M4 0.2%, A1 0.2%, A2 2.9%, A3 1.8%, P1 5.5%, P2 13.4%, P3 3.1% | Age ≥18; isolated MeVO (as defined); NIHSS ≥4 (or lower if disabling); presentation within 24 h of last known well; prestroke home living | Yes – excludes dominant M2, M1, ICA, basilar | First-line: combined (64.6%), stent retriever only (15.7%), aspiration only (15.7%). Successful reperfusion (mTICI 2b‑3) in 71.7%. Median puncture‑to‑recanalization 70 min. | Best medical treatment (BMT) alone – includes IVT if eligible (65.4% of BMT group) | Yes – multiple passes allowed | Level of disability measured by ordinal shift in 90‑day modified Rankin Scale (mRS) score | Unadjusted common OR 0.90 (95% CI 0.67‑1.22), P=0.50 (no significant difference) | sICH (modified SITS‑MOST): EVT 5.9% (16/271), BMT 2.6% (7/272); OR 2.38 (0.44‑6.14). 90‑day mortality: EVT 15.5% (42/271), BMT 14.0% (38/272); OR 1.17 (0.71‑1.90). | Excellent outcome (mRS 0‑1): EVT 34.7% vs BMT 37.5% (OR 0.88). Median NIHSS change at 24h: 0.4 vs 0.3. No difference in quality of life or cognitive function. Subgroup analyses showed no benefit across all prespecified subgroups (NIHSS, age, time, occlusion site, IVT use). | CTA or MRA for occlusion; DSA for EVT; core lab adjudication | Median LKW‑to‑randomization: EVT 3.8 h, BMT 4.0 h. Median LKW‑to‑arterial puncture (EVT): 4.9 h. Median imaging‑to‑puncture: 70 min. | RoB 2: Low – randomized, blinded outcome assessment, independent core lab, intention‑to‑treat, minimal missing data (0 lost to follow‑up, 3 withdrawals) | Neutral RCT for MeVO EVT. Lower successful reperfusion rate (71.7%) than in LVO trials. Higher sICH in EVT group but not significant. Results support no routine EVT for nondominant/codominant M2 and more distal occlusions. |
