# Supplementary material for: Evidence, strategies, and pathways for thrombectomy in medium vessel occlusion stroke: a systematic review
Source: Front Neurol. 2026 Sep 29;17:1838008. doi: 10.3389/fneur.2026.1838008 (PMC13658856; doi:10.3389/fneur.2026.1838008)
Supplement: Supplementary file 9 [file Table_2.docx]

Supplementary Table S2. Detailed inclusion and exclusion criteria for study selection.

| Standard Category | Inclusion Criteria | Exclusion (Title/Abstract Screening, n = 339) | Exclusion (Full-Text Screening, n = 4) |
| --- | --- | --- | --- |
| Research Types | •Randomized Controlled Trial (RCT)  •Prospective or Retrospective Observational Study (Cohort Study, Case-Control Study) | • Narrative Review (0)  •Descriptive Study (14)  •Case Report /Case Series (41)  •Technical Video or Technical Note (3)  •Animal Experiment, In Vitro Study  •Ongoing or Not Yet Commenced Study (13)  •Expert Discussion / Commentary (0)  •Abstract with Unavailable Full Text or Incomplete Data  •Review/commentary/opinion (72) | Review/Commentary/Opinion (1) |
| Study Subjects | •Adult patients (≥18 years old)  •Diagnosed with Acute Ischemic Stroke (AIS)  •Radiologically confirmed Medium Vessel Occlusion (MeVO) | •Non-adult patients (< 18 years old) (3)  •Medium or small vessel occlusions located outside the brain (e.g., cardiac, aortic arch)  •Large Vessel Occlusion (LVO) or stroke with unspecified occlusion site(3)  •Asymptomatic or with minor non-disabling neurological deficits (low NIHSS score and absence of disabling symptoms)  •Coexisting other intracranial pathologies (e.g., aneurysm, arterial dissection, arteriovenous malformation)(2) |  |
| Interventions | •Endovascular Mechanical Thrombectomy: Served as the primary or comparative intervention.  •Use of any type of thrombectomy device is permitted. | •Patients who received only intravenous thrombolysis (IVT) or conservative management without thrombectomy (if the study focus is a pure device comparison).(3)  •No thrombectomy (3)  •Studies where endovascular mechanical thrombectomy was not the primary recanalization strategy (0). |  |
| Publication Criteria | •Published in peer-reviewed academic journals  •Clear methodology description and complete data  •Published in reputable journals in the fields of neurointervention, stroke, or related disciplines | •Published in non-peer-reviewed journals or predatory journals  •Conference abstracts (without a full published paper)(82) | Published in non-peer-reviewed journals or predatory journals(1); Conference Abstracts (2) |
| Relevance | Study focus consistent with review objectives | Not relevant (100) |  |
