# Supplementary material for: Evidence, strategies, and pathways for thrombectomy in medium vessel occlusion stroke: a systematic review
Source: Front Neurol. 2026 Sep 29;17:1838008. doi: 10.3389/fneur.2026.1838008 (PMC13658856; doi:10.3389/fneur.2026.1838008)
Supplement: Supplementary file 10 [file Table_3.docx]

Supplementary Table S3. Excluded studies with reasons

(Legend: The following studies underwent full-text review but were subsequently excluded based on predefined scientific criteria. Studies excluded during the initial title/abstract screening phase are not listed individually; their numbers and reasons are summarized in the PRISMA flow diagram (Figure 1). None of the exclusion decisions were based on journal ranking, indexing status, or other non-methodological factors.)

Abbreviations: M2/M3, segments of the middle cerebral artery; AVM, arteriovenous malformation; CTP, CT perfusion; STAR, Systematic Thrombectomy Aspiration Registry; DMVO, distal medium vessel occlusion.

| First Author (Year) | Title | Specific Reason for Exclusion |
| --- | --- | --- |
| Hao et al., 2026 | Anterior vs Posterior Circulation Medium-vessel Occlusions and Response to Endovascular Thrombectomy- a Post-hoc Analysis of the ESCAPE MeVO Trial | Conference abstract only; no full text available |
| Guo et al., 2025 | Intravenous Thrombolysis before Mechanical Thrombectomy in Patients with Acute Ischemic Stroke Due to Medium Vessel Occlusion | Review article; not original research |
| Fourcand et al., 2023 | Clinical Outcomes Of Endovascular Thrombectomy Versus Acute Medical Treatment In Acute Ischemic Strokes Secondary To Medium Vessel Occlusion | Conference abstract only; no full text available |
| Farè et al., 2025 | Mechanical thrombectomy vs intravenous rt-PA in medium vessel occlusions: real-world data from the Italian SITS registry | Preprints that had not undergone peer review were excluded. |
