# Supplementary material for: Evidence, strategies, and pathways for thrombectomy in medium vessel occlusion stroke: a systematic review
Source: Front Neurol. 2026 Sep 29;17:1838008. doi: 10.3389/fneur.2026.1838008 (PMC13658856; doi:10.3389/fneur.2026.1838008)
Supplement: Supplementary file 11 [file Table_4.docx]

Supplementary Table S4. Structured imaging report template for MeVO stroke.

Abbreviations: rCBF, relative cerebral blood flow; Tmax, time to maximum; EVT, endovascular thrombectomy.

| Section | Item | Description/Example Entry |
| --- | --- | --- |
| Part 1: Patient & Exam Info | Patient ID | e.g., P001 |
|  | Symptoms & Onset Time | e.g., acute left-sided weakness and aphasia for 2 hours. 2025-10-09 10:00 |
|  | Clinical NIHSS Score | e.g., 7 (if available)  • 0-1: Normal  • 1-4: Minor Stroke  • 5-15: Moderate Stroke  •15-20: Moderate-Severe Stroke  • 21-42: Severe Stroke |
|  | Examination Date/Time | e.g., 2025-10-10 10:00 (One-Stop CT Protocol) |
| Part 2: Imaging Findings | Non-Contrast CT | |
|  | Early Ischemic Changes | e.g., blurring of the gray-white matter junction and sulcal effacement in the right temporal and posterior parietal cortex. |
|  | ASPECTS Score | e.g., 7 (Points deducted: M5, M6) |
|  | Exclusion of Hemorrhage | e.g., no hyperdense hemorrhage identified. |
|  | CT Angiography | |
|  | Site of Occlusion | e.g., right middle cerebral artery, M2 segment (superior division). |
|  | Collateral Circulation (e.g., Modified Tan Score) | e.g., Grade 2 (collateral filling >50% but <100%), suggesting moderate collateral compensation ^[83]^. |
|  | Other Vascular Findings | e.g., no significant stenosis, occlusion, or vascular anomaly in the cervical ICA or vertebrobasilar system. |
|  | CT Perfusion | |
|  | Software Used | e.g., syngo Via |
|  | Ischemic Core (rCBF < 30%) | • Volume: 12 mL  • Location: e.g., insular cortex, lentiform nucleus, M3 region. |
|  | Hypoperfused Area (Tmax > 6.0 s) | • Volume: 45 mL |
|  | Ischemic Penumbra | • Volume: 33 mL (calculated as Hypoperfused Volume [45 mL] - Core Volume [12 mL]) ^[84]^.  • Mismatch Ratio: 2.75 (calculated as Penumbra Volume [33 mL] / Core Volume [12 mL]). |
| Part 3: Diagnosis & Conclusion | Comprehensive Diagnosis | e.g., acute occlusion of the right M2 segment (MCA), ASPECTS 7. CT perfusion demonstrates a significant Target Mismatch (TMM) profile, characterized by a small core infarct, a large penumbral volume (≥10 mL), and a mismatch ratio ≥1.2. This imaging profile fulfills the criteria for endovascular thrombectomy in wake-up stroke patients or patients with an unknown time of symptom onset ^[85]^. |
|  | Treatment Recommendation | Recommend EVT: Small core (<30 mL), large penumbra (>10 mL), and high mismatch ratio (>1.2). The anticipated benefits of successful vascular recanalization are high, given the substantial volume of salvageable tissue ^[85]^.  Do Not Recommend EVT: Large core volume (>50 mL), which indicates a substantial amount of non-viable tissue and suggests an unfavorable risk-benefit profile for recanalization therapy ^[86]^. |
| Part 4: Reporting | Reporting Physician | [Name, Title, Electronic Signature] |
|  | Reviewing Physician | [Name, Title, Electronic Signature] |
|  | Report Date and Time | e.g., October 12, 2025, 10:10 |

Note: This template is intended as a standardized reference tool for MeVO imaging assessment and is based on established guidelines and expert consensus. It should be adapted to local institutional protocols and imaging capabilities.
