# Supplementary material for: Evidence, strategies, and pathways for thrombectomy in medium vessel occlusion stroke: a systematic review
Source: Front Neurol. 2026 Sep 29;17:1838008. doi: 10.3389/fneur.2026.1838008 (PMC13658856; doi:10.3389/fneur.2026.1838008)
Supplement: Supplementary file 12 [file Table_5.docx]

Supplementary Table S5. A conceptual, hypothesis-generating scoring system for patient selection in MeVO thrombectomy (requires prospective validation).

Important caveat: **THIS TOOL HAS NOT BEEN EMPIRICALLY DERIVED OR VALIDATED. IT MUST NOT BE USED FOR CLINICAL DECISION-MAKING.** This scoring system is entirely conceptual and derived from author synthesis of the reviewed evidence. Its component weights have not been empirically derived or prospectively validated. It should be regarded as a hypothesis-generating framework to guide future research, and must not be used to guide clinical decision-making in its current form.

Abbreviations: sICH, symptomatic intracerebral hemorrhage; SAH, subarachnoid hemorrhage; M2–M4, segments of the middle cerebral artery; PCA, posterior cerebral artery.

| Dimension | Assessment Elements | Scoring Criteria | Score |
| --- | --- | --- | --- |
| Patient Baseline Characteristics | Age | Non-Octogenarian Elderly Patients (<80 years) | +1 |
|  |  | Octogenarian Patients (≥80 years) ^1^ | 0 |
|  |  | Nonagenarian Patients (≥90 years) ^2^ | -1 |
|  | Complications and Other Adverse Events | Comorbid Intracranial Tumor, sICH, SAH, Perforation ^2^ | Treatment should be individualized based on the specific clinical scenario. |
|  | Other Diseases | No other comorbid conditions that significantly affect longevity or the efficacy of thrombectomy are present. | +1 |
|  |  | Metastatic malignancy or other terminal illnesses with limited life expectancy, in which the overall prognosis is primarily determined by the comorbid conditions rather than the acute stroke itself ^3^. | -1 |
| Clinical Severity | NIHSS Score | NIHSS Score ≥ 5 ^4^ | +2 |
|  |  | NIHSS Score 3-5 with disabling deficits that significantly impact the patient's daily living and professional activities ^4^. | +1 |
|  |  | NIHSS score < 5 with no disabling deficits. | -1 |
|  | Relationship Between Symptoms and MeVO Location | Clinical symptoms correspond to and are fully explained by the MeVO occlusion site ^3^. | +1 |
|  |  | Symptoms are significantly more severe than expected for the given MeVO location ^3^. | -1 |
|  | Symptoms are significantly more severe than expected for the given MeVO location. | Neurological deterioration after hospital admission | +1 |
|  |  | Stable Symptoms/No Fluctuation | 0 |
| Imaging Mismatch | Ischemic Core Volume | ≤50 mL | +1 |
|  |  | 50 mL-70 mL | -1 |
|  |  | >70mL | -2 |
|  | Mismatch ratio (time-to-maximum (Tmax) >6 s cerebral volume/ischemic core volume) | ≥1.2 and mismatch volume ≥10 ml | +1 |
|  |  | No Significant Mismatch | 0 |
| Anatomy and Risks | Occluded Vessel Location | M2 Dominant Hemisphere, A2 (Current evidence is conflicting; while anatomical eloquence suggests greater potential benefit from recanalization, some data indicate dominant hemisphere lesions may be associated with poorer prognosis independent of reperfusion. The direction and magnitude of any effect on EVT benefit are uncertain.) ^5^† | +1 |
|  |  | M3/M4 Non-Dominant Hemisphere, PCA ^6^ | -1 |
|  | Vascular Path and Morphology | Smooth vascular path with no severe tortuosity or tandem lesions. | +1 |
|  |  | Smaller caliber, tortuous course, and thinner walls ^6^. | -1 |
|  | Symptom Onset to Anticipated Puncture Time | ≤6 hours ^7^ | +2 |
|  |  | 6-24 hours ^8^ | +1 |
|  |  | >24hours | -1 |

The assigned weights (+2, +1, 0, −1, −2) represent the authors' qualitative synthesis of the relative prognostic importance of each factor based on the studies included in this review. They do not reflect quantitative meta-analytic estimates and are intended solely for illustrative purposes within this conceptual framework. The NIHSS ≥5 threshold for the +2 score was chosen as an intermediate value that synthesizes the enrollment criteria of the three pivotal RCTs: ESCAPE-MeVO (NIHSS >5), DISTAL (NIHSS ≥4), and ORIENTAL-MeVO (NIHSS ≥6). This threshold has not been empirically validated as an optimal cut-off and should be interpreted with appropriate caution.

† This assigned weight represents the authors' hypothesis that the functional importance of eloquent regions in the dominant hemisphere (e.g., language, motor praxis) may confer a greater potential for recovery if rapid reperfusion is achieved. However, this is a major assumption with significant uncertainty. Contradictory evidence suggests that dominant hemisphere stroke may be independently associated with worse functional outcomes due to the impact on more critical neurological functions. This represents one of the most critical areas requiring prospective empirical validation within this conceptual framework.

**References**

1.	Winkelmeier L, Kniep H, Faizy T, et al. Age and Functional Outcomes in Patients With Large Ischemic Stroke Receiving Endovascular Thrombectomy. *JAMA Netw Open* 2024;7(8):e2426007.

2.	Derraz I, Benali A, Ahmed R, et al. Impact of endovascular reperfusion therapy in nonagenarians with anterior circulation large-vessel ischaemic stroke. *Age Ageing* 2021;50(3):787-94.

3.	Ospel JM, Nguyen TN, Jadhav AP, et al. Endovascular Treatment of Medium Vessel Occlusion Stroke. *Stroke* 2024;55(3):769-78.

4.	Ospel JM, Dowlatshahi D, Demchuk A, et al. Endovascular treatment to improve outcomes for medium vessel occlusions: The ESCAPE-MeVO trial. *Int J Stroke* 2024;19(9):1064-70.

5.	Qu Y, Jin H, Abuduxukuer R, et al. The association between serum S100β levels and prognosis in acute stroke patients after intravenous thrombolysis: a multicenter prospective cohort study. *BMC Med* 2024;22(1):304.

6.	Nogueira RG, Doheim MF, Al-Bayati AR, et al. Distal Medium Vessel Occlusion Strokes: Understanding the Present and Paving the Way for a Better Future. *J Stroke* 2024;26(2):190-202.

7.	Roaldsen MB, Jusufovic M, Berge E, et al. Endovascular thrombectomy and intra-arterial interventions for acute ischaemic stroke. *Cochrane Database Syst Rev* 2021;6(6):Cd007574.

8.	Jadhav AP, Desai SM, Jovin TG. Indications for Mechanical Thrombectomy for Acute Ischemic Stroke: Current Guidelines and Beyond.*Neurology* 2021;97(20 Suppl 2):S126-s36.
