# Supplementary material for: Evidence, strategies, and pathways for thrombectomy in medium vessel occlusion stroke: a systematic review
Source: Front Neurol. 2026 Sep 29;17:1838008. doi: 10.3389/fneur.2026.1838008 (PMC13658856; doi:10.3389/fneur.2026.1838008)
Supplement: Supplementary file 13 [file Table_6.docx]

Supplementary Table S6. Definitions and clinical significance of key efficacy and safety metrics for MeVO thrombectomy.

Abbreviations: mRS, modified Rankin Scale; eTICI, expanded Thrombolysis in Cerebral Infarction; FPE, first-pass effect; sICH, symptomatic intracranial hemorrhage; SAH, subarachnoid hemorrhage; EVT, endovascular thrombectomy.

| Dimension | Validation Metrics | Definition and Interpretation |
| --- | --- | --- |
| Clinical Benefits | 90-day Functional Independence (mRS 0-2) | • Indicates the ability to perform activities of daily living (e.g., walking, eating) without assistance at 90 days post-procedure ^1^.  • Represents the ultimate patient-centered endpoint for evaluating the efficacy of EVT. |
| Technical Success Rate | Successful Recanalization (eTICI ≥2b50) | • Assessed using the expanded TICI (eTICI) scale, evaluating the restoration of antegrade reperfusion in the target territory.  • A grade of 2b50 or greater indicates successful reperfusion of ≥50% of the involved ischemic territory and is associated with higher rates of favorable clinical outcomes. |
|  | First-Pass Effect (FPE) Rate | • Defined as achieving near-complete or complete reperfusion (eTICI 2c–3) with a single thrombectomy device pass, without the use of rescue therapy.  • A high FPE rate is desirable as it minimizes endothelial injury, procedural time, and clot fragmentation. |
|  | Puncture-to-reperfusion time | • A critical workflow metric; the benefit of thrombectomy is highly time-dependent.  •Shorter times are consistently associated with improved functional outcomes, where every one-minute reduction can translate into additional days of disability-free survival^2^. |
| Procedural Safety | Symptomatic Intracranial Hemorrhage (sICH) | • A serious complication defined per the Heidelberg Bleeding Classification as any intracranial hemorrhage associated with a ≥4-point increase in NIHSS score or leading to death, and directly attributable to the hemorrhage ^3^.  • Significantly associated with poor functional outcomes and increased mortality. |
|  | Vascular Perforation Rate | • Iatrogenic rupture of an intracranial artery during the procedure, confirmed angiographically by active contrast extravasation.  • Perforation carries a mortality rate exceeding 50% in some series, and active bleeding persisting beyond 20 minutes further worsens survival (HR 1.797) ^4^. |
|  | Subarachnoid Hemorrhage (SAH) | • May occur due to vessel perforation, wire injury, or avulsion of small perforating arteries during device manipulation ^5^.  • Often detected post-procedurally as subarachnoid hyperdensity (SA-HD) on CT, with incidence varying based on detection methodology ^6^. |
|  | distal embolization | • Intraprocedural migration of thrombus fragments to previously uninvolved, distal vascular territories, potentially creating new occlusions.  • Significantly associated with neurological deterioration, and its occurrence is linked to thrombus fragility, device technique, and patient-specific factors ^7^.  • Distal embolization is significantly associated with neurological deterioration (p<0.01), potentially hindering complete recanalization and contributing to unfavorable functional outcomes. Its occurrence is linked to thrombus characteristics, procedural techniques, and patient-specific factors, and may significantly impact prognosis ^5^. |
| Ultimate Endpoint | 90-Day All-Cause Mortality | • The proportion of deaths from any cause within 90 days of treatment.  • Provides a definitive safety and effectiveness measure, capturing both short-term mortality and delayed deaths from complications of disability, while remaining attributable to the index stroke and its consequences. |

**References**

1.	Salim HA, Yedavalli V, Musmar B, Adeeb N, K ELN, Henninger N, et al. Endovascular therapy versus best medical management in distal medium middle cerebral artery acute ischaemic stroke: a multinational multicentre propensity score-matched study. Journal of neurology, neurosurgery, and psychiatry. 2025;96(3):239-48.

2.	Desai SM, Psychogios M, Khatri P, Jovin TG, Jadhav AP. Direct Transfer to the Neuroangiography Suite for Patients With Stroke. Stroke. 2023;54(6):1674-84.

3.	von Kummer R, Broderick JP, Campbell BC, Demchuk A, Goyal M, Hill MD, et al. The Heidelberg Bleeding Classification: Classification of Bleeding Events After Ischemic Stroke and Reperfusion Therapy. Stroke. 2015;46(10):2981-6.

4.	Schulze-Zachau V, Rommers N, Ntoulias N, Brehm A, Krug N, Tsogkas I, et al. Insights into vessel perforations during thrombectomy: Characteristics of a severe complication and the effect of thrombolysis. European stroke journal. 2025;10(1):63-73.

5.	Salsano G, Pracucci G, Mavilio N, Saia V, Bandettini di Poggio M, Malfatto L, et al. Complications of mechanical thrombectomy for acute ischemic stroke: Incidence, risk factors, and clinical relevance in the Italian Registry of Endovascular Treatment in acute stroke. International journal of stroke : official journal of the International Stroke Society. 2021;16(7):818-27.

6.	Renú A, Laredo C, Rodríguez-Vázquez A, Santana D, Werner M, Llull L, et al. Characterization of Subarachnoid Hyperdensities After Thrombectomy for Acute Stroke Using Dual-Energy CT. Neurology. 2022;98(6):e601-e11.

7.	Wong GJ, Yoo B, Liebeskind D, Baharvahdat H, Gornbein J, Jahan R, et al. Frequency, Determinants, and Outcomes of Emboli to Distal and New Territories Related to Mechanical Thrombectomy for Acute Ischemic Stroke. Stroke. 2021;52(7):2241-9.
