# Supplementary material for: Evidence, strategies, and pathways for thrombectomy in medium vessel occlusion stroke: a systematic review
Source: Front Neurol. 2026 Sep 29;17:1838008. doi: 10.3389/fneur.2026.1838008 (PMC13658856; doi:10.3389/fneur.2026.1838008)
Supplement: Supplementary file 14 [file Table_7.docx]

Supplementary Table S7. Summary of device evidence for MeVO thrombectomy techniques.

Abbreviations: eTICI, expanded Thrombolysis in Cerebral Infarction; mRS, modified Rankin Scale; FPE, first-pass effect; ICH, intracranial hemorrhage; sICH, symptomatic intracranial hemorrhage; ADAPT, A Direct Aspiration First-Pass Technique; SAVE, Stent-retriever Assisted Vacuum-locked Extraction; SAH, subarachnoid hemorrhage; CA, contact aspiration; SR, stent retriever.

| Device/Technique Category | Generation & Key Characteristics | Key Efficacy Metrics | Representative Key Studies |
| --- | --- | --- | --- |
| SR (2–3 mm Diameter Specific Series)  (e.g., 3mm Trevo SR, Solitaire X 3mm, APERIO Hybrid 17) | Accessibility: For distal medium vessel occlusions (DMVO) in the posterior circulation, SRs combined with CA resulted in higher reperfusion rates (52% vs 35%) ^1^.  Specificity: The core function of different SRs is focused on vascular recanalization and clinical improvement ^2^. | • eTICI ≥2b: 87.9%  • mRS 0-2: 41.8%  •Procedure-Related Adverse Events: 24.2%  • FPE: 62%  • ICH Risk: 48.0% | ASTER2 ^3^ |
| Distal Aspiration | Accessibility: First-line treatment demonstrated higher reperfusion rates across all occlusion segments and shorter procedure times ^4^.  Efficacy: In posterior circulation LVO stroke, showed shorter procedure times, better reperfusion, and better clinical outcomes vs. SR thrombectomy ^1^. | • eTICI ≥2b: 87.0%  • mRS 0-2: 44.0%  • FPE: 37.3%  • sICH Risk: 3.0%  • Procedure-Related Adverse Events: 49.0%  ^1^ | MR. CLEAN  • Shorter procedure time ^1^.  • Did not significantly improve reperfusion rates.  • Reduced unnecessary passes and immediate complications ^5^. |
| SR + Intermediate CA(Combined Technique) | Accessibility: Compared to SR alone, initial thrombectomy with a combined technique did not significantly improve near-complete or complete reperfusion rates at the end of the procedure ^6^.  Efficacy: Resulted in better final angiographic outcomes ^6^. | • eTICI ≥2b: 86.7%  • mRS 0-2: 38%  • Procedure-Related Adverse Events: 24.7%  • FPE: 37.3%  • ICH Risk: 46.0% | ASTER2 ^3^ |
| SAVE Technique | Strategy: Using the SAVE technique as first-line treatment might improve recanalization rates, rather than aspiration alone ^7^. | • eTICI ≥2b: 95%  • FPE: 51%  • Distal Embolization: 44% ^7^ | ASTER‑2  •Controversy remains on superiority.  •vs. ADAPT: lower distal embolization, higher first-pass success, fewer passes, shorter procedure times ^3^. |
| Double SR Technique | Strategy: Used as an effective rescue technique when single SR thrombectomy fails, showing high successful final reperfusion rates with low complication rates ^8^. | •mTICI≥2b: 92.60%  • mRS 0-2: 41.8%  • Final Recanalization Rate: 92.6%  • FPE: 76.6%  • SAH: 1.56%  •sICH Risk: 5.5% ^8^ | Selected Systematic Reviews and Meta-Analyses |
| QUATTRO / Four-Axis Technique | Strategy: Reduces the need for oversized aspiration catheters; effectively prevents vessel straightening, avoids avulsing small perforators, and limits collapse of smaller arteries ^9^. | • mTICI ≥2b: 98.0%  • mRS 0-2: 58.7%  • Final Recanalization Rate: 98.0%  • FPE: 76.6%  • Perforation: 2.0%  • SAH: 3.5%  • sICH Risk: 3.6% ^10^ | Selected Retrospective Studies ^10^ |

Note: This table provides a broad, contextual overview of common device categories and techniques discussed in the neurointerventional literature, intended to aid interpretation of device evolution in MeVO. It is not restricted to the studies included in this systematic review and includes key representative studies (e.g., ASTER2, MR CLEAN) conducted predominantly in large vessel occlusion (LVO) populations. The performance metrics cited are for general reference and were not derived or validated within this review's specific MeVO-focused analytic framework. For the specific outcomes of EVT versus medical management in MeVO, readers are referred to the primary results and analyses of the studies included in this review.

**References**

1.	Meyer L, Stracke P, Wallocha M, et al. Aspiration Versus Stent Retriever Thrombectomy for Distal, Medium Vessel Occlusion Stroke in the Posterior Circulation: A Subanalysis of the TOPMOST Study. *Stroke* 2022;53(8):2449-57.

2.	Nogueira RG, Lobsien D, Klisch J, et al. Thrombectomy With the pRESET vs Solitaire Stent Retrievers as First-Line Large Vessel Occlusion Stroke Treatment: A Randomized Clinical Trial. *JAMA Neurol* 2024;81(2):170-8.

3.	Sgreccia A, Desilles JP, Costalat V, et al. Combined Technique for Internal Carotid Artery Terminus or Middle Cerebral Artery Occlusions in the ASTER2 Trial. *Stroke* 2024;55(2):376-84.

4.	Bernsen MLE, Goldhoorn RB, Lingsma HF, et al. Importance of Occlusion Site for Thrombectomy Technique in Stroke: Comparison Between Aspiration and Stent Retriever. *Stroke* 2021;52(1):80-90.

5.	Hassan AE, Abraham MG, Blackburn S et al. Association of Reperfusion and Procedural Characteristics with Endovascular Thrombectomy Outcomes in Large Core Stroke: Sub-Analysis from the SELECT2 Trial. *Ann Neurol* 2024.

6.	Lapergue B, Blanc R, Costalat V, et al. Effect of Thrombectomy With Combined Contact Aspiration and Stent Retriever vs Stent Retriever Alone on Revascularization in Patients With Acute Ischemic Stroke and Large Vessel Occlusion: The ASTER2 Randomized Clinical Trial. *JAMA* 2021;326(12):1158-69.

7.	Pero G, Dória HM, Piano M, et al. Intracranial Carotid Occlusions : ADAPT versus SAVE and the role of Balloon Guide Catheters. *Clin Neuroradiol* 2023;33(3):825-31.

8.	Hofmeister J, Brina O, Bernava G, et al. Double Stent Retriever Technique for Mechanical Thrombectomy: A Systematic Review and Meta-Analysis. *AJNR Am J Neuroradiol* 2024;45(8):1031-7.

9.	Psychogios MN, Tsogkas I, Blackham K, et al. The Quattro Technique for Medium Distal Vessel Occlusion Stroke. *Clin Neuroradiol* 2024;34(1):257-62.

10.	Aebischer V, Brehm A, Rommers N, et al. First-line Aspiration Thrombectomy with the RED43 Catheter for Acute Ischemic Stroke due to Medium or Distal Vessel Occlusion. *Clin Neuroradiol* 2025.
