# Supplementary material for: Evidence, strategies, and pathways for thrombectomy in medium vessel occlusion stroke: a systematic review
Source: Front Neurol. 2026 Sep 29;17:1838008. doi: 10.3389/fneur.2026.1838008 (PMC13658856; doi:10.3389/fneur.2026.1838008)
Supplement: Supplementary file 15 [file Table_8.docx]

Supplementary Table S8. Summary of efficacy and safety outcomes of included studies

Abbreviations: mTICI, modified thrombolysis in cerebral infarction; CTA, CT angiography; CTP, CT perfusion; DSA, digital subtraction angiography; FPE, first-pass effect; sICH, symptomatic intracranial hemorrhage; mRS, modified Rankin scale; OR, odds ratio; aOR, adjusted odds ratio; CI, confidence interval; MeVO, medium vessel occlusion; LVO, large vessel occlusion; DMVO, distal medium vessel occlusion; BGC, balloon guide catheter; SR, stent retriever; CA, contact aspiration; ENT, embolization to new territory; SAH, subarachnoid hemorrhage; GA, general anesthesia; CS, conscious sedation; NIHSS, National Institutes of Health Stroke Scale; ASPECTS, Alberta Stroke Program Early CT Score; HIR, hypoperfusion intensity ratio; FIV, final infarct volume; NCCT, non-contrast CT; BMM/BMT, best medical management/therapy; IVT, intravenous thrombolysis; EVT, endovascular therapy/mechanical thrombectomy; SITS-MOST, Safe Implementation of Thrombolysis in Stroke-Monitoring Study.

Note: *aOR represents results from multivariable analysis.

| Study (First Author | 90-day mRS 0–2 | TICI≥2b | FPE | sICH | 90-day mortality | SAH | ENT | Perforation | Dissection | Evidence Level |
| --- | --- | --- | --- | --- | --- | --- | --- | --- | --- | --- |
| Randomized Controlled Trials | | | | | | | | | | |
| DISTAL Investigators 2024 | Only 55.2% in combined groups | 71.7% (EVT group) | Not reported | EVT 5.9% vs BMT 2.6%; aOR 2.38 (0.44–6.14) | EVT 15.5% vs BMT 14.0%; aOR 1.17 (0.71–1.90) | Reported, OR not provided | 6.3% (EVT group) | 3.0% (EVT group) | Not reported | High |
| ESCAPE-MeVO Investigators 2024 | EVT 54.1% vs Control 58.8%; aRR 0.92 (0.80–1.05) | 75.1% (EVT group) | Not reported | EVT 5.4% vs Control 2.2%; OR not provided | EVT 13.3% vs Control 8.4%; aHR 1.82 (1.06–3.12) | EVT 7.4% vs Control 0.7% | Not reported | Rare (5 patients) | Rare (included in 5 patients) | High |
| Hu W, 2026 (ORIENTAL-MeVO) | EVT 58.6% vs Control 46.6%; aRR 1.24 (1.07–1.44), P=0.004 | 74.4% (eTICI 2b50-3) | Not reported | EVT 4.7% vs Control 2.2%; aRR 2.21 (0.87–5.63) | EVT 11.1% vs Control 10.2%; aRR 1.11 (0.70–1.76) | Not reported | Not reported | Not reported | Not reported | High |
| Clarençon F, 2024 (DISCOUNT) | Not reached; trial terminated early after interim analysis showed harm signal (worse functional outcomes in EVT arm) | Not fully reported | Not reported | Not fully reported; interim analysis indicated increased sICH in EVT arm | Not fully reported; interim analysis indicated higher mortality in EVT arm | Not reported | Not reported | Not reported | Not reported | High (RCT — terminated early) |
| Observational Studies | | | | | | | | | | |
| Abecassis IJ 2024 | 64.60% | Not reported | Not reported | 6.9% (sICH/PH2) | 8.80% | Not reported | Not reported | Not reported | Not reported | Moderate |
| Alessiani M 2025 | 3m mRS>2: MT 49.1% vs IVT 27% (unadjusted P=0.020); after PSM P=0.270 | 95% (MT group) | Not reported | HT (not strictly sICH): MT 33.8% vs IVT 20.8% (PSM P=0.260) | MT 11.8% vs IVT 14.5% (PSM P=0.813) | Not reported | Not reported | Not reported | Not reported | Moderate |
| Dmytriw AA 2024 | aOR 1.37 (0.99–1.89) for MT+IVT | aOR 1.64 (0.99–2.73) | aOR 1.27 (0.90–1.79) | Not specifically reported (any ICH aOR 1.25, P=0.21) | aOR 0.78 (0.49–1.24) | Not reported | Not reported | Not reported | Not reported | Moderate |
| Gaub M 2025 | Aspiration 49.9% vs SR/Combined 44.0%; aOR 1.27 (1.03–1.57) | Not reported | Complete FPE: Asp 35.0% vs SR/Comb 27.6% | Asp 3.5% vs SR/Comb 6.2%; OR 0.55 (0.33–0.91) | Asp 19.1% vs SR/Comb 17.9%; OR 1.09 (0.83–1.42) | Not reported | Not reported | Not reported | Not reported | Moderate |
| Grossberg JA 2023 | ADAPT 45% vs SRT/PC 47% (p>0.05) | Not reported | Not reported | ADAPT 4% vs SRT/PC 9% (p<0.01) | Not significantly different | Not reported | Not reported | Not reported | Not reported | Moderate |
| Ishiguro T 2024 | 90d good outcome: Tron 32.9% vs Conv 25.6% (p=0.33) | Tron 92.1% vs Conv 90.1% | Tron 48.7% vs Conv 51.2% | Not reported (focused on SAH) | Not reported | Tron 6.6% vs Conv 19.8% (p=0.012) | Not reported | Not reported | Not reported | Serious |
| Jing XZ 2025 | 60.9% (EVT group); aRR 1.39 (1.14–1.69) | 85.80% | Not reported | EVT 11.5% vs SMM 10.4%; aRR 1.24 (0.92–1.66) | EVT 8.3% vs SMM 8.9%; aRR 0.74 (0.51–1.12) | Not reported | Not reported | Not reported | Not reported | Moderate |
| Meyer L 2024 | Not reported | Not reported | ~45% | LA±CS 2.5% vs GA 1.8% | LA±CS 15.7% vs GA 17.2% | Not reported | Not reported | Not reported | Not reported | Moderate |
| Meyer L 2021 | EVT 76.6% vs Medical 75.4% (p=0.87) | 87.40% | 45.50% | Both groups: 4.3% | EVT 11.8% vs Medical 15.8% (p=0.40) | Not separately reported | 3.50% | 1.40% | 2.80% | Moderate |
| Meyer L 2023 | After PSM: MT 49% vs BMT 49% (p=0.99) | 81% | 41% | 2% (both groups) | After PSM: MT 22% vs BMT 31% (p=0.36) | Not reported | 1.00% | 4.30% | Not reported | Moderate |
| Meyer L 2022 | Asp 60.5% vs SR 68.6% (mRS 0-1) | Not reported | Asp 53.7% vs SR 44.0% | Asp 2.4% vs SR 3.0% | Asp 13.2% vs SR 10.4% (p=0.750) | Not reported | Asp 2.4% vs SR 4.0% | Not reported | Asp 4.9% vs SR 4.0% | Moderate |
| Mohammaden MH 2025 | GA 45.5% vs non-GA 43.6% | GA 90.2% vs non-GA 78.7% | GA 23% vs non-GA 26.2% | GA 15.5% vs non-GA 4.5% (OR 3.24, P=0.09) | GA 23.6% vs non-GA 20% (OR 1.43, P=0.52) | Not reported | Not reported | Not reported | Not reported | Moderate |
| Mohammaden MH 2024 DUSK | EVT 53.6% vs MM 60.6% | 89.40% | Not reported | EVT 5.6% vs MM 6.3% (aOR 0.57, P=0.277) | EVT 17.3% vs MM 9.2% (aOR 1.73, P=0.173) | Not reported | Not reported | Not reported | Not reported | Moderate |
| Nguyen TN 2024 PLATO | Overall 50.0% | 81% | SR 62.8%, CA 42.5%, Combined 39.6% | Overall 5.6% | Overall 10.9% | Not reported | Not reported | Not reported | Not reported | Moderate |
| Orscelik A 2024 | BGC 38.6% vs non-BGC 45.2% (aOR 0.89, P=0.60) | Not reported | Not reported | BGC 4.2% vs non-BGC 5.1% (aOR 0.73, P=0.40) | Discharge mortality: BGC 8.9% vs non-BGC 13.5% (aOR 0.47, P=0.03) | Not reported | Reduced with BGC (aOR 0.41) | Not reported | Not reported | Moderate |
| Palmisano V 2025 | DA 63.7% vs CT 36.1% (P=0.001) | DA 89.5% vs CT 71.1% (P=0.003) | mTICI 3: DA 40.0% vs CT 10.5% (P<0.001) | PH (surrogate): DA 4.2% vs CT 10.5% (P=0.136) | DA 9.9% vs CT 27.8% (P=0.004) | Not reported | Not reported | Not reported | Not reported | Moderate |
| Pérez‑García C 2020 | BEMP 50.9% vs mini SR 53.1% (p=0.831) | BEMP 78.6% vs SR 78.0% | BEMP 57.1% vs SR 34.0% (P=0.017) | BEMP 1.9% vs mini SR 12.8% (P=0.038) | BEMP 20.8% vs mini SR 22.4% (P=0.835) | 23.1–28.2% | BEMP 1.8% vs SR 12.0% | Not reported | Not reported | Moderate |
| Rizzo F 2024 | EVT-only 31.6% vs IVT 61% | EVT-only 56.4% | Not reported | Overall 1.1% | Overall 2.2% | 1.70% | Not reported | Not reported | Not reported | Moderate |
| Rosi A 2024 | 3D-RA 49.1% vs Control 45% (P=0.834) | 3D-RA 100% vs Control 74.4% | Not reported | 3D-RA 5.3% vs Control 2.3% (P=0.697) | Not explicitly reported | Not reported | Not reported | Not reported | Not reported | Moderate |
| Saber H 2022 | Weighted: EVT 51.7% vs MM 50.0%; aOR 1.36 (0.84–2.19) | 81.20% | 56.60% | EVT 4.0% vs MM 3.1% (P=0.90) | EVT 18.7% vs MM 15.7%; aOR 1.24 (0.63–2.43) | Not reported | Not reported | Not reported | Not reported | Moderate |
| Salim HA 2026 | mRS 0-1: IAT 38% vs MT 21% (P=0.20) | MT 90% vs IAT 68% (P=0.02) | Not reported | IAT 0% vs MT 6.7% (P=0.60) | IAT 13% vs MT 17% (P>0.99) | Not reported | Not reported | MT 1.9% vs IAT 0% | Not reported | Moderate |
| Salim HA, 2024 (MT-IVT vs IVT) | MT-IVT 62% vs IVT 63%; aOR 1.00 (0.66–1.51) | 91% | Not reported | MT-IVT 8.3% vs IVT 6.9% (aOR 0.78, P=0.44) | MT-IVT 14% vs IVT 11% (aOR 0.75, P=0.30) | Not reported | Not reported | Not reported | Not reported | Moderate |
| Salim HA, 2025 (EVT vs BMM) | EVT 59% vs BMM 59%; aOR 1.00 (0.67–1.50) | 90% | 35% | EVT 8.4% vs BMM 3.0% (aOR 3.56, P<0.001) | EVT 13% vs BMM 13% (aOR 1.48, P=0.15) | EVT 9.0% vs BMM 1.7% | Not reported | Not reported | Not reported | Moderate |
| Salsano G 2024 | EVT 74.0% vs IVT 74.6% | 90% | 46% | EVT 6.0% vs IVT 9.4% (aOR 0.49, P=0.38) | EVT 16.0% vs IVT 20.3% (aOR 0.50, P=0.19) | 8% vs 12.5% | Not reported | Not reported | Not reported | Moderate |
| Siegler JE 2025 | Trevo 52.5% vs Other 46.4% (aOR 1.32, P=0.20) | 89.50% | Not reported | Not specifically (PH: Trevo 9.5% vs Other 7.9%) | Trevo 19.9% vs Other 20.7% (aOR 0.93) | Not reported | Not reported | Not reported | Not reported | Moderate |
| Siegler JE 2023 | No significant difference | Asp 90.2% vs SR 82.7% | Asp 58.0% vs SR 54.8% | Not specifically (Any ICH: Asp 25.3% vs SR 34.2%) | Asp 18.6% vs SR 18.5% (aOR 0.96) | Not reported | Not reported | Not reported | Not reported | Moderate |
| Zhang K 2026 | 62.7% (EVT) vs 52.8% (SMM); aRR 2.35 (1.63–3.39) | 85.50% | Not reported | No significant difference (PSM aRR 1.12, P=0.793) | PSM aRR 0.42 (0.14–1.30) | Not reported | Not reported | Not reported | Not reported | Moderate |
| Nedelcu S 2023 | EVT 69.2% vs BMT 70.7% (OR 0.56, P=0.341) | 87.20% | Not reported | PH: EVT 12.8% vs BMT 9.8% (P=0.879) | EVT 10.3% vs BMT 4.9% (P=0.426) | Not reported | Not reported | Not reported | Not reported | Moderate |
| Shimonaga K 2025 | Overall 50.8% | 75% | Overall 29% | Overall 2.2% | Not reported | 28.40% | Not reported | Not reported | Not reported | Moderate |
| Hu D 2026 | IPTW: EVT 68.0% vs Conservative 67.6% (P=0.998) | 74.20% | Not reported | EVT 8.2% vs Conservative 0% (P=0.002) | EVT 6.2% vs Conservative 8.3% (P=0.721) | Not reported | Not reported | Not reported | Not reported | Moderate |
| Alawieh AM 2023 | Overall ~44% | 75% | Not reported | ~5-6% | ~19% (overall LVO/MeVO/DVO) | Not reported | Not reported | Not reported | Not reported | Moderate |
| Elhorany M 2022 | After PSM: MT+BMT 53.6% vs BMT 57.1% (OR 0.87, P=1.00) | 90.90% | Not reported | After PSM: MT+BMT 3.6% vs BMT 0% | After PSM: MT+BMT 7.1% vs BMT 10.7% (OR 0.64, P=1.00) | Not reported | Not reported | Not reported | Not reported | Moderate |
| Nagel S 2020 | Good outcome: EVT 73.3% vs IVT 87.5% (P=0.14) | 86.60% | Not reported | EVT 13.3% vs IVT 1.6% (P=0.01) | EVT 13.3% vs IVT 1.6% (P=0.04) | Not reported | Not reported | Not reported | Not reported | Moderate |
| Mehta A 2025 | mRS 0-3: CS 41.7% vs GA 34.4% (P=0.12) | CS 79.8% vs GA 72.9% (P=0.33) | CS 31.1% vs GA 24.0% (P=0.29) | PH1/2: CS 2.2% vs GA 4.2% (P=0.51) | CS 10.96% vs GA 19.79% (P=0.04 unadjusted; aOR 0.51, P=0.16) | CS 10.96% vs GA 10.42% | Not reported | Not reported | Not reported | Moderate |
| Valente I 2024 | mRS 0-1: GA 74.5% vs CS/LA 72.3% (aOR 1.12, P=0.815) | GA 89.4% vs CS/LA 83.0% (P=0.374) | Not reported | Not reported (sSAH: GA 2.2% vs CS/LA 13.5%, P=0.082) | GA 8.5% vs CS/LA 4.3% (aOR 2.09, P=0.408) | Not reported | Not reported | Not reported | Not reported | Moderate |
| Hofmeister J 2025 | No significant difference | CE-CBCT 100% vs 3DRA 74.4% (P<0.001) | CE-CBCT 65.7% vs 3DRA 37.8% (P=0.02) | Not reported | Not reported | Not reported | 2.7–2.8% | 0% | 0% | Moderate |
| Berberich A 2025 | non-GA 50.0% vs GA 47.7% (OR 0.84, P=0.49) | GA 83.6% vs non-GA 73.6% (aOR 1.70, P=0.005) | Not reported | non-GA 7.8% vs GA 5.0% (OR 0.54, P=0.31) | non-GA 10.9% vs GA 11.6% (OR 1.18, P=0.64) | Not reported | Not reported | Not reported | Not reported | Moderate |
| MAD-MT HIR Analysis | 50% (73/147) | Not reported | Not reported | Not reported | 12% (18/147) | Not reported | Not reported | Not reported | Not reported | Moderate |
| Virtanen et al 2025 | Not reported | 64.0% vs 62.5% | 24% | OR 3.32 (1.04-10.64) | 15% vs 16% | 17% | Not reported | Not reported | Not reported | Moderate |
| Settecase et al 2024 | Not reported | Primary 97%, Secondary 86% | Primary 66%, Secondary 69% | 3.3% (3/92) | In-hospital: 8.7% | 1.10% | 1.10% | 1.10% | Device 0%, ICA 1 case | Low |
| Schwartzmann et al 2024 | mRS 0-1: 39% | 96% vs 87.5% | Not reported | 3% (2/66) | Not reported | Not reported | Not reported | Not reported | Not reported | Moderate |
| Yedavalli et al 2024 DMVO | aOR 1.00 (0.67-1.50) | 90% | 35% | aOR 3.56 (1.69-7.48) | aOR 1.48 (0.87-2.51) | aOR 6.71 (2.53-17.8) | Not reported | Not reported | Not reported | Moderate |
| Salim et al 2024 Aspirin | aOR 1.89 (1.14-3.12) | No significant difference | Not reported | aOR 0.92 (0.60-1.43) | aOR 0.56 (0.32-1.00) | No significant difference | Not reported | Not reported | Not reported | Moderate |
| Salim et al 2024 ENT | aOR 0.87 (0.48-1.60) | aOR 0.41 (0.23-0.72) | Not reported | aOR 1.51 (0.67-3.42) | aOR 1.80 (0.91-3.58) | aOR 1.10 (0.42-2.86) | Incidence 2.9% | Not reported | Not reported | Moderate |
| Loehr et al 2024 ASSIST | 65.00% | 93.50% | 43.10% | 1.30% | 9.00% | Not reported | Not reported | 1 case (CA) | Not reported | Moderate |
| Mohammaden et al 2024 BGC | IPTW aOR 4.31 (1.66-11.19) | IPTW aOR 1.02 (0.35-2.96) | IPTW aOR 5.63 (2.43-13.10) | IPTW aOR 0.75 (0.20-2.76) | IPTW aOR 0.32 (0.11-0.98) | Not reported | BGC 4.2%, non-BGC 1.6% | BGC 1, non-BGC 2 | BGC 0, non-BGC 1 | Moderate |
| Madaelil et al 2024 | 61.9% (single-arm) | 87.7% (single-arm) | 38% (core lab) | 1.5% (single-arm) | 4.6% (single-arm) | Not reported | Not reported | Not reported | Not reported | Low |
| Regenhardt et al 2024 | Not reported | aOR 0.18 (0.08-0.40) | aOR 0.27 (0.10-0.61) | Not reported | Not reported | Not reported | Not reported | Not reported | Not reported | Moderate |
| He et al 2025 | aOR 1.70 (1.01-2.89) | PSI coefficient 0.10 (P<0.001) | Not reported | 7.10% | Not reported | Not reported | Not reported | Not reported | Not reported | Moderate |
| Hansen et al 2025 | aOR 0.93 (0.67-1.30) | Primary 85%, Secondary 82% | Not reported | Primary 3.3%, Secondary 4.3% | Not reported | Not reported | Not reported | Not reported | Not reported | Low |
| MAD-MT Registry 2024 | 64% | 85% | 27% | 6.20% | 11% | Not reported | Not reported | Not reported | Not reported | Moderate |
| Garcia et al 2024 | pMVO 74.1% vs dMVO 73.3% | pMVO 96% vs dMVO 91% | pMVO 46.3% vs dMVO 84.4% | pMVO 0.6% vs dMVO 0% | pMVO 5.9% vs dMVO 8.8% | Overall 8.1% | Not reported | Not reported | Not reported | Moderate |
| Salim et al 2024 EVT Failure | aOR 0.50 (0.29-0.85) | EVT failure: all mTICI <2b | EVT failure: all mTICI <2b | aOR 4.32 (1.84-10.10) | aOR 1.18 (0.67-2.05) | Not reported | Not reported | Not reported | Not reported | Moderate |
| REVISAR Registry 2024 | 79.00% | 95.80% | 63.90% | 0% | 4.2% (in-hospital) | 12.6% (all minor) | 1.70% | Not reported | 1.7% (non-target) | Low |
| CTP-Guided MeVO Study 2024 | Overall aOR 1.76 (0.79-3.95) | 61% | Not reported | aOR 1.89 (0.45-7.92) | Not reported | Not reported | Not reported | Not reported | Not reported | Moderate |
| Amuluru et al 2024 | Monoplane 54% vs Biplane 42% | Monoplane 91% vs Biplane 86% | Not reported | Monoplane 4% vs Biplane 7% | Not reported | Monoplane 7% vs Biplane 14% | Not reported | 0% vs 0% | 0% vs 0% | Moderate |
| MAD-MT Registry 2024 (n=1568) | Overall 52% | 86% | 33% | Not reported | 18% | 9.40% | 3.40% | 4.30% | 0.90% | Moderate |
| Yedavalli et al 2024 CBV | CBV ≥0.7 better (OR 2.27) | 100% (by inclusion) | Not reported | Not reported | Not reported | Not reported | Not reported | Not reported | Not reported | Moderate |
| Mujanovic et al 2024 | SH II-IV vs SH 0-I: aOR 2.2 (1.1-4.3) | SH group lower | Not reported | Not reported | SH grade II: aOR 3.7 (1.4-9.9) | Incidence 45% | Not reported | Not reported | Not reported | Moderate |
| Schäfer et al 2024 | MeVO 25.7% vs LVO 10.9% | Not reported | Not reported | Not reported | MeVO 50.5% vs LVO 53.0% | Primary focus | Not reported | MeVO 2.4% vs LVO 1.0% | Not reported | Moderate |
| German Stroke Registry 2024 | 36.0% vs 34.9% (aOR 0.94/yr) | 83.9% vs 85.5% (aOR 1.07/yr) | 35.8% vs 41.6% (aOR 1.06/yr) | Stable 4.4% | 25.3% vs 34.7% (aOR 1.13/yr) | Not reported | Stable 4.0% | Stable 3.4% | Not reported | Moderate |
| Guenego et al 2024 | OR 0.95 (0.67-1.35) | CS 89.4% vs GA 90.2% | CS 32.9% vs GA 33.8% | OR 5.32 (1.92-14.72) | CS 16.5% vs GA 22.6% | Not reported | Not reported | Not reported | Not reported | Moderate |
| Radu et al 2024 | FPE 72.8% vs Non-FPE 52.8% (aOR 2.20) | 100% (by inclusion) | FPE group: 100% | FPE 0.4% vs Non-FPE 1.2% | FPE 6.6% vs Non-FPE 15.7% (aOR 0.43) | Not reported | Not reported | Not reported | Not reported | Moderate |
| Nome et al 2024 | BGC 38.6% vs non-BGC 45.2% (aOR 0.89) | BGC 43.2% vs non-BGC 52.7% (aOR 0.70) | BGC 25.9% vs non-BGC 30.5% | BGC 4.2% vs non-BGC 5.1% (aOR 0.73) | In-hospital: aOR 0.47 (0.23-0.88) | Not reported | Reduced (aOR 0.41) | Not reported | Not reported | Moderate |
| Bo et al 2024 | aOR 0.89 (0.59-1.35) | aOR 0.70 (0.50-0.98) | aOR 0.83 (0.56-1.20) | aOR 0.73 (0.30-1.54) | Not reported | Not reported | Not reported | Not reported | Not reported | Moderate |
| Monteiro et al 2024 | 2nd vs 1st MeVO: OR 0.13 (0.03-0.46) | 92.80% | 49.3% (mFPE 69.6%) | 2.90% | Puncture-to-recanalization: aOR 0.95 (0.92-0.98) | Not reported | Not reported | Not reported | Not reported | Moderate |
| Matsukawa et al 2024 | M3 vs M2: aOR 1.05; A2 vs M2: aOR 0.30; P2 vs M2: aOR 0.64 | M3 vs M2: aOR 0.49; A2 vs M2: aOR 0.64; P2 vs M2: aOR 0.19 | Not reported | Not reported | M3 vs M2: aOR 1.02; A2 vs M2: aOR 1.08; P2 vs M2: aOR 0.51 | Not reported | Not reported | Not reported | Not reported | Moderate |
| Toledo et al 2024 | Not Applicable (Predictive Model) | Not Applicable | Not Applicable | Not Applicable | Not Applicable | Not Applicable | Not Applicable | Not Applicable | Not Applicable | Moderate |
| Kuwahara et al 2024 | aOR 0.70 (0.48-1.002) | aOR 0.60 (0.35-1.03) | aOR 1.77 (1.26-2.47) | aOR 1.80 (0.64-5.08) | aOR 0.82 (0.42-1.58) | aOR 1.80 (0.64-5.08) | Not reported | Not reported | Not reported | Moderate |
| Yedavalli et al 2024 HT | 55.3% (HT+ vs HT-: 17.0% vs 38.3%) | 100% (by inclusion) | Not reported | Not reported | Not reported | Not reported | Not reported | Not reported | Not reported | Low |
| Dmytriw et al 2024 Perforation | Perf 28.8% vs Non-perf 53.9% | Perf 56.5% vs Non-perf 88.3% | Perf 1.7% vs Non-perf 35.9% | Not reported | Perf 30.3% vs Non-perf 16.8% | Perf 32.3% vs Non-perf 8.9% | Not reported | Overall 4.8% | Not reported | Low |
| Schwarz et al 2023 | 77.1% vs 77.6% | Not reported | Not reported | 2.6% vs 3.4% | 9.8% vs 9.2% | 12.5% vs 13.3% | Not reported | Not reported | Not reported | Moderate |
| Enriquez et al 2023 | 58% vs 56% (NS) | 91% (TO group) | Not reported | 7.8% (TO group) | Not reported | Not reported | Not reported | Not reported | Not reported | Low |
| Shi et al 2023 | aOR 0.90 (0.62-1.31) | aOR 1.00 (0.51-1.93) | Not reported | aOR 0.59 (0.26-1.34) | aOR 0.83 (0.48-1.46) | Not reported | Not reported | Not reported | Not reported | Critical |
| TOPMOST 2024 | Not reported | Asp 90.2% vs SR 87.0% | Asp 53.7% vs SR 44.0% | Asp 2.4% vs SR 3.0% | Asp 13.2% vs SR 10.4% | Not reported | Asp 2.4% vs SR 4.0% | Included in vascular injury | Asp 4.9% vs SR 4.0% | Moderate |
| TOPMOST 2024 Secondary DMVO | 44.8% (overall) | 85.9% (primary endpoint) | 38.0% (overall) | 38.0% (overall) | 25.9% (overall) | Not reported | 2.8% (intraprocedural migration) | Included in vascular injury | 1.4% (intraprocedural migration) | Moderate |
| Guenego et al 2022 | HIR<0.3: 75% vs HIR≥0.3: 33% | 100% (all patients) | Not reported | 7% | 5% | Not reported | Not reported | Included in procedural complications | Included in procedural complications | Moderate |
| Roussel et al 2022 | Primary 44.1%, Secondary 39.4% | 74.70% | Not reported | 6.90% | In-hospital: 18.1% | 6.7–44.7% | Not reported | Included in procedural complications | Included in procedural complications | Moderate |
| TOPMOST 2023 | EVT 76.6% vs BMT 75.4% | 87.40% | 45.50% | Both groups 4.3% | EVT 11.8% vs BMT 15.8% | Not separately reported | 3.5% (EVT) | 1.4% (EVT) | 2.8% (EVT) | Low |
| BEMP Technique Study 2022 | BEMP 50.9% vs SR 53.1% | Final: BEMP 78.6% vs SR 78.0% | FPE: BEMP 57.1% vs SR 34.0% | BEMP 1.9% vs SR 12.8% | BEMP 20.8% vs SR 22.4% | BEMP 23.1% vs SR 28.2% | BEMP 1.8% vs SR 12.0% | Not reported | Not reported | Moderate |
| Bala et al 2024 | 59.1% vs 54.3% (aRR 1.09) | 71.7% vs 60.0% (aRR 1.29) | Not reported | 1.7% vs 2.3% | 14.2% vs 17.0% | Not reported | Not reported | 4.3% vs 5.0% | Not reported | Low |

This table provides the complete study-by-study outcome data. For a concise overview of evidence levels, see Table 7 in the main text.
