# Supplementary material for: Evidence, strategies, and pathways for thrombectomy in medium vessel occlusion stroke: a systematic review
Source: Front Neurol. 2026 Sep 29;17:1838008. doi: 10.3389/fneur.2026.1838008 (PMC13658856; doi:10.3389/fneur.2026.1838008)
Supplement: Supplementary file 16 [file Table_9.docx]

Supplementary Table S9. Standardized CT/CTA/CTP protocol for acute ischemic stroke and MeVO assessment.

Abbreviations: NCCT, non-contrast computed tomography; CTA, CT angiography; CTP, CT perfusion; MPR, multi-planar reformation; FDA, U.S. Food and Drug Administration.

| Protocol Component | Specification | Rationale & Notes |
| --- | --- | --- |
| Protocol Name | One-Stop CT Protocol (NCCT → CTA → CTP) | Streamlines workflow; a single start time is essential for calculating "door-to-puncture" times. |
| Scanner Model | A single, unified scanner model is recommended (e.g., Siemens Somatom Force 384). | Ensures data consistency; using different models for different sequences is highly unusual and raises concerns. |
| Non-Contrast CT (NCCT) | | |
| Tube Voltage | 70-150 kV (e.g., 100 kV) | Standard diagnostic range. |
| Tube Current | CARE Dose4D (automated modulation) | Optimizes radiation dose. |
| Reconstruction Slice Thickness | 5.0 mm | Standard for initial interpretation. |
| CT Angiography (CTA) | | |
| Tube Voltage | 100 kV | Standard for vascular imaging. |
| Contrast Agent | Iohexol (350 mgI/mL) | Standard iso-osmolar agent. |
| Volume/Rate | 50 mL at 5.0 mL/s | Standard bolus for arterial opacification. |
| Saline Flush | 40 mL at 5.0 mL/s | Ensures complete contrast delivery. |
| Trigger Method | Bolus-tracking in ascending aorta (threshold: 100 HU) | Standard technique for consistent arterial phase timing. |
| Reconstruction Slice Thickness | 0.75 mm | Enables high-resolution MPR and 3D reformats. |
| CT Perfusion (CTP) | | |
| Tube Voltage | 80 kV | Improves iodine contrast-to-noise ratio. |
| Tube Current-Time Product | 200 mAs | Balances image quality and radiation dose. |
| Coverage | 160 mm | Covers a substantial brain volume from vertex to skull base. |
| Temporal Sampling | 1.5 s intervals for 60 s total | Captures the first-pass kinetics of contrast necessary for parameter calculation. |
| Post-processing Software | RAPID (iSchemaView, Menlo Park, CA, USA) | FDA-cleared, widely validated software for automated core/penumbra quantification. |
